# Associations between psychedelic use and adverse outcomes in substance use disorders: a real-world EHR-based cohort study

**DOI:** 10.3389/fpsyt.2025.1648104

**Published:** 2025-10-24

**Authors:** Fares Qeadan, Ashlie McCunn, Benjamin Tingey, Paul Thielking

**Affiliations:** ^1^ Loyola University Chicago, Parkinson School of Health Sciences and Public Health, Maywood, IL, United States; ^2^ University of Utah Huntsman Mental Health Institute, Salt Lake City, UT, United States

**Keywords:** psychedelics, ketamine, substance use disorder, real-world evidence, electronic health records, drug overdose, mental health, relapse

## Abstract

**Aims:**

To examine associations between psychedelic use and adverse health outcomes, including overdose, relapse, mental health crises, and hospitalizations, among individuals with substance use disorders (SUD), and to compare these outcomes across different treatment modalities including anesthetics and outpatient SUD services.

**Design:**

Retrospective cohort study using propensity score-weighted quasi-Poisson regression models to estimate adjusted incidence rate ratios (aIRRs).

**Setting:**

Data were drawn from Oracle EHR Real-World Data™ comprising 138 U.S. health systems restricted to those ≥12 years old from January 1, 2000, to August 31, 2023.

**Participants:**

3,209,798 patients with a documented SUD diagnosis from 2000 to 2023. Patients with a prior history of psychedelic use or hallucinogen-related diagnoses were excluded. The final cohort included 8,514 new psychedelic users and over 3.2 million non-users.

**Measurements:**

Exposures were captured during a 3-month post-index period and included outpatient psychedelic prescriptions or procedures (primarily ketamine), general anesthetic outpatient prescriptions, and outpatient SUD services. Outcomes, assessed over 2 years, included SUD-related hospitalizations/emergency department (ED) visits, mental health crises, all-drug overdoses, and relapse. Propensity scores accounted for demographic, clinical, and behavioral confounders.

**Findings:**

Psychedelic use was associated with significantly reduced rates of all adverse outcomes, including all-drug overdose (aIRR F;= F;0.48; 95% CI: 0.37-0.63), relapse (aIRR F;= F;0.68; 0.60-0.77), SUD hospitalizations/ED visits (aIRR F;= F;0.76; 0.69-0.82), and mental health crises (aIRR F;= F;0.82; 0.73-0.92), compared to no treatment. The combination of psychedelics, anesthetics, and outpatient services was associated with the strongest reduction in mental health crises (aIRR F;= F;0.21; 0.06-0.77). Trends were consistent in sensitivity analyses including patients with mental health conditions and comparisons to medication-assisted treatment.

**Conclusions:**

In this large national cohort, psychedelic use, particularly when combined with anesthetic and outpatient care, was associated with reduced adverse health outcomes among people with SUD. These findings support further investigation into psychedelic-based interventions within integrated treatment frameworks.

## Introduction

In 2023, 48.5 million Americans (17.1%) aged 12 and older had a substance use disorder (SUD) (1). These chronic conditions, characterized by cycles of abstinence, use, and relapse, contribute significantly to public health burdens, with drug overdose deaths rising from 8.2 per 100,000 in 2002 to 32.6 in 2022 (2). While individualized treatment, especially medication-assisted treatment (MAT), is effective (3, 4), pharmacologic options are currently limited to alcohol, opioid, and tobacco use disorders (OUD, AUD, and TUD), with no approved therapies for marijuana, cocaine, methamphetamine, or stimulant misuse (5). Barriers to accessing treatment persist, including provider limitations (6), referral gaps (7), stigma (8, 9), and insurance restrictions (10). Despite established medications, underutilization remains a critical issue (11–14). Among 54.2 million people needing treatment in 2023, only 23.6% received it (1). This highlights the need to explore alternative or adjunctive therapies for SUD.

Psychedelics (also called hallucinogens), including lysergic acid diethylamide (LSD), psilocybin, dimethyltryptamine (DMT), and mescaline (15, 16), are increasingly studied as therapeutic agents. Compounds such as ketamine and methylenedioxymethamphetamine (MDMA), while mechanistically distinct, are grouped with psychedelics in research due to similar consciousness-altering effects. Use of hallucinogens for nonmedical purposes has grown, with 8.8 million adults reporting use in 2023, up from 5.5 million in 2019 (1, 17). A longitudinal study found LSD use rose slightly from 3.7% in 2018 to 4.2% in 2021, while psilocybin or phencyclidine (PCP) use increased more notably from 3.4% to 6.6% (18). Most psychedelic users are poly-users (19). In contrast, clinical use remains limited to research trials and specialized health care settings, where safety profiles and therapeutic potential are being actively investigated. Though adverse effects such as mental health crises and emergency visits are reported (20–22), these are generally tied to unsupervised recreational use rather than clinical application.

Conversely, early clinical studies suggest psychedelics may offer benefits for addiction treatment and hence are gaining a resurgence in interest as a therapy option (23). Psilocybin has yielded smoking abstinence rates up to 80% at six months; far surpassing traditional interventions (24–26). Population-based studies have linked psychedelic use with reduced odds of opioid dependence (27, 28), cocaine use disorder (29), and emotional distress linked to substance use (30).

Ketamine, a dissociative agent with psychedelic properties, is already used for depression (31), and is being investigated for SUD (32). Electronic Health Record (EHR) and trial data show associations with greater remission in cocaine and stimulant use disorders (33, 34). Randomized trials report higher abstinence in ketamine-treated groups for both cocaine (35) and AUD (36). Proposed mechanisms include modulation of addiction-related neurocircuitry (32, 33, 37). Anesthetic combinations involving ketamine (e.g., with midazolam or propofol) also show promise in clinical outcomes (38–41). While anesthetics have been explored for pain and psychiatric conditions (42–45), their use in SUD remains under-researched.

In 2022, the National Institute on Drug Abuse (NIDA) launched an initiative to evaluate psychedelic therapies for SUD (46). Yet to date, no large-scale study has assessed psychedelic treatments, alone or in combination with anesthetics and established outpatient interventions, using EHR data in a general SUD population. This study addresses this gap by examining the real-world clinical use of these therapies, particularly ketamine, in structured healthcare settings. As the only currently legal and scalable psychedelic-like agent, ketamine offers a uniquely practical lens through which to evaluate system-level feasibility and outcomes. Moreover, by analyzing treatment exposures from EHRs, this study complements existing psychedelic literature that relies heavily on non-medical, self-reported, or unregulated use, and provides critical data on how psychedelic treatments function in real-world, clinically supervised settings. This study will be the first to provide a robust, large-scale estimate of the overall benefit or risk of psychedelic/anesthetic compounds used in conjunction with current SUD treatment efforts among a diverse U.S. population comprising all SUD types. Such findings would further encourage efforts to study psychedelic compounds as a potential alternative treatment option for SUD, helping to overcome the many barriers to current SUD treatment options.

## Methods

### Data source

This retrospective study used de-identified records from Oracle EHR Real-World Data™ (OERWD), comprising 138 U.S. health systems as of November 2024. The dataset includes over 111 million patients and approximately 2 billion encounters. Oracle EHR Real-World Data is extracted from the electronic medical records of hospitals in which Oracle has a data use agreement. Encounters may include pharmacy, clinical and microbiology laboratory, admission, and billing information from affiliated patient care locations. All admissions, medication orders and dispensing, laboratory orders and specimens are date and time stamped, providing a temporal relationship between treatment patterns and clinical information. Oracle has established Health Insurance Portability and Accountability Act-compliant operating policies to establish de-identification for Oracle EHR Real-World Data (47).

### Sample

Eligible patients had a qualifying SUD diagnosis (International Classification of Diseases, Ninth/Tenth Revision, Clinical Modification (ICD-9/10-CM) or Systemized Nomenclature of Medicine-Clinical Terms (SNOMED CT); **Supplementary Table 1**) between January 1, 2000, and August 31, 2023, and were ≥12 years old. The index date was defined as the first inpatient or emergency department (ED) encounter with a qualifying SUD code, or the first of ≥2 qualifying encounters within two years. Exclusions included prior hallucinogen use disorder, psychedelic-related overdose, or psychedelic use based on Current Procedural Terminology (CPT) procedure codes, National Drug Code (NDC) or Multum MediSource Lexicon (MMSL) medication prescriptions, or Logical Observation Identifiers Names and Codes (LOINC) positive urine drug tests (**Supplementary Tables 1**, **2**). Patients required ≥3 months of post-index data.

### Study design

This study used a retrospective cohort design that started enrollment of patients at index SUD encounter and assessed treatment usage over an ensuing three-month baseline period. After the baseline period, patients were followed for two years to assess outcomes of interest. Patients were allowed inclusion until August 31, 2023, to ensure those lastly recruited had at least three more months to assess baseline treatments, and then a following year of follow-up ending at data refresh in November 2024. Thus, those lastly recruited only had one year of possible follow-up, which was done to maximize the cohort sample. Sensitivity analyses assessed robustness among those with two full years of follow-up. Outcomes were measured using person-months from follow-up start to maximum discharge.

### Outcomes

Primary outcomes included SUD-related hospitalizations or ED visits (**Supplementary Table 1**), mental health crises (e.g., anxiety, depression, suicidality (48); **Supplementary Table 3**), all-drug overdoses (e.g., drugs, alcohol, nicotine; **Supplementary Table 4**), and relapse (a composite of SUD visit, overdose, or detoxification; **Supplementary Tables 1**, **4**, **5**). Incident rates (IRs) were calculated per 10,000 person-months.

### Exposures

Treatment exposures during baseline included outpatient SUD services (OS; **Supplementary Table 6**), general anesthetic prescriptions excluding ketamine (e.g., Propofol, Midazolam, Etomidate, Sevoflurane, Desflurane, **Supplementary Table 7**; from outpatient encounters not overlapping with any procedures, **Supplementary Table 8**) and psychedelic use (mainly ketamine; **Supplementary Table 2**). Psychedelics were classified based on outpatient procedures or prescriptions, with ketamine categorized solely under psychedelics. Exposures were analyzed as binary (yes/no), ordinal (number of treatments), and nominal (combinations of treatment types).

### Additional measures

Covariates and factors included demographic (e.g., age, sex, race/ethnicity, insurance, geography), clinical (e.g., Charlson Comorbidity Index (49), index SUD type, chronic pain), behavioral (e.g., tobacco use, social determinants coded as ICD-10 Z55-Z65), and treatment history (e.g., anesthetic, benzodiazepine, medications for substance use disorder (MSUD) prescriptions; **Supplementary Tables 1**, **9**-**13**) as determined by literature (32–34). Most were assessed any time before or during the baseline, with some restricted to specific periods.

### Statistical analysis

Descriptive statistics and standardized mean differences (SMDs) evaluated covariate balance between treatment groups. To address covariate imbalance, propensity scores were calculated via logistic (binary) and multinomial (ordinal/nominal) regressions using the average treatment effect (ATE) estimand. Propensity score weighted covariate balance was visualized in **Supplementary Figures 1A-L**, and doubly robust adjustment was applied for residual imbalances. Adjusted incidence rate ratios (aIRRs) were estimated using mixed-effects quasi-Poisson regression, incorporating person-time as an offset, clustering by hospital, and weighted with propensity score weights. Models were further adjusted for covariates to mitigate residual confounding. Quasi-Poisson was chosen based on overdispersion confirmed via Cameron and Trivedi’s test (50). Model fit was assessed via residual deviance relative to degrees of freedom.

### Sensitivity analyses

Supplemental analyses repeated all models among patients with a recent mental health diagnosis (within two years before or during baseline) and included adjustment for antidepressant use (e.g., selective serotonin reuptake inhibitors [SSRIs], serotonin-norepinephrine reuptake inhibitors [SNRIs], atypical antidepressants, tricyclic antidepressants, monoamine oxidase inhibitors [MAOIs]; **Supplementary Table 14**). A secondary analysis redefined OS to include MSUD, reflecting MAT. These were used to test treatment combinations involving MAT, anesthetics, and psychedelics. Due to warnings about combining sedating agents and MSUD medications (51–55), this analysis was exploratory. All tests were two-sided with α=0.05. Analyses were conducted using R version 4.0.2 (The R Foundation).

## Results

### Descriptive statistics

A total of 3,209,798 patients with SUD were analyzed. Of these, 0.3% (8,514) used psychedelic prescriptions, primarily ketamine, while 99.7% (3,201,284) did not. Baseline use of outpatient SUD services (OS) was observed in 3.7%, and general anesthetic prescriptions in 2.3%. Most patients were male (53.1%), non-Hispanic White (69.9%), and single (64.6%). Index diagnoses included 10% with OUD, 18.7% with AUD, 62.2% with TUD, and smaller proportions with cannabis (6%), stimulant (4.4%), or psychotropic medication (3.9%) use disorders; over 7% had multiple index SUDs (**Table 1**).

**Table 1:** Characteristics of patients diagnosed with SUD^1^, overall and by psychedelic use treatment^2^.

Total	Total	Psychedelics	No Psychedelics	SMD^5^
	n (%^3^)	n (%^3^)	n (%^3^)	
	3,209,798	8,514 (0.3^4^)	3,201,284 (99.7^4^)	
Age (Years)^6^	46.8 (17.2)	50.3 (14.6)	46.8 (17.2)	0.223
Sex				0.015
Female	1503953 (46.9)	4054 (47.6)	1499899 (46.9)	
Male	1705275 (53.1)	4458 (52.4)	1700817 (53.1)	
Race/Ethnicity				0.118
NH^7^-AI/AN^8^	45543 (1.4)	78 (0.9)	45465 (1.4)	
NH-Asian	21894 (0.7)	35 (0.4)	21859 (0.7)	
NH-Black	365299 (11.4)	833 (9.8)	364466 (11.4)	
Hispanic/Latino	427148 (13.3)	947 (11.1)	426201 (13.3)	
NH-NH/PI^9^	4477 (0.1)	12 (0.1)	4465 (0.1)	
NH-White	2243744 (69.9)	6374 (74.9)	2237370 (69.9)	
NH-Other	57885 (1.8)	133 (1.6)	57752 (1.8)	
Unknown	43808 (1.4)	102 (1.2)	43706 (1.4)	
Marital status				0.081
Married/Partner	972440 (30.3)	2720 (31.9)	969720 (30.3)	
Single	2074389 (64.6)	5230 (61.4)	2069159 (64.6)	
Unknown	162969 (5.1)	564 (6.6)	162405 (5.1)	
U.S. Census Division				0.436
New England	175158 (5.5)	361 (4.2)	174797 (5.5)	
Mid Atlantic	349658 (10.9)	684 (8.0)	348974 (10.9)	
South Atlantic	428861 (13.4)	1608 (18.9)	427253 (13.3)	
East North Central	365059 (11.4)	844 (9.9)	364215 (11.4)	
East South Central	493539 (15.4)	1163 (13.7)	492376 (15.4)	
West North Central	176989 (5.5)	778 (9.1)	176211 (5.5)	
West South Central	198039 (6.2)	1308 (15.4)	196731 (6.1)	
Mountain	757597 (23.6)	1334 (15.7)	756263 (23.6)	
Pacific	226454 (7.1)	338 (4.0)	226116 (7.1)	
Unknown	38444 (1.2)	96 (1.1)	38348 (1.2)	
Metropolitan				0.322
Metropolitan	2661041 (83.6)	5955 (70.3)	2655086 (83.7)	
Non-Metropolitan	520890 (16.4)	2519 (29.7)	518371 (16.3)	
Rural				0.292
Rural	538929 (16.9)	2462 (29.1)	536467 (16.9)	
Urban	2643001 (83.1)	6013 (70.9)	2636988 (83.1)	
Year of encounter				0.624
2000-2003	8940 (0.3)	0 (0.0)	8940 (0.3)	
2004-2007	46856 (1.5)	11 (0.1)	46845 (1.5)	
2008-2011	194620 (6.1)	102 (1.2)	194518 (6.1)	
2012-2015	665676 (20.7)	713 (8.4)	664963 (20.8)	
2016-2019	1230858 (38.3)	2697 (31.7)	1228161 (38.4)	
2020-2023	1062848 (33.1)	4991 (58.6)	1057857 (33.0)	
Insurance				0.199
Private	888521 (27.7)	2886 (33.9)	885635 (27.7)	
Medicare	764962 (23.8)	2211 (26.0)	762751 (23.8)	
Medicaid	801188 (25.0)	1760 (20.7)	799428 (25.0)	
Other govt/misc.	205274 (6.4)	581 (6.8)	204693 (6.4)	
Self-Pay	454874 (14.2)	944 (11.1)	453930 (14.2)	
Unknown	94979 (3.0)	132 (1.6)	94847 (3.0)	
CCI^10^ Categorized				0.043
0	2037937 (63.5)	5559 (65.3)	2032378 (63.5)	
1-2	558719 (17.4)	1460 (17.1)	557259 (17.4)	
3-4	344874 (10.7)	843 (9.9)	344031 (10.7)	
≥5	268268 (8.4)	652 (7.7)	267616 (8.4)	
Index SUDs^11^ (Yes)				
Opioids	322438 (10.0)	1082 (12.7)	321356 (10.0)	0.084
Alcohol	600996 (18.7)	938 (11.0)	600058 (18.7)	0.218
Tobacco	1997938 (62.2)	6250 (73.4)	1991688 (62.2)	0.241
Cannabis	193826 (6.0)	311 (3.7)	193515 (6.0)	0.112
Sedatives	32316 (1.0)	33 (0.4)	32283 (1.0)	0.075
Stimulants	139664 (4.4)	130 (1.5)	139534 (4.4)	0.168
Inhalants	1034 (0.0)	1 (0.0)	1033 (0.0)	0.014
Psychotropic medications	124768 (3.9)	200 (2.3)	124568 (3.9)	0.089
Other SUD	69341 (2.2)	61 (0.7)	69280 (2.2)	0.122
Number of index SUDs				0.092
1	2978186 (92.8)	8071 (94.8)	2970115 (92.8)	
2	197489 (6.2)	401 (4.7)	197088 (6.2)	
3	28340 (0.9)	35 (0.4)	28305 (0.9)	
≥4	5783 (0.2)	7 (0.1)	5776 (0.2)	
History of mental health conditions^11^ (Yes)				
Anxiety	621213 (19.4)	1888 (22.2)	619325 (19.3)	0.070
Depression	599674 (18.7)	1719 (20.2)	597955 (18.7)	0.038
ADD/ADHD^12^	65127 (2.0)	171 (2.0)	64956 (2.0)	0.001
Bipolar	201925 (6.3)	412 (4.8)	201513 (6.3)	0.064
Schizophrenia/Psychotic	71777 (2.2)	76 (0.9)	71701 (2.2)	0.109
PTSD^13^	79693 (2.5)	248 (2.9)	79445 (2.5)	0.027
Other	80691 (2.5)	195 (2.3)	80496 (2.5)	0.015
Number of mental health conditions				0.035
0	2153350 (67.1)	5653 (66.4)	2147697 (67.1)	
1	593623 (18.5)	1550 (18.2)	592073 (18.5)	
2	314514 (9.8)	924 (10.9)	313590 (9.8)	
≥3	148311 (4.6)	387 (4.5)	147924 (4.6)	
Chronic pain (Yes)	1611115 (50.2)	5553 (65.2)	1605562 (50.2)	0.309
Problems related to lifestyle (Yes)	491525 (15.3)	1327 (15.6)	490198 (15.3)	0.008
Adverse socioeconomic/psychosocial determinants of health (Yes)	72394 (2.3)	162 (1.9)	72232 (2.3)	0.025
MSUD^14, 15^ (Yes)	161560 (5.0)	662 (7.8)	160898 (5.0)	0.113
Outpatient SUD Services^15^ (Yes)	117651 (3.7)	226 (2.7)	117425 (3.7)	0.058
MAT^16, 15^ (Yes)	272993 (8.5)	862 (10.1)	272131 (8.5)	0.056
Anesthetics^11^ (Yes)				
History	204390 (6.4)	1268 (14.9)	203122 (6.3)	0.280
Baseline	73691 (2.3)	3137 (36.8)	70554 (2.2)	0.972
Anesthetic types at baseline^11^ (Yes)				
Propofol	51160 (1.6)	3004 (35.3)	48156 (1.5)	0.969
Methohexital	103 (0.0)	0 (0.0)	103 (0.0)	0.008
Thiopental	2 (0.0)	0 (0.0)	2 (0.0)	0.001
Isoflurane	78 (0.0)	2 (0.0)	76 (0.0)	0.019
Desflurane	495 (0.0)	31 (0.4)	464 (0.0)	0.080
Etomidate	537 (0.0)	23 (0.3)	514 (0.0)	0.067
Sevoflurane	2530 (0.1)	158 (1.9)	2372 (0.1)	0.183
Midazolam	49443 (1.5)	2614 (30.7)	46829 (1.5)	0.868
Other	3355 (0.1)	38 (0.4)	3317 (0.1)	0.065
Antidepressants^11, 17^ (Yes)				
History	415136 (43.5)	1251 (47.2)	413885 (43.4)	0.076
Baseline	284287 (29.8)	635 (24.0)	283652 (29.8)	0.131
Antidepressant types at baseline^11, 17^ (Yes)				
SSRIs^18^	169593 (17.8)	337 (12.7)	169256 (17.8)	0.141
SNRIs^19^	55162 (5.8)	157 (5.9)	55005 (5.8)	0.007
Atypical antidepressants	130171 (13.6)	275 (10.4)	129896 (13.6)	0.100
Tricyclic antidepressants	18436 (1.9)	78 (2.9)	18358 (1.9)	0.066
MAOIs^20^	153 (0.0)	1 (0.0)	152 (0.0)	0.013
Benzodiazepine prescription^15^ (Yes)	823651 (25.7)	7205 (84.6)	816446 (25.5)	1.478
Procedure history^21^ (Yes)	203358 (6.3)	3049 (35.8)	200309 (6.3)	0.778
SUD hospitalization/ED visit history (Yes)	1881976 (58.6)	2817 (33.1)	1879159 (58.7)	0.532
Mental health crises history^22^ (Yes)	256205 (8.0)	429 (5.0)	255776 (8.0)	0.120
All-drug overdose history^23^ (Yes)	287040 (8.9)	347 (4.1)	286693 (9.0)	0.199
Relapse history^24^ (Yes)	141080 (4.4)	303 (3.6)	140777 (4.4)	0.043

^1^ SUD diagnosis at inpatient/emergency encounter or >=2 SUD diagnoses within two years of each other from any encounter type; excluding those with any history (or baseline occurrence) of hallucinogen use disorder (HUD) or hallucinogen/psychodysleptic-related overdose, additionally excluding those with any psychedelic history (procedures, medications, or positive urine drug tests from any encounter), with index encounters from January 1, 2000 to August 31, 2023, having at least 3 months of data post index SUD (baseline treatment period).

^2^ outpatient procedure-based indication or prescription (at outpatient encounters) occurring in baseline period (up to three months after index SUD date).

^3^ column % (unless otherwise noted).

^4^ row % (out of total [n=3,209,798]).

^5^ standardized mean difference.

^6^ mean (standard deviation).

^7^ non-Hispanic.

^8^ American Indian or Alaskan Native.

^9^ Native Hawaiian or Other Pacific Islander.

^10^ Charlson comorbidity index.

^11^ not mutually exclusive.

^12^ attention-deficit disorder/attention-deficit/hyperactivity disorder.

^13^ post traumatic stress disorder.

^14^ medications for substance use disorder.

^15^ occurring in baseline treatment period (up to three months after index SUD).

^16^ medication assisted treatment: MSUD or outpatient SUD-related services.

^17^ %’s calculated further among those with recent mental health condition (diagnosis up to two years prior to index SUD and up to three months after [n=955,368]).

^18^ selective serotonin reuptake inhibitors.

^19^ serotonin-norepinephrine reuptake inhibitors.

^20^ monoamine oxidase inhibitors.

^21^ occurring up to one year prior to index SUD and within baseline period.

^22^ conditions of suicide (any encounter) or anxiety/depression (from emergency encounters).

^23^ any drug overdose, alcohol intoxication, or tobacco poisoning.

^24^ any SUD hospitalization/ED visit, all-drug overdose, or detoxification service occurring 30 days apart.

Mental health comorbidities were common, with 19.4% having anxiety and 18.7% depression; nearly 32% had at least one mental health diagnosis. Over half experienced chronic pain or prior SUD-related hospitalization/ED use. Compared to non-users, psychedelic users were older, more often lived in non-metropolitan or rural areas, had more recent index encounters, private insurance, chronic pain, benzodiazepine prescriptions, procedures, anesthetic treatment, and lower baseline adverse outcomes (**Table 1**).

During follow-up, incidence rates per 10,000 person-months were 199.71 for SUD hospitalization/ED visits, 138.12 for mental health crises, 49.62 for all-drug overdoses, and 62.21 for relapse (**Table 2**).

**Table 2:** Association between treatment and adverse healthcare outcomes (SUD-hospitalizations/ED visits, mental health crisis, all-drug overdose, relapse), among those with SUD.

	SUD hospitalization/ED visit		Mental health crisis		All-drug overdose		Relapse	
	n^1^ (IR^2^)	aIRR^3^ (95% CI^4^)	n^1^ (IR^2^)	aIRR^3^ (95% CI^4^)	n^1^ (IR^2^)	aIRR^3^ (95% CI^4^)	n^1^ (IR^2^)	aIRR^3^ (95% CI^4^)
Overall	1498433 (199.71)	–	1036281 (138.12)	–	372328 (49.62)	–	466777 (62.21)	–
Individual treatments								
OS^5^								
No	1481200 (205.32)	1 [REF]	1009645 (139.95)	1 [REF]	365253 (50.63)	1 [REF]	461986 (64.04)	1 [REF]
Yes	17233 (59.67)	**0.36 (0.35, 0.37)**	26636 (92.24)	**0.57 (0.56, 0.59)**	7075 (24.50)	**0.51 (0.48, 0.54)**	4791 (16.59)	**0.32 (0.31, 0.33)**
Anesthetics								
No	1467776 (200.46)	1 [REF]	1006136 (137.42)	1 [REF]	369346 (50.440)	1 [REF]	461986 (64.04)	1 [REF]
Yes	30657 (169.25)	**0.86 (0.84, 0.88)**	30145 (166.42)	**0.89 (0.87, 0.91)**	2982 (16.46)	**0.44 (0.40, 0.48)**	4791 (16.59)	**0.81 (0.78, 0.84)**
Psychedelics								
No	1495223 (199.78)	1 [REF]	1033615 (138.11)	1 [REF]	371801 (49.68)	1 [REF]	461986 (64.04)	1 [REF]
Yes	3210 (171.17)	**0.81 (0.76, 0.86)**	2666 (142.16)	**0.86 (0.79, 0.94)**	449 (23.94)	**0.46 (0.36, 0.60)**	4791 (16.59)	**0.76 (0.69, 0.84)**
Sum of treatments								
0	1449104 (206.14)	1 [REF]	979022 (139.27)	1 [REF]	362106 (51.51)	1 [REF]	452474 (64.37)	1 [REF]
1	47581 (103.83)	**0.56 (0.55, 0.57)**	55083 (120.2)	**0.70 (0.69, 0.71)**	9906 (21.62)	**0.47 (0.45, 0.50)**	13775 (30.06)	**0.52 (0.51, 0.53)**
2	1725 (116.31)	**0.53 (0.48, 0.58)**	2164 (145.91)	**0.66 (0.60, 0.73)**	309 (20.83)	**0.43 (0.34, 0.52)**	521 (35.13)	**0.49 (0.44, 0.56)**
3	23 (89.84)	**0.46 (0.21, 0.99)**	12 (46.87)	**0.21 (0.06, 0.75)**	7 (27.34)	0.61 (0.10, 3.81)	7 (27.34)	*0.46 (0.16, 1.09)*
Combinations of treatments								
None	1449104 (206.14)	1 [REF]	979022 (139.27)	1 [REF]	362106 (51.51)	1 [REF]	452474 (64.37)	1 [REF]
OS only	16640 (59.23)	**0.35 (0.34, 0.36)**	25609 (91.16)	**0.57 (0.56, 0.59)**	6886 (24.51)	**0.50 (0.47, 0.53)**	4612 (16.42)	**0.32 (0.30, 0.33)**
Anesthetics only	28934 (173.96)	**0.86 (0.84, 0.88)**	28003 (168.36)	**0.88 (0.86, 0.91)**	2677 (16.09)	**0.41 (0.37, 0.45)**	8610 (51.77)	**0.80 (0.77, 0.82)**
Psychedelics only	2007 (182.72)	**0.76 (0.69, 0.82)**	1471 (133.92)	**0.82 (0.73, 0.92)**	343 (31.23)	**0.48 (0.37, 0.63)**	553 (50.35)	**0.68 (0.60, 0.77)**
OS and anesthetics	545 (74.48)	**0.41 (0.35, 0.48)**	981 (134.06)	**0.57 (0.50, 0.66)**	171 (23.37)	**0.58 (0.40, 0.84)**	166 (22.69)	**0.39 (0.31, 0.49)**
OS and psychedelics	25 (90.20)	**0.33 (0.16, 0.70)**	34 (122.68)	0.56 (0.26, 1.20)	11 (39.69)	0.48 (0.11, 2.08)	6 (21.65)	**0.26 (0.08, 0.81)**
Anesthetics and psychedelics	1155 (159.61)	**0.79 (0.71, 0.88)**	1149 (158.78)	**0.81 (0.71, 0.93)**	127 (17.55)	**0.39 (0.25, 0.60)**	349 (48.23)	**0.76 (0.65, 0.88)**
OS, anesthetics, and psychedelics	23 (89.84)	**0.46 (0.20, 0.98)**	12 (46.87)	**0.21 (0.06, 0.77)**	7 (27.34)	0.60 (0.10, 3.78)	7 (27.34)	*0.47 (0.16, 1.10)*

^1^ count of events occurring within >=3 months and <=27 months after index SUD (allowing a two-year follow-up period).

^2^ incidence rate (per 10,000 person-months).

^3^ adjusted incidence rate ratio, via Quasipoisson regression; sample balanced on treatment status (separately for each individual, summation, and combination) via logistic/multinomial regression propensity score weighting with average treatment effect in population (ATE) estimand, by age, gender, race/ethnicity, marital status, census division, metropolitan status, rural status, year of index SUD, insurance, comorbidity, index SUD count, number of mental health conditions history, chronic pain history, problems related to lifestyle history, adverse socioeconomic/psychosocial determinants of health history, anesthetics history, benzodiazepine prescription, procedure history, MSUD, SUD hospitalization history, mental health crisis history, all-drug overdose history, relapse history (and OS, anesthetics at baseline, psychedelics at baseline with respective removal given the treatment chosen of interest for balancing), doubly adjusted for residual imbalance. Bolded values signify statistically significant results: p<0.05. Grayed/italicized values signify results on the boundary of significance: 0.05≤p<0.1.

^4^ confidence interval.

^5^ outpatient SUD services.

### Inferential statistics

After propensity score weighting, treatment groups showed lower rates of adverse outcomes. OS use was associated with the lowest rates of SUD hospitalization/ED visit (aIRR: 0.36; 95% confidence interval [CI]: 0.35-0.37), mental health crisis (aIRR: 0.57; 95% CI: 0.56-0.59), and relapse (aIRR: 0.32; 95% CI: 0.31-0.33) compared to those without OS. General anesthetics (aIRR: 0.44; 95% CI: 0.40-0.48) and psychedelics (aIRR: 0.46; 95% CI: 0.36-0.60) were associated with the lowest overdose rates (**Table 2**).

Patients receiving one or two treatments had lower rates of all outcomes compared to untreated patients. Those receiving all three treatments had a 54% lower rate of SUD hospitalization/ED visits (aIRR: 0.46; 95% CI: 0.21-0.99), 79% lower rate of mental health crises (aIRR: 0.21; 95% CI: 0.06-0.75), and 54% lower relapse (aIRR: 0.46; 95% CI: 0.16-1.09).

By treatment combinations, patients receiving only OS had significantly lower rates of all outcomes compared to those with no treatment: SUD hospitalization/ED visit (aIRR: 0.35; 95% CI: 0.34-0.36), mental health crisis (aIRR: 0.57; 95% CI: 0.56-0.59), overdose (aIRR: 0.50; 95% CI: 0.47-0.53), and relapse (aIRR: 0.32; 95% CI: 0.30-0.33). Anesthetic-only users had a 59% lower overdose rate (aIRR: 0.41; 95% CI: 0.37-0.45) and modest reductions across other outcomes. Psychedelic-only users had a 52% lower overdose rate (aIRR: 0.48; 95% CI: 0.37-0.63), along with reductions in hospitalization (aIRR: 0.76; 95% CI: 0.69-0.82), mental health crises (aIRR: 0.82; 95% CI: 0.73-0.92), and relapse (aIRR: 0.68; 95% CI: 0.60-0.77).

Patients receiving OS and anesthetics had significantly lower rates of all outcomes, including SUD hospitalization/ED visit (aIRR: 0.41; 95% CI: 0.35-0.48), mental health crisis (aIRR: 0.57; 95% CI: 0.50-0.66), overdose (aIRR: 0.58; 95% CI: 0.40-0.84), and relapse (aIRR: 0.39; 95% CI: 0.31-0.49). Those with OS and psychedelics experienced the lowest relapse rate (aIRR: 0.26; 95% CI: 0.08-0.81) and a 67% lower SUD hospitalization rate (aIRR: 0.33; 95% CI: 0.16-0.70). Anesthetics and psychedelics were associated with lower rates of all outcomes, including a 61% reduction in overdose (aIRR: 0.39; 95% CI: 0.25-0.60).

Patients receiving all three treatments had a 54% lower rate of SUD hospitalization/ED visits (aIRR: 0.46; 95% CI: 0.20-0.98), a 53% lower relapse rate (aIRR: 0.47; 95% CI: 0.16-1.10), and a 79% lower mental health crisis rate (aIRR: 0.21; 95% CI: 0.06-0.77), though the association with overdose was not significant.

### Supplemental analyses

In the subset with recent mental health conditions (n = 955,368; **Supplementary Table 15**), incidence rates were higher across all outcomes. Treatment associations remained directionally consistent, though statistical significance varied due to smaller subgroup sizes. Notably, trends for psychedelics, anesthetics, and OS persisted.

When redefining OS to include MSUD as MAT (**Supplementary Table 16**), results were similar. Patients with MAT had lower rates of SUD hospitalization (aIRR: 0.72; 95% CI: 0.71-0.73), mental health crises (aIRR: 0.74; 95% CI: 0.73-0.75), overdose (aIRR: 0.70; 95% CI: 0.68-0.73), and relapse (aIRR: 0.70; 95% CI: 0.69-0.71), though effects were slightly weaker than with OS alone. MAT in combination with anesthetics or psychedelics also showed protective trends, with the three-treatment model yielding reduced outcomes, though some estimates were non-significant.

## Discussion

Among a large sample of patients with SUD, this study identified protective associations between outpatient SUD-related encounters, anesthetics, and psychedelics and adverse healthcare outcomes. These associations were found when treatments were used alone and were often stronger when used in combination. Several remained significant when restricted to those with recent mental health conditions. This study offers one of the first real-world, large-scale estimates of psychedelic and anesthetic use, primarily ketamine, in structured clinical settings, highlighting their potential integration within existing care systems. This study provides the first estimates of adverse outcomes associated with anesthetics and psychedelics used alongside outpatient care in a large, diverse SUD cohort.

Crucially, most published evidence on psychedelics stems from small trials or non-clinical surveys of naturalistic use. In contrast, this study utilizes clinical EHR data to assess psychedelic use in health system settings, making it one of the few pragmatic evaluations of these therapies as they are currently implemented. This distinction is essential, as real-world outcomes are influenced not only by pharmacology but also by clinical supervision, patient selection, and healthcare context.

Psychedelic use was rare, with only 0.3% of patients having a qualifying exposure. Several factors likely explain this. First, psychedelic exposure was limited to outpatient prescriptions or procedures among patients without prior use. Most qualifying indications involved ketamine, which remains uncommon (292 per million Medicaid enrollees in 2020) (56). Psychedelic treatments are often reserved for patients unresponsive to conventional therapy and were only recently included in clinical practice for SUD or mental health (15). Our dataset, which captures structured care, may underreport usage that occurs in informal settings. In a survey of over 2,000 Canadian patients, 33.7% reported using psychedelics to self-treat a health condition, but only 4.4% did so with a therapist and 3.6% in clinical settings (57). Thus, true prevalence may be higher. Second, although research highlights benefits of psychedelics in treating substance use (24, 27–30, 35, 36), adverse effects are still possible (21, 22, 58). In a survey of over 300 U.S. psychologists, many expressed cautious interest, citing psychiatric risks (59). Finally, there was also a large overlap between psychedelic use and anesthetic use found in this patient sample and is likely explained by the compound ketamine which has known psychedelic properties and is more often used as a form of anesthesia (60).

This study assessed associations between three treatment categories, OS, anesthetics, and psychedelics, and four adverse outcomes: SUD-related hospitalizations or ED visits, mental health crises, all-drug overdose, and relapse. Across outcomes, each treatment was associated with significantly lower rates relative to no treatment. These effects were also observed among those who received only one treatment. OS was most protective for hospitalizations, mental health crises, and relapse. This aligns with research showing that outpatient treatment improves abstinence, reduces use, and decreases rehospitalization (61–64). In contrast, the results indicate that anesthetics and psychedelics were most strongly protective of overdose compared to OS treatment. The capacity of anesthetic drugs to impact substance use outcomes has not been thoroughly explored in research likely due to the addictive potential of non-opioid anesthetics (65). However, a recent animal study did conclude that isoflurane had a strong inhibitory effect on cocaine-reinforced behavior in rats (66). The protective association found between anesthetics and all-drug overdose is a novel discovery that should be further explored in future research. Ample research has shown evidence of psychedelic compounds reducing substance use tendencies. Multiple surveys have revealed individuals reporting reductions in, alcohol, cannabis, and opioid cravings following naturalistic psychedelic use (67–69). Congruent with these findings, psychedelic use among the patient sample likely resulted in a substance use reduction that was illustrated by the strong protective association for all-drug overdose.

Patients receiving multiple treatments experienced lower rates of all outcomes compared to those without any treatment. This supports recommendations to combine pharmacotherapy and behavioral therapy for optimal outcomes (70, 71). The combined use of all three treatments (OS, anesthetics, and psychedelics) was significantly protective against hospitalization and mental health crises. Notably, the greatest protective association in the primary analysis, 79% reduction, was observed between all three treatments and mental health crises. Anesthetics and ketamine are used to treat mental illness (31, 39, 72), and outpatient care is known to reduce mental health symptom severity (61, 73). Therefore, the strongest mental health protection seen with all three treatments is plausible. Additionally, three treatment strategies did result in a protective association with relapse being on the boundary of significance but all-drug overdose being insignificant. The sample subset of patients that used a sum of three treatment strategies was very small, which likely influenced the statistical power of the analysis explaining the lack of statistical significance seen in this group. The consistent protective effect, though insignificant, is still notable and warrants further efforts to study the impacts of these treatments used together.

We also examined outcomes by explicit treatment combinations. Those receiving both OS and anesthetics or both anesthetics and psychedelics had significantly lower rates across all outcomes. These combinations have precedent in procedural and psychiatric contexts (39, 74, 75). For example, ketamine combined with anesthetics is widely used in procedural sedation and may confer mental health benefits. Our findings suggest these pairings could improve outcomes in SUD as well. The OS and psychedelic group showed the lowest relapse and hospitalization rates in the primary analysis. This supports prior findings that psychological interventions enhance psychedelic treatment. A trial combining psilocybin with motivational enhancement therapy reported reduced alcohol cravings and increased abstinence (76). A review of ketamine for SUD suggested its effects may be amplified when combined with cognitive-behavioral therapy (77). While the combination showed protective trends for mental health crises and overdose, results were not statistically significant, likely due to small sample size. Still, these findings support further evaluation of integrated psychedelic therapies for SUD.

In the subset of patients with a recent mental health condition, trends were consistent, though statistical significance was attenuated in several comparisons due to reduced sample size. Incidence rates were higher across all outcomes, consistent with literature showing that SUD with psychiatric comorbidity results in worse health (78–83). Despite this, treatment effects remained directionally protective, suggesting these approaches may benefit complex patients.

In the second supplemental analysis, MAT was defined as a combination of MSUD and OS. While all treatments showed protective trends, OS alone was more protective than MAT across all outcomes. This may reflect differences in illness severity, with MAT patients representing more chronic or complex cases. Alternatively, lower adherence or greater side effect burden could reduce effectiveness. Despite this, MAT still showed significant protective effects, consistent with its known benefits in reducing withdrawal symptoms and substance use (84, 85).

When MAT was used in combination with anesthetics or psychedelics, results remained protective for most outcomes. Some associations did not reach statistical significance, which could reflect lower power or pharmacological interactions. While combining sedatives with medications for opioid, alcohol, or tobacco use disorders carries known risks (51–55), patients may require complex regimens. Encouragingly, these patients still experienced reduced adverse outcomes relative to those without treatment, suggesting benefits may outweigh risks in appropriate cases.

Taken together, these findings underscore the clinical relevance of ketamine as a pragmatic psychedelic treatment option within U.S. healthcare systems. By leveraging structured clinical data rather than relying solely on self-report or experimental trials, this study contributes meaningfully to the real-world evidence base guiding future SUD treatment innovation. While outpatient therapy remains a critical foundation, adjunctive treatments, particularly ketamine and other anesthetics, could further reduce relapse, overdose, and mental health burden. Given the novelty of psychedelic therapies and the complexity of their use alongside other medications, additional research is warranted to determine optimal timing, combinations, and patient selection.

### Limitations

There are several limitations of this study design to consider when interpreting the results of these analyses. For one, the retrospective study design only produces correlational estimates between SUD treatment methods and adverse healthcare outcomes limiting the ability to assume causality of these variables. Furthermore, the data examined in this study is limited to health indications within OERWD-affiliated centers and may not be completely generalizable to health centers outside of this specific database. Additionally, it is important to note that the psychedelic treatment cohort in our analyses is largely made up of prescription ketamine indications. The true number of psychedelic users (primarily self-medicating) is likely larger than the number that could be captured with the OERWD. Moreover, the analysis lacked details on dosing, frequency, administration route, and treatment setting, which limited the ability to explore potential dose-response effects or distinguish structured therapeutic use from general medical administration. In reference to SUD treatment combinations, patients using a combination of treatment methods had an indication of a particular treatment method within the span of three months as opposed to explicit simultaneous indications. This methodology could have potentially included patients that were switching from one treatment to another rather than intentionally using a combination of treatment strategies. Further, specific use contexts (e.g., the combination of ketamine and anesthetics being used specifically for SUD treatment) was not explicitly captured in this study; rather analyses used longitudinal context (i.e., identifying patients with SUD that had following indications of both psychedelics and anesthetics within a three-month period) to determine treatment assignments. Finally, although extensive propensity score weighting was used to reduce bias, the possibility of unmeasured confounding remains. For example, providers’ prescribing behavior, patient motivation, treatment adherence, and socioeconomic context may all influence treatment selection and outcomes but were not fully considered. Despite the given limitations, our study’s results fill a gap in the current literature of the potential impacts of psychedelic compounds on SUD treatment-related healthcare outcomes when used with and without traditional treatment strategies. Most importantly, these results stem from an entire patient population of overall SUD from a large, diverse, nationwide sample.

## Conclusion

In a diverse national cohort of patients with SUD, psychedelic, anesthetic, and outpatient treatment, alone and in combination, were associated with significantly reduced risks of relapse, overdose, psychiatric crisis, and hospitalization. These findings support the growing investigation of psychedelic-based and anesthetic-supported interventions as part of an integrated treatment model for SUD. Further research should examine treatment timing, setting, and interactions with standard therapies to optimize outcomes and safety.

## Data Availability

The datasets generated during and/or analyzed during the current study are not publicly available because of restrictions by Oracle Cerner, the owner of the data. Data could be accessed by signing a data sharing agreement with Oracle Cerner and covering any costs that may be involved (contact Kendra Stillwell: kendra.stillwell@cernerenviza.com). Further inquiries can be directed to the corresponding author/s.
