# Supplementary material for: Associations between psychedelic use and adverse outcomes in substance use disorders: a real-world EHR-based cohort study
Source: Front Psychiatry. 2025 Oct 24;16:1648104. doi: 10.3389/fpsyt.2025.1648104 (PMC12592884; doi:10.3389/fpsyt.2025.1648104)
Supplement: Supplementary file 1 [file Supplementaryfile1.docx]

**Supplemental Table 1.**List of codes used to define substance use disorder by substance class

| **Substance Class** | **Code Type** | **Code(s)** |
| --- | --- | --- |
| Alcohol | ICD-9 | 291.X, 303.X, 305.00, 305.01, 305.02, 305.03, 357.5, 425.5, 535.30, 535.31, 571.0, 571.1, 571.2, 571.3, 760.71, E860.0, 980.X, E860.0 |
|  | ICD-9 Procedure | 94.46, 94.53, 94.61, 94.62, 94.63, 94.67, 94.68, 94.69 |
|  | SNOMED | 235875008, 1082611000119101, 307757001, 191802004, 191806001, 9953008, 713370005, 183486001, 15167005, 73097000, 66590003, 213687005, 212820004, 212819005, 212812001, 212812001, 212808007, 212807002, 212806006, 67426006, 82782008, 212809004, 10741871000119101, 7052005, 25702006, 18653004, 8635005, 191476005, 191480000, 34938008, 300992002, 235952002, 53936005, 29212009, 237738005, 42344001, 78524005, 41083005, 70701004, 192811002, 235875008, 191478006, 7200002, 191475009, 307757001, 713181003, 154221000119101, 154211000119108, 191804003, 191811004, 133301000119102, 281004, 191805002, 21000000, 445507008, 135311000119100, 55571001, 85561006, 713862009, 228281002, 284591009, 191477001, 191883007, 191882002, 268645007, 714829008, 713583005, 191471000 |
|  | ICD-10 | F10.X, G62.1, I42.6, K29.20, K29.21, K70.0, K70.10, K70.11, K70.2, K70.3, K70.30, K7.31, K70.40, K70.41, K70.9, T51.X, Z71.41 |
| Tobacco | ICD-9 | 305.1, 649.0X, 989.84 |
|  | SNOMED | 230064005, 160606002, 191888003, 191887008, 89765005, 449868002, 56294008, 56578002, 230062009, 160604004, 428071000124103, 56771006, 230063004, 455431000124101, 455421000124104, 455441000124106, 65568007, 191888003, 191887008, 212899006, 111779009, 291284006, 291285007, 291286008, 66884007 |
|  | CPT | 99406, 99407 |
|  | HCPCS | G9458 |
|  | ICD-10 | F17.X, O99.33X, T65.2X, T65.211A, T65.212A, T65.213A, T65.214A, T65.221A, T65.222A, T65.223A, T65.224A, T65.291A, T65.292A, T65.293A, T65.294A, Z72.0 |
| Cannabis | ICD-9 | 304.3X, 305.2X |
|  | SNOMED | 15233006, 216551003, 16292008, 291246000, 291247009, 291248004, 212666007, 1149328002, 85005007, 191838006, 191837001, 191839003, 11048011000119103 |
|  | ICD-10 | F12.X, T40.7X |
| Opioids | ICD-9 | 304.0X, 304.7X, 305.5X, 965.0, 965.00, 965.01, 965.02, 965.09, E850.0, E850.1, E850.2, E935.0, E935.1, E935.2 |
|  | SNOMED | 295213004, 297199006, 242253008, 295165009, 461001000124107, 288861000119108, 1081000119105, 724653003, 432353006, 426001001, 295195001, 295194002, 295193008, 295193008, 295186009, 295185008, 295184007, 295176008, 295175007, 295174006, 295172005, 295171003, 295170002, 295161000, 295148000, 292064003, 292063009, 292060007, 292059002, 292055008, 292052006, 290204003, 290203009, 290202004, 290201006, 290193005, 290183003, 290182008, 290181001, 290179003, 290172007, 290171000, 290170004, 290157008, 269264002, 242829007, 241749009, 231480002, 231479000, 231478008, 231477003, 222059008, 216470005, 216469009, 216468001, 216466002, 216465003, 216464004, 216463005, 213660006, 213659001, 213658009, 212676005, 212591003, 212588003, 212587008, 191914006, 191913000, 191912005, 191909007, 191820008, 191819002, 191817000, 75544000, 74264003, 68099003, 60199004, 18052008, 13187008, 11196001, 5602001, 191868002, 191867007, 191869005, 191865004 |
|  | ICD-10 | F11.X, R78.1, T40.0X, T40.1X, T40.2X, T40.3X, T40.4X, T40.6X |
| Sedatives | ICD-9 | 304.1X, 305.4X, E950.1, 967.0, E980.1 |
|  | SNOMED | 216497003, 216504000, 81914009, 216530001, 216537003, 212662009, 427327003, 64386003, 125851000119106 |
|  | ICD-10 | F13.X, T42.3X, T42.4X |
| Stimulants | ICD-9 | 304.2X, 304.4X, 305.6X, 305.7 |
|  | SNOMED | 216583009, 241761001, 291261004, 291263001, 291264007, 9982009, 290543000, 290544006, 290545007, 241760000, 216558009, 216561005, 442406005, 441527004 |
|  | ICD-10 | F14.X, F15.X, T40.5X, T43.6X |
| Hallucinogens/ psychodysleptic | ICD-9 | 304.5X, 305.3X, 969.6, E854.1 |
|  | SNOMED | 67893003, 216552005, 216550002, 213661005, 221809003, 221810008, 221811007, 221812000, 221813005, 221814004, 221815003, 221816002,221817006, 221818001, 221819009, 222070001, 222071002, 222072009, 222073004, 222074005, 222075006, 222076007, 222077003, 222078008, 222079000, 222080002, 222668009, 222669001, 222670000, 222671001, 222672008, 222673003,222674009, 222675005, 222676006, 222677002, 222678007, 38247002, 191850000, 191849000, 191851001, 74851005 |
|  | ICD-10 | F16.X, T40.8X, T40.9X |
| Inhalants | SNOMED | 213705007, 212900001, 216662006, 216663001, 216666009, 157833001, 5002000, 86401000119104, 86391000119101, 70340006, 427229002, 426095000 |
|  | ICD-10 | F18.X, T65.6X |
| Other SUD | ICD-9 | 292.X, 304.6X, 304.8X, 304.9X, 305.9X, V654.2 |
|  | ICD-9 Procedure | 94.45, 94.54, 94.64, 94.65, 94.66, 94.67, 94.68, 94.69 |
|  | SNOMED | 11387009, 396344000, 28368009, 39003006, 50026000, 83168008, 11061003, 2403008, 91388009, 724730008 |
|  | HCPCS | H0005, H0006, H0007, H0008, H0009, H0010, H0011, H0012, H0013, H0014, H0015, H0016, H0050, H0047, H2034, T1006 |
|  | ICD-10 | F19.X |
|  | ICD-10 Procedure | HZ2ZZZZ,  HZ30ZZZ, HZ31ZZZ, HZ32ZZZ, HZ33ZZZ, HZ34ZZZ, HZ35ZZZ, HZ36ZZZ, HZ37ZZZ, HZ38ZZZ, HZ39ZZZ, HZ3BZZZ, [HZ3CZZZ](https://www.icd10data.com/ICD10PCS/Codes/H/Z/3/C/HZ3CZZZ)  HZ40ZZZ, HZ41ZZZ, HZ42ZZZ, HZ43ZZZ, HZ44ZZZ, HZ45ZZZ, HZ46ZZZ, HZ47ZZZ, HZ48ZZZ, HZ49ZZZ, HZ4BZZZ, [HZ4CZZZ](https://www.icd10data.com/ICD10PCS/Codes/H/Z/3/C/HZ3CZZZ),  HZ50ZZZ, HZ51ZZZ, HZ52ZZZ, HZ53ZZZ, HZ54ZZZ, HZ55ZZZ, HZ56ZZZ, HZ57ZZZ, HZ58ZZZ, HZ59ZZZ, HZ5BZZZ, [HZ5CZZZ](https://www.icd10data.com/ICD10PCS/Codes/H/Z/3/C/HZ3CZZZ), HZ5DZZZ,  [HZ63ZZZ](https://www.icd10data.com/ICD10PCS/Codes/H/Z/6/3/HZ63ZZZ) |
| Psychotropic | SNOMED | 212653007, 219311001, 61438005, 699011008, 221786001, 212672007, 212673002, 213668004, 216544007, 216566000, 221787005, 221788000, 221789008, 221790004, 221791000, 221792007, 221793002, 221794008, 221795009, 221796005, 222048004, 222049007, 222050007, 222051006, 222052004, 222053009, 222054003, 222055002, 222056001, 222057005, 222058000, 222646004, 222647008, 222648003, 222649006, 222650006, 222651005, 222652003, 222653008, 222654002, 222655001, 222656000, 82225006 |
|  | ICD-10 | T43.8X, T43.9X |

**Supplemental Table 2.**List of codes used to define psychedelic use^1^

| **Code Type** | **Code(s)** |
| --- | --- |
| SNOMED | 15698006, 333847008, 333853008, 373464007, 3983008, 51251000112106, 5729000, 67757005, 781952002, 781953007, 105219000* |
| CPT | 0820T, 0821T, 0822T, 80357*, 80359*, 83992* |
| NDC | 00143950801, 00143950810, 00143950910, 00409205105, 00409205310, 38779175405, 38779175407, 38779175408 42023011310, 42023011410, 42023011510, 42023013710, 42023013810, 42852030370 50458002800, 50458002802, 50458002803, 55150043801, 55150043810, 55150043910, 5811002010C530,  61553033165, 61553050028, 61570058110, 67457000110, 67457018100, 67457018120, 69374030805, 69374051450, 69374098233, 69374098255, 70092111944, 70092112043, 70092156343, 70092912043, 70092956343, 70400020102005, 70400020102015, 7040002010E509, 7040002011E525,  7040002011E531, 71286302201, 71384070021, 71449006811, 71506005056,  72572032110, 73177010106, 96625003392900 |
| MMSL Synonym | 12190, 147885, 147886, 184393, 184403, 187713, 189831, 200480, 200494,  203184, 2119389, 2119391, 2130, 213318, 21961, 21962, 22371, 22598, 22599,  23057, 234048, 238082, 238083, 238084, 284333, 284334, 307297, 309865, 309966,  309967, 318372, 326992, 335770, 335772, 359493, 359494, 359495, 359496, 363250,  367294, 379456, 42523, 4497, 6130, 9883, 13487* |
| MMSL Drug ID | d00272, d08827, d09174 |
| LOINC* | 11052-8, 12327-3, 14267-9, 14310-7, 16254-5, 17033-2, 18322-8, 18358-2, 18392-1, 19499-3, 19568-5, 19569-3, 19570-1, 19571-9, 19659-2, 19660-0, 19661-8, 20537-7, 32107-5, 3732-5, 3934-7, 3935-4, 3936-2, 3937-0, 42922-5, 44424-0, 72799-0, 72825-3, 73971-4, 74653-7, 8235-4, 8236-2, 8238-8 |

**1** primary exposure of psychedelic use only involved codes from outpatient encounters (non-inclusive of LOINC codes or any other codes identified by “*”), all codes utilized for psychedelic use history

**Supplemental Table 3.**List of codes used to define mental health crisis

| **Crisis Type** | **Code Type** | **Code(s)** |
| --- | --- | --- |
| Suicide^1^ | SNOMED | 219118000, 219121003, 219107000, 219108005, 219117005, 219119008, 219124006, 219134002, 219135001, 219130006, 219120002, 219125007, 219126008, 219127004, 219128009, 219129001, 219132003, 219171000, 269726003, 269725004, 219136000, 219139007, 219140009, 219149005, 219154001, 219141008, 219142001, 219147007, 219150005, 219156004, 219157008, 219158003, 219159006, 219160001, 219161002, 219162009, 219163004, 219164005, 219171000, 219172007, 219173002, 219175009, 370908002, 219106009, 461291000124108, 461141000124108, 219137009, 269728002, 461181000124102, 461261000124100, 460981000124108, 460991000124106, 461001000124107, 461011000124105, 461021000124102, 461031000124104, 461041000124109, 461051000124106, 461081000124103, 461091000124100, 461111000124109, 461131000124103, 461151000124105, 461161000124107, 461201000124101, 461211000124103, 461271000124107, 269726003, 269808005, 287181000, 287182007, 287183002, 287184008, 287185009, 287186005, 287187001, 288311002, 36153001, 440144004, 53846008, 55554002, 82313006, 158062004, 158065002, 158066001, 158067005, 158068000, 158069008, 158070009, 158071008, 158072001, 158073006, 248061004, 222160001, 222161002, 222162009, 222163004, 222164005, 222165006, 222166007, 222167003, 222168008, 222169000, 222170004, 222171000, 222172007, 222173002, 222174008, 222175009, 222176005, 222177001, 222178006, 222179003, 222180000, 222181001, 222182008, 222183003, 222184009, 222185005, 222186006, 222187002, 222188007, 222189004, 222190008, 222191007, 222192000, 222193005, 222194004, 222195003, 222196002, 222197006, 222198001, 222199009, 222200007, 222201006, 222202004, 222203009, 222204003, 222205002, 222206001, 222207005, 222208000, 222209008, 222210003, 222212006, 222213001, 222214007, 222215008, 222216009, 222217000, 222218005, 222219002, 222220008, 222221007, 222222000, 222223005, 222224004, 222225003, 222226002, 222227006, 222228001, 222229009, 222230004, 222231000, 222232007, 222233002, 222234008, 222235009, 222236005, 222237001, 222238006, 222239003, 222240001, 222241002, 222242009, 222243004, 222244005, 222245006, 222246007, 222247003, 222248008, 222249000, 222250000, 222251001, 222252008, 222253003, 222254009, 222255005, 222256006, 222257002, 222258007, 222259004, 222260009, 222261008, 222262001, 222263006, 222264000, 222265004, 222266003, 222267007, 222268002, 222269005, 222270006, 222271005, 222272003, 222273008, 222274002, 222275001, 222276000, 222277009, 222278004, 222279007, 222280005, 222281009, 222282002, 222283007, 222284001, 222285000, 222286004, 222288003, 222289006, 222290002, 222291003, 222292005, 222293000, 222294006, 222295007, 222296008, 222297004, 222298009, 222299001, 222300009, 222301008, 222302001, 222303006, 222304000, 222306003, 222307007, 222308002, 222309005, 222310000, 222311001, 222312008, 222314009, 222315005, 222316006, 222317002, 222318007, 222319004, 222320005, 222321009, 222322002, 222323007, 222324001, 222325000, 222326004, 222327008, 222328003, 223318002, 223319005, 269743000, 418420002 |
|  | ICD-9 | E950.X, E951.X, E952.X, E953.X, E954, E955.X, E956, E957.X, E958.X, E959 |
|  | ICD-10 | T14.91, X71.X – X83.X |
| Anxiety Disorders^2^ | ICD-9 | 293.84, 300.10, 300.20, 300.21, 300.22, 300.3, 300.0X, 308.X |
|  | SNOMED | 48694002, 197480006, 21897009, 192404005, 21897009, 52910006, 160332003, 154882009, 268752000, 191706008, 192401002, 192459009, 191720001, 111487009, 16265701000119107, 17496003, 192398000, 192400001, 192405006, 197480006, 154884005, 191703000, 192192006, 65673007, 126943008, 192393009, 192397005, 192399008, 192403004, 268714001, 436001000124105, 69479009, 371631005, 386810004, 25501002, 52039009, 191736004, 71478004, 1376001, 191739006, 192411009, 192406007, 192410005, 192394003, 414371008, 111490003, 111491004, 11941006, 1380006, 1816003, 191722009, 191723004, 19766004, 22230001, 24781009, 30059008, 3158007, 31781004, 32388005, 34116005, 35607004, 38328002, 43150009, 4932002, 49564006, 50983008, 53956006, 5509004, 56576003, 59923000, 61212007, 61569007, 63701002, 63909006, 64060000, 65064003, 70691001, 72861004, 74010007, 76812003, 76868007, 8185002, 82415003, 82494000, 82738004, 83631006, 87798009, 89948007, 154885006 |
|  | ICD-10 | F06.4, F40.0X, F41.X, F42.X |
| Depression^2^ | ICD-9 | 296.92, 300.4, 309.1, 311, 296.2X, 296.3X, 296.5X |
|  | SNOMED | 25922000, 35489007, 320751009, 36923009, 370143000, 42925002, 69392006, 63778009, 87512008, 79298009, 48589009, 832007, 15639000, 73867007, 75084000, 430852001, 77911002, 20250007, 76441001, 19527009, 14183003, 300706003, 231499006, 321717001, 63412003, 30605009, 42810003, 70747007, 726772006, 320751009, 36923009, 370143000, 10811121000119102, 10811161000119107, 42925002, 69392006, 63778009, 87512008, 79298009, 16265951000119109, 720455008, 720454007, 832007, 15639000, 16266831000119100, 719592004, 720453001, 450714000, 73867007, 33736005, 60099002, 75084000, 251000119105, 430852001, 77911002, 20250007, 76441001, 16266991000119108, 19527009, 191606003, 191601008, 191602001, 191604000, 2506003, 19694002, 83176005, 38451003, 67711008, 85080004, 3109008, 36170009, 78667006, 2506003, 19694002, 83176005, 38451003, 67711008, 85080004, 3109008, 36170009, 191659001, 192080009, 35489007, 40379007, 191610000, 40379007, 191610000, 18818009, 191611001, 18818009, 191611001, 36474008, 281000119103, 36474008, 39809009, 191613003, 28475009, 33078009, 15193003, 39809009, 191613003, 28475009, 33078009, 15193003, 68019004, 33135002, 46244001, 191615005, 40568001, 38694004, 274948002, 38694004, 2618002, 191616006, 66344007, 319768000, 71336009, 268621008, 2618002, 720451004, 720452006, 66344007, 319768000, 71336009, 268621008 |
|  | ICD-10 | F31.4, F31.5, F31.75, F31.76, F31.77, F31.78, F31.81, F32.0, F32.1, F32.2, F32.3, F32.4, F32.5, F32.9, F32.A, F33, F34.1, F31.3X, F31.6X, F33.X |

**1** from any encounter type

**2** from inpatient or emergency encounters

**Supplemental Table 4.**List of codes used to define all-drug overdose

| **Overdose Type** | **Code Type** | **Code(s)** |
| --- | --- | --- |
| All-drug | SNOMED | 213647000, 213651003, 221742006, 221743001, 221744007, 221745008, 221746009, 221747000, 221748005, 221749002, 221750002, 221751003, 221752005, 222002008, 222003003, 222004009, 222005005, 222006006, 222008007, 222009004, 222010009, 222011008, 222012001, 222013006, 222601000, 222602007, 222603002, 222604008, 222605009, 222606005, 222607001, 222608006, 222609003, 222610008, 222611007, 216550002, 213661005, 221809003, 221810008, 221811007, 221812000, 221813005, 221814004, 221815003, 221816002, 221817006, 221818001, 221819009, 222070001, 222071002, 222072009, 222073004, 222074005, 222075006, 222076007, 222077003, 222078008, 222079000, 222080002, 222668009, 222669001, 222670000, 222671001, 222672008, 222673003, 222674009, 222675005, 222676006, 222677002, 222678007, 216579009, 213662003, 216587005, 11196001, 13187008, 18052008, 212587008, 212588003, 212591003, 212676005, 213658009, 213659001, 213660006, 216463005, 216464004, 216465003, 216466002, 216468001, 216469009, 216470005, 222059008, 241749009, 242829007, 269264002, 290157008, 290170004, 290171000, 290172007, 290179003, 290181001, 290182008, 290183003, 290193005, 290201006, 290202004, 290203009, 290204003, 295148000, 295161000, 295165009, 295170002, 295171003, 295172005, 295174006, 295175007, 295176008, 295184007, 295185008, 295186009, 295193008, 295194002, 295195001, 432353006, 461001000124107, 47836003, 60199004, 68099003, 74264003, 213663008, 213664002, 221753000, 221754006, 221755007, 221756008, 221757004, 221758009, 221759001, 221760006, 221761005, 221762003, 221763008, 222014000, 222015004, 222016003, 222017007, 222018002, 222019005, 222020004, 222021000, 222022007, 222023002, 222024008, 222612000, 222613005, 222614004, 222615003, 222616002, 222618001, 222619009, 222620003, 222621004, 222622006, 222623001, 290950009, 85337000, 212628009, 212639007, 111763008, 699012001, 221764002, 212637009, 212638004, 213664002, 216505004, 216515005, 216516006, 216517002, 219117005, 219310000, 221765001, 221766000, 221767009, 221768004, 221769007, 221770008, 221771007, 221772000, 221773005, 221774004, 222025009, 222026005, 222027001, 222028006, 222029003, 222030008, 222031007, 222032000, 222033005, 222034004, 222035003, 222624007, 222625008, 222626009, 222627000, 222628005, 222629002, 222630007, 222631006, 222632004, 222633009, 222634003, 269266000, 269267009, 290984008, 291100009, 44043001, 86726006, 1148567001, 212653007, 219118000, 219311001, 61438005, 699011008, 221786001, 212672007, 212673002, 213668004, 216544007, 216566000, 221787005, 221788000, 221789008, 221790004, 221791000, 221792007, 221793002, 221794008, 221795009, 221796005, 222048004, 222049007, 222050007, 222051006, 222052004, 222053009, 222054003, 222055002, 222056001, 222057005, 222058000, 222646004, 222647008, 222648003, 222649006, 222650006, 222651005, 222652003, 222653008, 222654002, 222655001, 222656000, 82225006, 32835006, 212687002, 213671007, 216567009, 216607002, 216608007, 213672000, 216615004, 22915003, 212716007, 213675003, 157680006, 157821006, 216616003, 43624006, 212732005, 212734006, 213678001, 157681005, 157822004, 27163006, 213682004, 15233006, 216551003, 16292008, 291246000, 291247009, 291248004, 212666007, 1149328002, 61803000, 216562003, 212668008, 212677001, 212678006, 216565001, 290406004, 291334002, 31523001, 212809004, 82782008, 67426006, 212806006, 212807002, 212808007, 212812001, 212819005, 212820004, 213687005, 10483001, 157683008, 157684002, 157685001, 157686000, 49153000, 212899006, 291284006, 291285007, 291286008, 66884007 |
|  | ICD-9 | 960.X - 979.X, E850.X - 858.X, E950.0 - E950.5, E962.0; E980.0-E980.5 |
|  | ICD-10 | T36.X -T50.X^1^ |
| Alcohol intoxication | SNOMED | 212809004, 82782008, 67426006, 212806006, 212807002, 212808007, 212812001, 212819005, 212820004, 213687005, 10483001, 157683008, 157684002, 157685001, 157686000, 49153000 |
|  | ICD-9 | 291.4, 303.0, 303.00, 303.01, 303.02, 303.03, 980, 980.1, 980.2, 980.3, 980.8, 980.9 |
|  | ICD-10 | F10.12, F10.120, F10.121, F10.129, F10.22, F10.220, F10.221, F10.229, F10.92, F10.920, F10.921, F10.929, T51.0, T51.0X, T51.0X1A, T51.0X1D, T51.0X1S, T51.0X2, T51.0X2A, T51.0X2D, T51.0X2S, T51.0X3A, T51.0X3S, T51.0X4A, T51.0X4D, T51.0X4S, T51.1, T51.1X1, T51.1X1A, T51.1X1S, T51.1X2A, T51.1X3A, T51.1X4A, T51.1X4S, T51.2, T51.2X1A, T51.2X1D, T51.2X1S, T51.2X2A, T51.2X2D, T51.2X3A, T51.2X3D, T51.2X4A, T51.2X4D, T51.2X4S, T51.3X1A, T51.3X2A, T51.3X4A, T51.8X, T51.8X1, T51.8X1A, T51.8X1D, T51.8X2, T51.8X2A, T51.8X2D, T51.8X3A, T51.8X3S, T51.8X4A, T51.8X4D, T51.8X4S, T51.9, T51.91, T51.91XA, T51.91XD, T51.91XS, T51.92XA, T51.92XD, T51.92XS, T51.93XA, T51.94, T51.94XA, T51.94XD, T51.94XS |
| Tobacco poisoning | ICD-9 | 989.84 |
|  | ICD-10 | T65.2X |
|  | SNOMED | 212899006 |

**1** not inclusive of codes relating to “underdosing” or “adverse effect”

**Supplemental Table 5.**List of codes used to define detoxification services

| **Code Type** | **Code(s)** |
| --- | --- |
| SNOMED | 56876005, 61480009, 827094004, 182969009, 87106005 |
| ICD-9 Procedure | 94.62, 94.65 |
| ICD-10 Procedure | HZ2ZZZZ |
| HCPCS | H0008, H0009, H0010, H0011, H0012, H0013, H0014, S9475 |

**Supplemental Table 6.**List of codes used to define outpatient SUD-related services

| **Code Type** | **Code(s)** |
| --- | --- |
| CPT | 90791, 90792, 90832, 90833, 90834, 90835, 90836, 90837, 90838, 90839, 90840, 90845, 90846, 90847, 90848, 90849, 90853, 90857, 90865, 90867, 90868, 90869, 90870,90871,90875,90876, 90880, 90900, 90901, 90902, 90904, 90906, 90908, 90910, 97003, 97004, 98960, 98961, 98962, 99058, 99078, 99201, 99202, 99203, 99204, 99205, 99211, 99212, 99213, 99214, 99215, 99241, 99242, 99243, 99244, 99245, 99341, 99342, 99343, 99344, 99345, 99347, 99348, 99349, 99350, 99382, 99383, 99384, 99385, 99386, 99387, 99392, 99393, 99394, 99395, 99396, 99397, 99401, 99402, 99403, 99404, 99408, 99409, 99411, 99412, 99420, 99441, 99442, 99443, 99843, 99490, 99495, 99496, 99510, 0359T, 0360T, 0361T, 0362T, 0363T, 0364T, 0365T, 0366T, 0367T, 0368T, 0369T, 0370T, 0371T, 0372T, 0373T,0374T |
| HCPCS | G0155, G0175, G0351, G0396, G0397, G0438, G0439, G0442, G0443, G0463, G0466, G0467, G0468, G0469, G0470, G0505, G0507, G0513, G0514, G0515, G2025, G2067, G2068, G2069, G2080, G2086, G2087, G2088, H0001, H0002, H0003, H0004, H0005, H0006, H0007, H0014, H0016, H0022, H0023, H0028, H0029, H0031, H0034, H0036, H0037, H0038, H0039, H0040, H0041, H0042, H0043, H0044, H0045, H0046, H0047, H0048, H0049, H0050, H1011, H2000, H2001, H2010, H2011, H2012, H2013, H2014, H2015, H2016, H2017, H2018, H2019, H2020, H2021, H2022, H2023, H2024, H2025, H2026, H2027, H2028, H2029, H2030, H2031, H2032, H2033, H2037, H5010, H5020, H5025, H5030, H5220, H5230, H5240, H5299, M0064, S9454, S9482, S9484, S9485, T1006, T1007, T1011, T1012, T1015, T1016, T1017, T1018, T1023, T1024, T1025, T1026, T1027, T1040, T1041, T2010, T2011, T2012, T2013, T2014, T2015, T2018, T2019, T2020, T2021, T2022, T2023, T2024, T2036, T2037, Z0001, Z0002 |

**Supplemental Table 7.**List of codes used to define general anesthetics^1^

| **Code Type** | **Code(s)** |
| --- | --- |
| NDC | 00054356699, 00069020910, 00069023420, 00069024810, 00074445651,  00143931010, 00143937910, 00143938010, 00143950610, 00143950710,  00409230504, 00409230505, 00409230517, 00409230521, 00409230550,  00409230801, 00409230802, 00409230822, 00409230849, 00409230850,  00409259603, 00409259605, 00409259653, 00409469924, 00409469930,  00409469933, 00409601025, 00409669501, 00409669502, 00517078001,  00641605701, 00641605710, 00641605910, 00641606010, 00641606110,  00641606325, 00904711422, 10019002801, 10019064134, 23155060031, 23155060041, 25021060820, 25021060850, 25021060851, 25021065502, 25021067410, 25021067420, 44567061110, 50474050015, 55150022110, 55150022220, 55390012610, 55390013702, 55390013801, 60201025101220, 60201025102002, 60201025102003, 60201025102005, 60201025102006, 60201025102011, 60201025102025, 61553019648, 63323026910, 63323026927, 63323026929, 63323026937, 63323026950, 63323026957, 63323026959, 63323026965, 63323026967, 63323026969, 63323026978, 63323026994, 63323027057, 63323027065, 63323041112, 63323041125, 63323041210, 63323041225, 64679076302, 66794001525, 66794001725, 67457018320, 67457090300, 68094076459, 68094076462, 68382054508, 70092102935,  70092117735, 70092131046, 70400010002020, 70400050001652, 70400050001660, 72100060002010, 72572043025, 72572043210, 75901800501,  76045000210, 76045000320 |
| MMSL Synonym | 108102, 12185, 12215, 12286, 12556, 12557, 12558, 12874, 131662, 13461, 159527, 1666798, 1666800, 1666814, 169627, 169628, 169629, 1808217, 18413, 184407, 184409, 184410, 184411, 184412, 187714, 200646, 2007, 203101, 203128, 2152, 2173500, 21852, 220599, 2227, 22619, 22620, 23020, 23800, 2434, 253981, 254718, 262452, 26496, 27498, 27536, 2762, 27959, 284,  284333, 284334, 285138, 285232, 311702, 319984, 32073, 333907, 335897, 340762, 3682, 374, 385156, 40926, 4177, 422410, 4465, 45875, 46012,  47582, 47583, 65, 6960, 73245, 8782, 9154 |
| MMSL Drug ID | d00267, d00301, d00388, d00929, d00931, d00933, d02374, d03845, d07370 |

**1** from outpatient encounters

**Supplemental Table 8.** List of codes used to define procedures

| **Procedure Department** | **Procedure Type** | **Code Type** | **Codes** |
| --- | --- | --- | --- |
| Orthopedics | Carpal tunnel | CPT | 20526, 25000, 25001, 29848, 64721 |
|  |  | ICD-9 | 78.44 |
|  |  | SNOMED | 171837008, 171839006, 171840008, 171841007 |
|  |  | ICD-10 | OPNNXXX, 0PNMXXX |
|  | Hip replacement | CPT | 01215, 27125, 27130, 27132, 27134, 27137, 27138, 27236, 27258, 01214 |
|  |  | ICD-9 | 81.51 |
|  |  | SNOMED | 15163009, 179304004, 179326003, 265106004, 265157000, 265160007, 314491003, 32581000, 340922009, 443435007, 450813004, 52734007, 711202009, 713686009, 770606008, 386649003 |
|  |  | ICD-10 | 0SR901A, 0SR901Z, 0SR9029, 0SR902A, 0SR902Z, 0SR9039, 0SR903A, 0SR903Z, 0SR9049, 0SR904A, 0SR904Z, 0SR9069, 0SR906A, 0SR906Z, 0SR907Z, 0SR90EZ, 0SR90J9, 0SR90JA, 0SR90JZ, 0SR90KZ, 0SRB019, 0SRB01A, 0SRB01Z, 0SRB029, 0SRB02A, 0SRB02Z, 0SRB039, 0SRB03A, 0SRB03Z, 0SRB049, 0SRB04A, 0SRB04Z, 0SRB069, 0SRB06A, 0SRB06Z, 0SRB07Z, 0SRB0EZ, 0SRB0J9, 0SRB0JA, 0SRB0JZ, 0SRB0KZ |
|  | Knee osteotomy | CPT | 27448, 27450, 27454, 27455, 27457, 27705, 27709, 27712, 01484 |
|  |  | ICD-9 | 77.27 |
|  |  | SNOMED | 171837008, 171839006, 171840008, 39585008, 56582000, 171841007 |
|  |  | ICD-10 | 0G8HXXX, 0G8JXXX, 0G8KXXX, 0Q8GXXX |
|  | ACL reconstruction | CPT | 29888 |
|  |  | ICD-9 | 81.45 |
|  |  | SNOMED | 239431009, 313315002, 314284003, 37422000, 391101007, 444889008, 54419004, 55244002, 239426007 |
|  |  | ICD-10 | 0MQPXXX, 0MQNXXX |
|  | Lumbar interbody arthrodesis | CPT | 22630, 22633, 22558 |
|  |  | SNOMED | 10420000, 178603007, 178646005, 178647001, 265717009, 265718004, 276850007, 277764006, 278659001, 34238000, 428925003, 429541002, 448025007, 448240004, 448514006, 449448002, 705043000, 719217005, 85926009, 50172003 |
|  | Fracture or dislocation of hip and femur | CPT | 27230, 27232, 27235, 27236, 27246, 27248, 27130 |
|  |  | SNOMED | 179051005, 179064006, 179104006, 179105007, 179136004, 179137008, 179138003, 179139006, 179159005, 180315008, 429891009, 432304006, 439995008, 440302000, 440304004, 440376001, 440417002, 442957002, 708898008, 708900005, 708903007, 448243002 |
|  | Knee arthroplasty | CPT | 27446, 27447, 27486, 27487, 27488, 27438 |
|  |  | ICD-9 | 0.8 |
|  |  | SNOMED | 265170009, 265172001, 443681002, 443682009, 713687000, 609588000 |
|  |  | ICD-10 | 0SPC09Z, 0SPC0JZ, 0SPC48Z, 0SPC4JZ, 0SPD08Z, 0SPD09Z, 0SPD0JZ, 0SPD48Z, 0SPD4JZ, 0SRC069, 0SRC06A, 0SRC06Z, 0SRC0J9, 0SRC0JA, 0SRC0JZ, 0SRD069, 0SRD06A, 0SRD06Z, 0SRD0J9, 0SRD0JA, 0SRD0JZ, 0SPC08Z |
| Neurology | Spinal fusion | CPT | 22533, 22534, 22548, 22551, 22552, 22554, 22556, 22558, 22585, 22586, 22590, 22595, 22600, 22610, 22612, 22614, 22630, 22632, 22633, 22634, 22800, 22802, 22804, 22808, 22810, 22812, 22830, 22840, 22841, 22842, 22843, 22844, 22845, 22846, 22847, 22848, 22532 |
|  |  | SNOMED | 10420000, 1253002, 178603007, 178647001, 239546004, 239547008, 25789004, 265717009, 277764006, 278659001, 279526001, 359586003, 428547005, 428925003, 429769008, 441715004, 448514006, 448807001, 448808006, 448918002, 449048009, 449243009, 59323008, 705043000, 709292007, 709504001, 81099000, 50172003 |
|  |  | ICD-10 | 0RG00J, 0RG00K, 0RG037, 0RG03J, 0RG047, 0RG04J, 0RG04K, 0RG007, 0RG03K |
|  | Laminectomy | CPT | 22101, 22102, 22103, 62351, 63001, 63003, 63005, 63011, 63012, 63015, 63016, 63017, 63020, 63035, 63045, 63046, 63047, 63048, 63170, 63172, 63173, 63185, 63190, 63191, 63194, 63195, 63196, 63197, 63198, 63199, 63200, 63250, 63251, 63252, 63265, 63266, 63267, 63268, 63270, 63271, 63272, 63273, 63275, 63276, 63277, 63278, 63280, 63281, 63282, 63283, 63285, 63286, 63287, 63290, 63295, 63300, 63301, 63302, 63303, 63304, 63305, 63306, 63307, 63308, 63655, 22100 |
|  |  | SNOMED | 197889000, 21089001, 2564002, 260647003, 260648008, 261540001, 283165009, 284088004, 359586003, 387731002, 429377005, 438362003, 440211003, 445429009, 448234006, 47478002, 709217002, 83471008, 83948001 |
|  |  | ICD-10 | 0R533ZZ, 0R534ZZ, 0R550ZZ, 0R553ZZ, 0R554ZZ, 0R590ZZ, 0R593ZZ, 0R594ZZ, 0R5B0ZZ, 0R5B3ZZ, 0R5B4ZZ, 0RB30ZZ, 0RB33ZZ, 0RB34ZZ, 0RB50ZZ, 0RB53ZZ, 0RB54ZZ, 0RB90ZZ, 0RB93ZZ, 0RB94ZZ, 0RBB0ZZ, 0RBB3ZZ, 0RBB4ZZ, 0RT30ZZ, 0RT40ZZ, 0RT50ZZ, 0RT90ZZ, 0RTB0ZZ, 0S520ZZ, 0S523ZZ, 0S524ZZ, 0S540ZZ, 0S543ZZ, 0S544ZZ, 0SB20ZZ, 0SB23ZZ, 0SB24ZZ, 0SB40ZZ, 0SB43ZZ, 0SB44ZZ, 0ST20ZZ, 0ST40ZZ, 0R530ZZ |
|  | Diskectomy | CPT | 22224, 22857, 22858, 62287, 63064, 63075, 22856 |
|  |  | ICD-9 | 80.5 |
|  |  | SNOMED | 178618008, 178619000, 178620006, 178623008, 178624002, 178625001, 178626000, 265714002, 307720007, 309702009, 450837004, 448234006 |
|  | Carotid endarterectomy | CPT | 35390, 35301 |
|  |  | ICD-9 | 38.12 |
|  |  | SNOMED | 175367001, 233296007, 233297003, 233298008, 276949008, 276950008, 276951007, 405407008, 405408003, 405409006, 405411002, 405412009, 66951008 |
|  |  | ICD-10 | 03CH4ZZ, 03CJ0ZZ, 03CJ4ZZ, 03CK0ZZ, 03CK4ZZ, 03CL0ZZ, 03CL4ZZ, 03CM0ZZ, 03CM4ZZ, 03CN0ZZ, 03CN4ZZ, 03CH0ZZ |
|  | Vertebroplasty | CPT | 22511, 22512, 22510 |
|  |  | ICD-9 | 81.65 |
|  |  | SNOMED | 401226007, 431206007, 431328005, 431496002, 431555002, 431911003, 432037000, 433034001, 433220005, 432637001 |
|  |  | ICD-10 | 0PU33JZ, 0PU34JZ, 0PU43JZ, 0PU44JZ, 0QU03JZ, 0QU13JZ, 0QU14JZ, 0QU04JZ |
| Obstetrics / Gynecology | Cesarean section | CPT | 01968, 58611, 59100, 59510, 59514, 59515, 59525, 59610, 59612, 59614, 59618, 59620, 59622, 01961 |
|  |  | ICD-9 | 649.81, 649.82, 669.70, 669.71, 74.1, 74.2, 74.4, 74.9, 74.99, O75.82, O82.XX, 74.0 |
|  |  | SNOMED | 156258008, 177141003, 177142005, 177143000, 177144006, 177145007, 200148001, 236985002, 236986001, 236987005, 236988000, 236989008, 236990004, 267357005, 274130007, 288042004, 41059002, 450483001, 450484007, 57271003, 709004006, 736018001, 736026009, 788180009, 84195007, 89053004, 11466000 |
|  | Hysterecomy | CPT | 00944, 01962, 01963, 01969, 45126, 58150, 58152, 58180, 58200, 58210, 58240, 58260, 58262, 58263, 58267, 58270, 58275, 58280, 58285, 58290, 58291, 58292, 58293, 58294, 58541, 58542, 58543, 58544, 58545, 58546, 58548, 58550, 58552, 58553, 58554, 58570, 58571, 58572, 58573, 58575, 58661, 58951, 58952, 58953, 58954, 58956, 59525, 00846 |
|  |  | SNOMED | 431316002, 11050006, 112918004, 116140006, 116141005, 116142003, 116143008, 120038005, 12398007, 176795006, 176895001, 17744000, 236884004, 236887006, 236888001, 24068006, 265056007, 265065000, 288042004, 288043009, 309880009, 359971002, 359974005, 359977003, 359983000, 36384005, 387643005, 387644004, 41059002, 413145007, 427107006, 447237002, 448539002, 449727003, 450692005, 54130005, 54261007, 54490004, 63516002, 699789005, 708985003, 739671004, 739672006, 740514001, 740515000, 75835007, 762625001, 767610009, 88144003, 88218008, 236886002 |
|  | Myomectomy | CPT | 45108, 58145, 58146, 58545, 58546, 58140 |
|  |  | SNOMED | 195459001, 236901008, 236902001, 265059000, 306966000, 42010004, 428652006, 446804002, 450559006 |
|  | Dilation and curettage | CPT | 57522, 57558, 58120, 57520 |
|  |  | SNOMED | 176832001, 176833006, 265062002, 274972007, 274973002, 28379004, 287927002, 391998006, 74608009, 76810006, 11401008 |
| Otolaryngology | Thyroidectomy | CPT | 60210, 60212, 60220, 60225, 60240, 60252, 60254, 60260, 60270, 60271, 60512, 60200 |
|  |  | ICD-9 | 06.4, 06.39 |
|  |  | SNOMED | 171977001, 237486002, 24443003, 27210007, 274005006, 302338000, 30956003, 359884005, 50339005, 52814001, 52826006, 53533006, 708915006, 712978001, 719753008, 744854002, 744855001, 91276009, 13619001 |
|  |  | ICD-10 | 0GBG0ZX, 0GBG0ZZ, 0GBG3ZX, 0GBG3ZZ, 0GBG4ZX, 0GBG4ZZ, 0GBH0ZX, 0GBH0ZZ, 0GBH3ZX, 0GBH3ZZ, 0GBH4ZX, 0GBH4ZZ, 0GTK0ZZ |
|  | Reflux surgery | CPT | 43325, 43327, 43328, 43280 |
|  |  | SNOMED | 265358004, 265359007, 265360002, 359890009, 359893006, 7161000179101, 78656005, 265357009 |
|  | Otoplasty | CPT | 69300 |
|  |  | SNOMED | 120135005, 120136006, 16443000, 172624005, 19934003, 210805003, 212525003, 232161006, 232162004, 23235002, 33901004, 350515003, 417224006, 50691002, 84705009, 52577005 |
| General Surgery | Laparoscopic appendectomy | CPT | 44979, 44970 |
|  |  | ICD-9 | 47.11, 47.01 |
|  |  | SNOMED | 174041007, 307581005, 708876004, 6025007 |
|  |  | ICD-10 | 0DTJ4ZZ |
|  | Laparoscopic cholecystectomy | CPT | 47563, 47564, 47562 |
|  |  | ICD-9 | 51.24, 51.23 |
|  |  | SNOMED | 20630000, 450499007, 67557008, 713872007, 45595009 |
|  |  | ICD-10 | 0FB44ZZ, 0FB48ZZ, 0FT44ZZ |
|  | Mastectomy | CPT | 19125, 19126, 19294, 19297, 19300, 19301, 19302, 19303, 19304, 19305, 19306, 19307, 19120 |
|  |  | ICD-9 | 85.42, 85.43, 85.44, 85.41 |
|  |  | SNOMED | 12708000, 14693006, 14714006, 172044000, 172049005, 172111009, 20486005, 22418005, 237367009, 237368004, 237370008, 237393002, 237394008, 237400005, 265255003, 274957008, 27865001, 287653007, 307796007, 310638008, 318190001, 35212009, 35482003, 359728003, 359731002, 359734005, 359740003, 384723003, 392021009, 395702000, 406505007, 428554004, 428564008, 428571003, 429400009, 446109005, 446420001, 447135002, 447168009, 447421006, 451201000124106, 451211000124109, 456903003, 52314009, 59620004, 60633004, 6189002, 62347003, 64368001, 66398006, 70183006, 72432009, 72577009, 726429001, 726430006, 726434002, 726435001, 726436000, 726437009, 735085002, 735086001, 736751004, 736752006, 736753001, 736754007, 741009001, 741010006, 741018004, 741019007, 76468001, 770108008, 172043006 |
|  |  | ICD-10 | 0HTU0ZZ, 0HTV0ZZ, 0HTT0ZZ |
|  | Open inguinal hernia repair | CPT | 49568 |
|  |  | ICD-9 | 53.02, 53.03, 53.04, 53.01 |
|  |  | SNOMED | 771714004, 771716002, 771715003 |
|  |  | ICD-10 | 0YQ50ZZ, 0YQA0ZZ, 0YU507Z, 0YU50JZ, 0YU50KZ, 0YU607Z, 0YU60JZ, 0YU60KZ, 0YUA07Z, 0YUA0JZ, 0YUA0KZ, 0YQ60ZZ |
|  | Open appendectomy | CPT | 44955, 44960, 44950 |
|  |  | ICD-9 | 47.09 |
|  |  | SNOMED | 443935000 |
|  |  | ICD-10 | 0DTJ0ZZ |
| Bariatrics | Sleeve gastrectomy | CPT | 43775 |
|  |  | ICD-9 | 43.89, 43.82 |
|  |  | SNOMED | 427074001, 427980007, 87604009 |
|  |  | ICD-10 | 0DB63Z3, 0DB64Z3, 0DB67Z3, 0DB68Z3, 0DB60Z3 |
|  | Open small bowel resection | CPT | 44005, 44125, 44158, 44180, 44625, 44626 |
|  | Colectomy | CPT | 44140, 44141, 44143, 44144, 44145, 44146, 44147, 44150, 44151, 44155, 44156, 44157, 44158, 44160, 44204, 44205, 44206, 44207, 44208, 44210, 44211, 44212, 44213, 44626, 44701, 45121, 45126, 44139 |
|  |  | ICD-9 | 45.79, 45.71 |
|  |  | SNOMED | 13327002, 174059005, 174081000, 26390003, 274025005, 275017002, 276190007, 287815004, 301699000, 304587000, 307507006, 307654002, 307659007, 307662005, 307666008, 31130001, 32044008, 359571009, 36192008, 386197003, 425851003, 426699005, 43075005, 443480008, 44378008, 443909001, 444165004, 445884009, 446747005, 448050005, 450462007, 54164009, 75312003, 75404005, 771568007, 787109009, 787874000, 80294005, 84604002, 84952009, 87279008, 23968004 |
|  |  | ICD-10 | 0DBE3ZZ, 0DBE7ZZ, 0DBE8ZZ, 0DBGFZZ, 0DBLFZZ, 0DBMFZZ, 0DBNFZZ, 0DTMFZZ, 0DBE0ZZ |
|  | Bariatric surgery | CPT | 43644, 43645, 43659, 43771, 43772, 43773, 43774, 43775, 43842, 43843, 43844, 43845, 43846, 43847, 43770 |
|  |  | SNOMED | 2391000175104, 430715008 |
|  | Proctectomy | CPT | 44156, 44157, 44158, 44210, 44211, 44212, 45110, 45111, 45112, 45113, 45114, 45116, 45119, 45120, 45121, 45123, 45126, 45395, 45397, 44155 |
|  |  | SNOMED | 11626008, 174240003, 235364003, 303584001, 44751009, 55966001, 77878003, 787108001, 787109009, 31130001 |
| Colo-Rectal | Colostomy | CPT | 44143, 44144, 44146, 44160, 44188, 44205, 44206, 44208, 44320, 44322, 44340, 44345, 44346, 44388, 44390, 44391, 44392, 44141 |
|  |  | ICD-9 | 46.1X |
|  |  | SNOMED | 11626008, 16564004, 17671008, 24221008, 25196007, 261030007, 261072008, 261765002, 261766001, 297223007, 299681007, 299682000, 307645000, 307657009, 35535008, 37828009, 386651004, 386828002, 386829005, 387606008, 387607004, 398740003, 4044002, 44642007, 447982008, 448653006, 46295004, 49440008, 49924002, 51977003, 54629001, 62069000, 64215005, 73509004, 75312003, 89339008, 89642006, 9905009, 46070005 |
|  |  | ICD-10 | 0D1K0J4, 0D1K0K4, 0D1K0Z4, 0D1K3J4, 0D1K474, 0D1K4J4, 0D1K4K4, 0D1K4Z4, 0D1K874, 0D1K8J4, 0D1K8K4, 0D1K8Z4, 0D1L0J4, 0D1L0L4, 0D1L0Z4, 0D1L3J4, 0D1L474, 0D1L4J4, 0D1L4L4, 0D1L4Z4, 0D1L874, 0D1L8J4, 0D1L8L4, 0D1L8Z4, 0D1M074, 0D1M0J4, 0D1M0M4, 0D1M0Z4, 0D1M3J4, 0D1M474, 0D1M4J4, 0D1M4M4, 0D1M4Z4, 0D1M874, 0D1M8J4, 0D1M8M4, 0D1M8Z4, 0D1N074, 0D1N0J4, 0D1N0N4, 0D1N0Z4, 0D1N3J4, 0D1N474, 0D1N4J4, 0D1N4N4, 0D1N4Z4, 0D1N874, 0D1N8J4, 0D1N8N4, 0D1N8Z4, OD1L074, 0D1K074 |
|  | Hemorrhoidectomy | CPT | 46255, 46257, 46258, 46260, 46261, 46262, 46250 |
|  |  | ICD-9 | 49.46 |
|  |  | SNOMED | 12896001, 22432007, 235389009, 235390000, 235391001, 287802008, 442792000, 49645007, 5796001, 61498008, 24496007 |
|  |  | ICD-10 | 06BY3ZC, 06BY4ZC, 06BY0ZC |
|  | Rectopexy | CPT | 45402, 45540, 45541, 45550, 57280, 57425, 45400 |
|  |  | SNOMED | 112877004, 2234009, 235376003, 235377007, 235378002, 235379005, 265412004, 265417005, 265418000, 265420002, 275011001, 29205000, 30402002, 440032004, 440033009, 444688002, 708642005, 73996007, 75894009, 782836001, 10259007 |
| Transplant & Hepatobilary Surgery | Kidney transplant | CPT | 0088U, 50220, 50225, 50230, 50234, 50236, 50240, 50300, 50320, 50323, 50325, 50327, 50328, 50329, 50340, 50360, 50365, 50370, 50380, 00868 |
|  |  | ICD-9 | 55.69 |
|  |  | SNOMED | 236436003, 428575007, 714153000 |
|  |  | ICD-10 | 0TY00Z0, 0TY00Z1, 0TY00Z2, 0TY10Z1, 0TY10Z2, 0TY10Z0 |
|  | Whipple | CPT | 48150, 48152, 48153, 48154, 00794 |
|  |  | SNOMED | 116031009, 116242006, 265458003, 287846000, 265459006 |
|  | Distal pancreatectomy | CPT | 48145, 48146, 48160, 48140 |
|  |  | SNOMED | 235468001, 235469009, 235470005, 265461002, 60194009, 91516004, 401004 |
|  | Liver transplant | CPT | 47133, 47135, 47140, 47141, 47142, 47143, 47144, 47145, 47146, 47147, 00796 |
|  |  | ICD-9 | 50.59, 50.51 |
|  |  | SNOMED | 174425003, 174426002, 174427006, 27280000, 28009009, 426356008, 18027006 |
|  |  | ICD-10 | 0FY00Z1, 0FY00Z2, 0FY00Z0 |
|  | Heart transplant | CPT | 33927, 33928, 33929, 33930, 33933, 33935, 33944, 33945, 00580 |
|  |  | ICD-9 | 37.51 |
|  |  | SNOMED | 174802006, 174808005, 174809002, 232973007, 232974001, 32477003, 405768001, 47058000, 32413006 |
|  |  | ICD-10 | 02YA0Z1, 02YA0Z2, 02YA0Z0 |
| Cardiac | Aortic valve repair | CPT | 33361, 33362, 33363, 33364, 33365, 33366, 33390, 33391, 33400, 33414, 33415, 33416, 33417, 33470, 33471, 33474, 33602, 33782, 33783, 33852, 33853, 33858, 33859, 33864, 93591, 93592, 92986 |
|  |  | SNOMED | 174927000, 232823005, 232827006, 232843004, 265473002, 357575008, 444812008, 52247003, 232848008 |
|  | Coronary artery bypass graft | CPT | 33361, 33362, 33363, 33364, 33365, 33366, 33511, 33512, 33513, 33514, 33516, 33510 |
|  |  | SNOMED | 10326007, 119565001, 17073005, 232717009, 232720001, 232721002, 232722009, 232723004, 232724005, 309814006, 3546002, 359601003, 39202005, 405598005, 405599002, 67166004, 736966005, 736967001, 736968006, 736969003, 74371005, 82247006, 8876004, 232719007 |
|  | Implantable cardioverter-defibrillator | CPT | 0575T, 0576T, 0577T, 0578T, 0579T, 33223, 33224, 33230, 33241, 33249, 33262, 33263, 33264, 4470F, 0571T |
|  |  | ICD-9 | 37.96, 37.95 |
|  |  | SNOMED | 360056009, 450651000124104, 462655000, 463257006, 465460004, 465652008, 465790005, 468542000, 704707009, 72506001 |
|  |  | ICD-10 | 02H60KZ, 02H63KZ, 02H64KZ, 02H70KZ, 02H73KZ, 02H74KZ, 02HK0KZ, 02HK3KZ, 02HK4KZ, 02HL0KZ, 02HL3KZ, 02HL4KZ, 02PA0MZ, 02PA3MZ, 02PA4MZ, 02PAXMZ, 0JH609Z, 0JH60FZ, 0JH638Z, 0JH639Z, 0JH63FZ, 0JH808Z, 0JH809Z, 0JH838Z, 0JH839Z, 0JPT0FZ, 0JPT0PZ, 0JPT3FZ, 0JPT3PZ, 0JH608Z |
|  | Balloon angioplasty | CPT | 37246, 37248, 61630, 92921, 92924, 92925, 92928, 92929, 92933, 92934, 92937, 92938, 92941, 92943, 92944, 92997, 92998, 92920 |
|  |  | SNOMED | 175066001, 175354004, 230923002, 233260008, 233269009, 233284000, 233286003, 233291002, 425979008, 426674009, 428901006, 429639007, 429933006, 433711000, 433734009, 434433007, 707828002, 713155002, 713169002, 86274005 |
|  | Catheter ablation | CPT | 93462, 93650, 93653, 93654, 93655, 93656, 93657 |
|  |  | SNOMED | 702181002, 702209009, 702210004, 704706000, 717300007, 705732002 |
| Urology | Prostatectomy | CPT | 00908, 00914, 52601, 52630, 52640, 55801, 55810, 55812, 55815, 55821, 55831, 55840, 55842, 55845, 55866, 00865 |
|  |  | ICD-9 | 60.4, 60.5, 60.6, 60.3 |
|  |  | SNOMED | 176106009, 176258007, 176260009, 176261008, 176262001, 176263006, 176267007, 176288003, 19149007, 21190008, 236209003, 236211007, 26294005, 28579000, 30426000, 314202001, 36253005, 37851009, 41371003, 41416003, 427985002, 446445003, 57525009, 65551008, 67598001, 68986004, 699077003, 708622001, 708919000, 72388004, 81232004, 83154001, 85768003, 87795007, 8782006, 90199006, 91531008, 90470006 |
|  |  | ICD-10 | 0VT04ZZ, 0VT07ZZ, 0VT08ZZ, 0VT00ZZ |
|  | Orchiopexy | CPT | 54640, 54650, 54692, 00930 |
|  |  | ICD-9 | 62.5 |
|  |  | SNOMED | 12410002, 15791006, 176434006, 176437004, 176438009, 236340008, 25041004, 301774009, 301776006, 387668007, 387669004, 387671004, 447082006, 719441005, 719444002, 735023007, 735024001, 762902004, 762903009, 767331003, 767332005, 767378002, 767379005, 89844005, 85419002 |
|  |  | ICD-10 | 0VS93ZZ, 0VS94ZZ, 0VS98ZZ, 0VSB0ZZ, 0VSB3ZZ, 0VSB4ZZ, 0VSB8ZZ, 0VSC0ZZ, 0VSC3ZZ, 0VSC4ZZ, 0VSC8ZZ, 0VS90ZZ |
|  | Vasectomy | CPT | 0421T, 52402, 52601, 52630, 52647, 52648, 52649, 55250, 55400, 55801, 55821, 55831, 00921 |
|  |  | ICD-9 | 63.73 |
|  |  | SNOMED | 17293009, 276487005, 287662009, 47655001, 22523008 |
|  |  | ICD-10 | 0VBQ3ZZ, 0VBQ4ZZ, 0VBQ8ZZ, 0VBQ0ZZ |
|  | Cystoplasty | CPT | 5180050825 |
|  |  | ICD-9 | 57.89, 57.87 |
|  |  | SNOMED | 176117005, 176118000, 176165006, 176166007, 277837003, 361256009, 361257000, 386652006, 48007004 |
|  |  | ICD-10 | 0TQBXXX, 0TRBXXX, 0TUBXXX |
|  | Lithotripsy | CPT | 00873, 43265, 47544, 50080, 50081, 50590, 51065, 52325, 52353, 52356, 00872 |
|  |  | ICD-9 | 98.52, 98.51 |
|  |  | SNOMED | 175951002, 175952009, 175953004, 176050007, 176058000, 176064007, 20042009, 235525000, 235547000, 236172004, 236173009, 236179008, 236180006, 236190003, 24376003, 274446006, 274447002, 274448007, 287586004, 37597002, 386189005, 386198008, 404812006, 42041003, 425577000, 427001005, 427278007, 431401004, 431494004, 431847003, 432032006, 432036009, 432649000, 432650000, 432880008, 433137006, 44345001, 446125000, 446297008, 446587003, 446685001, 44817001, 4501000087102, 450497009, 450498004, 4511000087100, 53514001, 61417004, 713163001, 713164007, 82920008, 90705003, 133864008 |
|  |  | ICD-10 | 0FF4XZZ, 0TF3XZZ, 0TF4XZZ, 0TF7XZZ, 0TFBXZZ, 0TFCXZZ, 0TF6XZZ |
| Vascular Surgery | Angioplasty | CPT | 35879, 36903, 36905, 36906, 36907, 36908, 37186, 37215, 37216, 37217, 37218, 37220, 37221, 37222, 37223, 37224, 37225, 37226, 37227, 37228, 37229, 37230, 37231, 37232, 37233, 37234, 37235, 37236, 37237, 37238, 37239, 37246, 37247, 37248, 37249, 61630, 61635, 61640, 61641, 61642, 36902 |
|  |  | SNOMED | 233259003, 405326004, 405407008, 405408003, 405409006, 405411002, 405415006, 417884003, 419014003, 420026003, 420046008, 429287007 |
|  | Atherectomy | CPT | 0234T, 0235T, 0236T, 0237T, 0238T, 37227, 37229, 37231, 37233, 37235, 92924, 92933, 92937, 92941, 92943, 37225 |
|  |  | SNOMED | 126062009, 15101009, 175764006, 175882004, 20500004, 230922007, 232727003, 232728008, 232729000, 232980009, 232988002, 232996007, 233002007, 233214006, 233258006, 233261007, 233262000, 233263005, 233266002, 233268001, 233270005, 233272002, 233275000, 233277008, 233290001, 233476002, 24088005, 257765008, 257766009, 261562001, 27659003, 27672002, 276861004, 276890005, 276897008, 29843007, 301429003, 301430008, 312610006, 346007006, 359551000, 373360004, 373363002, 397194000, 405395009, 405396005, 405410001, 405454007, 405593001, 410023005, 41339005, 415069007, 418076001, 418269003, 419224002, 419801000, 419806005, 419827000, 425712000, 428811000, 429287007, 429813006, 429814000, 429815004, 429816003, 431253005, 431522007, 431523002, 431581003, 431752001, 431759005, 431932006, 432640001, 432866001, 432867005, 441540009, 442881005, 443038001, 443513002, 446051001, 446052008, 446878003, 448754006, 448798006, 51158001, 5431005, 58622000, 609167004, 65659003, 6832004, 698740005, 70131004, 708872002, 708873007, 708874001, 709019007, 709021002, 709862009, 709863004, 709981002, 710161003, 711222008, 723683000, 726555002, 7414004, 76611008 |
|  |  | ICD-10 | 02C13Z7, 02C23Z7, 02C33Z7, 02C03Z7 |
|  | Endarterectomy | CPT | 03CL0ZZ, 33501, 33502, 33503, 33504, 33505, 33506, 33507, 33572, 33916, 35301, 35302, 35303, 35304, 35305, 35306, 35311, 35321, 35331, 35341, 35351, 35355, 35361, 35363, 35371, 35372, 35390, 38.1X, 33500 |
|  |  | SNOMED | 14259004, 15023006, 15853003, 16589005, 16903003, 17437005, 175346001, 175367001, 175418002, 175419005, 175423002, 175424008, 175425009, 175477003, 175514002, 175689005, 175690001, 19606007, 19953008, 20738002, 21260009, 232726007, 233296007, 233297003, 233298008, 233299000, 233300008, 233301007, 233302000, 233303005, 233310004, 240923000, 240925007, 26076006, 265517007, 275064002, 276921001, 276922008, 276923003, 276925005, 276926006, 276927002, 276928007, 276929004, 276930009, 276931008, 276932001, 276933006, 276937007, 276939005, 276942004, 276946001, 276949008, 276950008, 307800001, 35653006, 35888008, 37199006, 38260008, 38863001, 40455006, 405337003, 405338008, 405343001, 405389008, 405392007, 405395009, 405396005, 405397001, 405398006, 405399003, 405400005, 405401009, 405402002, 405403007, 405406004, 405407008, 405410001, 405411002, 405412009, 405454007, 405455008, 405456009, 415071007, 415072000, 43418007, 44620006, 447746007, 448341002, 448534007, 448939009, 450513009, 450696008, 49187005, 52005005, 66951008, 68324008, 71198003, 72396009, 72672007, 74556005, 7642007, 77163008, 77445003, 82467004, 83662005, 84594003, 85448008, 87742009, 90499001, 392031002 |
|  | Endovascular stent graft | CPT | 34813, 37221, 37223, 37226, 37227, 37230, 37231, 33891 |
|  |  | SNOMED | 434159001, 434378006, 433591001 |
|  | Bypass surgery | CPT | 33315, 33916, 35523, 35535, 35537, 35538, 35539, 35540, 35570, 35632, 35633, 35634, 35637, 35638, 35686, 35883, 35884, 33310 |
|  |  | SNOMED | 10190003, 10326007, 119565001, 14323007, 17073005, 175045009, 232717009, 232719007, 232720001, 232721002, 232722009, 232723004, 232724005, 29819009, 309814006, 3546002, 359601003, 39202005, 405598005, 405599002, 67166004, 736962007, 736963002, 736964008, 736965009, 736966005, 736967001, 736968006, 736969003, 736970002, 736971003, 736972005, 736973000, 74371005, 82247006, 8876004, 90487008, 175021005 |
| Plastic Surgery | Liposuction | CPT | 15771, 15772, 15773, 15774, 15819, 15876, 15877, 15878, 15879, 0566T |
|  |  | ICD-9 | 86.9 |
|  |  | SNOMED | 15039004, 177252003, 177710009, 444162001, 75674002, 8085000, 302441008 |
|  |  | ICD-10 | 0JDXXXX |
|  | Rhinoplasty | CPT | 30410, 30420, 30430, 30435, 30450, 30460, 30462, 30400 |
|  |  | SNOMED | 11759002, 172761005, 172773005, 172786001, 172796005, 172842002, 172848003, 172849006, 172850006, 20805001, 232463004, 232464005, 232466007, 232467003, 232468008, 232482008, 265021008, 265023006, 315312002, 32010001, 360754002, 360762005, 415302008, 45474001, 52103001, 53356001, 704040006, 704041005, 704053009, 72009003, 72767005, 736513001, 8142006, 82862002, 172765001 |
|  | Breast augmentation/implant | CPT | 19318, 19325, 19328, 19330, 19350, 19355, 19357, 19361, 19364, 19367, 19368, 19369 |
|  |  | SNOMED | 172079003, 172080000, 172081001, 287649005, 287650005, 287651009, 443180000, 45467006, 22890008 |
|  | Abdominoplasty | CPT | 15847, 17999, 15830 |
|  |  | ICD-9 | 86.89 |
|  |  | SNOMED | 177252003, 240972007, 240973002, 240974008, 240975009, 442209001, 59716009, 177250006 |
|  |  | ICD-10 | 0W0F0ZZ |
| Burn Surgery | Laryngoplasty | CPT | 31552, 31553, 31554, 31580, 31582, 31584, 31587, 31588, 31591, 31599, 31551 |
|  |  | SNOMED | 38724002 |
|  | Escharotomy | CPT | 15003, 15004, 15005, 16035, 16036, 15002 |
|  |  | SNOMED | 177652008, 177653003, 177654009, 177656006, 177659004, 240992000, 449665001, 449666000, 449667009, 449668004, 70177008 |
| **Other** | Colonoscopy | CPT | 44388, 44389, 44390, 44391, 44392, 44393, 44394, 44397, 44401, 44402,  44403, 44404, 44405, 44406, 44407, 44408, 45355, 45378, 45379, 45380,  45381, 45382, 45383, 45384, 45385, 45386, 45387, 45388, 45389, 45390,  45391, 45392, 45393, 45398,  45330, 45331, 45332, 45333, 45334, 45335, 45337, 45338, 45339, 45340, 45341,  45342, 45345, 45346, 45347, 45349, 45350, 74261, 74262, 74263 |
|  |  | HCPCS | G0105, G0120, G0121 |
|  | Cataract Surgery | CPT | 66984, 66983, 66982 |

**Supplemental Table 9.** List of codes used to define Charlson Comorbidity Index (CCI)

| CCI condition | Code type | Codes |
| --- | --- | --- |
| Myocardial infarction | ICD-9 | 410.X, 411.0, 411.89, 412.X, 414.01, 414.8, 414.9, 429.2, 429.4, 429.6, 429.71, 429.79, 997.1 |
|  | SNOMED | 10273003, 103011000119106, 1077002, 129574000, 15712841000119100, 15712881000119105, 15712921000119103, 15712961000119108, 15713041000119103, 15713081000119108, 15713121000119105, 15713161000119100, 15713201000119105, 15963181000119104, 15990001, 161502000, 161503005, 164865005, 164867002, 164868007, 164869004, 164870003, 164871004, 17531000119105, 1755008, 194802003, 194809007, 194856005, 194857001, 194858006, 194861007, 194862000, 194863005, 194865003, 194866002, 194867006, 194868001, 22298006, 23311000119105, 233825009, 233826005, 233827001, 233828006, 233829003, 233830008, 233831007, 233832000, 233833005, 233834004, 233835003, 233836002, 233837006, 233838001, 233839009, 233840006, 233841005, 233842003, 233843008, 233846000, 233847009, 233885007, 282006, 285981000119103, 285991000119100, 30277009, 304914007, 307140009, 308065005, 311792005, 311793000, 311796008, 314207007, 32574007, 371068009, 371824008, 371864007, 371865008, 371866009, 371867000, 380001000004106, 394710008, 399211009, 401303003, 401314000, 418044006, 42531007, 428196007, 428752002, 429391004, 429731003, 43630006, 461000119108, 52035003, 54329005, 57054005, 58612006, 59063002, 62695002, 64627002, 65547006, 66189004, 698593009, 70211005, 703164000, 703165004, 703209002, 703210007, 703211006, 703212004, 703213009, 703251009, 703252002, 703253007, 703326006, 703328007, 703330009, 703360004, 70422006, 70998009, 710031008, 71023004, 7326005, 73795002, 73999000, 76593002, 79009004 |
|  | ICD-10 | I21.X, I22.X, I23.0, I23.1, I23.2, I23.3, I23.5, I23.6, I23.8, I24.1, I24.8, I25.10, I25.6, I25.9, I97.111, I97.191, I97.791, I25.2 |
| Congestive heart failure | ICD-9 | 402.01, 402.11, 402.91, 404.01, 404.03, 404.11, 404.13, 404.91, 404.93,398.91 |
|  | SNOMED | 15781000119107, 194779001, 194781004, 438367009, 5148006, 77737007, 3105008,194767001 |
|  | ICD-10 | 142.9X, 143.X, 150.X, 425.4X, 425.5X, 425.7X, 425.8X, 425.9X, 426.6X, 428.X, I11.0, I13.0, I13.2, I25.5, I42.0, I42.5X, I42.6X, I42.7X, I42.8X, P29.0, I09.9 |
| Peripheral vascular disease | ICD-9 | 437.3, 440.X, 441.X, 443.X, 447.1, 447.9, 459.39, 459.89, 557.1, 557.9, V43.4,093.0 |
|  | SNOMED | 153811000119105, 195313002, 233958001, 449821000124101, 449831000124103,399957001 |
|  | ICD-10 | I70.X, I71.X, I73.8, I73.9, I77.1, I77.9, I79.0, I79.2, I87.399, I99.8, K55.1, K55.8, K55.9, Z95.9, I73.1 |
| Cerebrovascular disease | ICD-9 | 377.72, 430.X-438.X, V12.54,362.34 |
|  | SNOMED | 102831000119104, 103761000119107, 106241000119108, 108691000119102, 111297002, 111298007, 116288000, 118951000119103, 118971000119107, 125081000119106, 133981000119106, 133991000119109, 137991000119103, 140221000119109, 140281000119108, 140701000119108, 140711000119106, 140911000119109, 140921000119102, 141821000119104, 141831000119101, 145741000119101, 148871000119109, 149821000119103, 151161000119102, 15258001, 15967181000119108, 161511000, 18761000119108, 192813004, 195185009, 195186005, 195189003, 195190007, 195199008, 195200006, 195201005, 195206000, 195209007, 195211003, 195212005, 195213000, 195230003, 195231004, 195232006, 195233001, 195234007, 195236009, 195239002, 195243003, 20059004, 204501003, 21290001000004104, 230523009, 230690007, 230692004, 230694003, 230695002, 230696001, 230698000, 230699008, 230700009, 230701008, 230702001, 230703006, 230704000, 230706003, 230708002, 230713003, 230715005, 230716006, 230717002, 230738008, 23671000119107, 25133001, 26021000119107, 266257000, 275434003, 275526006, 276219001, 276220007, 276221006, 276222004, 281240008, 288723005, 293811000119100, 293831000119105, 29941000119105, 302904002, 302909007, 307363008, 307766002, 307767006, 308067002, 33331000119103, 34181000119102, 34191000119104, 34781003, 361000119103, 371040005, 371041009, 373606000, 390936003, 40161000119102, 413102000, 413758000, 41713005, 422504002, 425642008, 425882004, 426033005, 426788002, 426814001, 426983002, 427065003, 427296003, 427432001, 428668000, 429235008, 429993008, 430781000124102, 430831000124106, 430841000124101, 430947007, 430959006, 432051000124108, 432181000124104, 432191000124101, 432504007, 433183000, 433891000124100, 433911000124103, 433931000124109, 433941000124104, 433951000124102, 433961000124100, 433971000124107, 434141000124103, 434151000124101, 434951000124104, 434961000124102, 434991000124105, 436041000124107, 440140008, 441526008, 441630004, 441735003, 441887006, 441894009, 441960006, 441991000, 442024001, 442097001, 442181008, 442212003, 442668000, 442676003, 442733008, 443929000, 444172003, 444657001, 46421000119102, 48601000119107, 5571000124103, 57981008, 62914000, 64009001, 672521000119108, 672561000119103, 674161000119102, 674361000119104, 674401000119108, 690051000119100, 690071000119109, 69533002, 699429007, 703163006, 703205008, 703207000, 703208005, 705128004, 705130002, 710575003, 78569004, 87551000119101, 87555007, 90921000119104, 91601000119109, 92341000119107, 95457000, 95460007, 97531000119106, 9901000119100, 99051000119101, 99451000119105, 441759008 |
|  | ICD-10 | G45.X, G46.X, H47.649, I60.X, I61.X, I62.X, I63.X, I64.X, I65.X, I66.X, I67.X, I68.X, I69.X, Z86.73, H34.0 |
| Dementia | ICD-9 | 290.X, 331.19, 331.2, 294.1 |
|  | SNOMED | 10349009, 106021000119105, 14070001, 230285003, 230287006, 25772007, 420614009, 421023003, 421529006, 56267009, 703544004, 70936005, 713488003, 713844000, 281004 |
|  | ICD-10 | F00.X, F01.X, F02.X, F03.X, G30.X, G31.09, G31.1, G71.2, F05.1 |
| Chronic obstructive pulmonary disease | ICD-9 | 415.2, 416.9, 490.X-505.X, 506.4, 508.1, 508.8, 508.9, 514, 515,416.8 |
|  | SNOMED | 106001000119101, 10692761000119107, 11211003, 11641008, 12428000, 125294002, 125295001, 13151001, 135836000, 13645005, 16003001, 16846004, 1751000119100, 185086009, 195949008, 195951007, 195957006, 195958001, 195959009, 195963002, 195967001, 195977004, 196001008, 196026004, 233672007, 233675009, 233677001, 233678006, 233679003, 233681001, 233683003, 233685005, 233686006, 233688007, 233935004, 23958009, 266355005, 266356006, 266361008, 266364000, 276637009, 281239006, 285381006, 293241000119100, 30352005, 304527002, 313296004, 313297008, 313299006, 31387002, 31886003, 31898008, 34015007, 370218001, 370219009, 370220003, 370221004, 389145006, 390921001, 40100001, 401000119107, 401193004, 404808000, 405944004, 407674008, 409663006, 423889005, 424199006, 424643009, 425969006, 426656000, 426979002, 427295004, 427354000, 427603009, 427679007, 442025000, 4981000, 5281000124103, 55570000, 56968009, 57546000, 57607007, 57686001, 59327009, 59786004, 63088003, 66110007, 68328006, 707412000, 707413005, 707445000, 707446004, 707447008, 70756004, 708030004, 85761009, 86680006, 87433001, 901000119100, 91340006, 92807009, 93432008, |
|  | ICD-10 | I27.9, J27.82, J40.X-J47.X, J60.X-J67.X, J68.4, J70.1, J70.3, J70.4, J70.9, J81.1, J84.10, P25.0, Q32.4,I27.8 |
| Rheumatic disease | ICD-9 | 710.0X-710.4X, 714.0X-714.2X, 714.8, 725.X, 446.5 |
|  | SNOMED | 1212005, 193248005, 193250002, 193252005, 196136009, 202768008, 234529004, 238935002, 238936001, 239898008, 239899000, 239901009, 240120003, 281357005, 281358000, 31384009, 396230008, 402358003, 402425006, 715401008, 86365006, 86365006, 95415006 |
|  | ICD-10 | M05.X, M06.X, M32.X, M33.X, M34.X, M35.1, M35.3, M36.0, M31.5 |
| Peptic ulcer disease | ICD-9 | 531.X-534.X |
|  | SNOMED | 111353003, 12274003, 12355008, 12847006, 15115006, 15902003, 16694003, 17067009, 17593008, 18367003, 2066005, 22157005, 23812009, 24001002, 26221006, 27281001, 28945005, 307233002, 308882008, 34021006, 35560008, 36975000, 41986000, 42698006, 43406003, 45640006, 46523000, 46708007, 47064007, 48658001, 48974009, 49232000, 50663005, 51847008, 53337006, 53877005, 54798007, 55617001, 55746001, 56461008, 57246001, 58711008, 59356009, 59515005, 61300005, 62341002, 62366003, 62838000, 63954007, 64094003, 64121000, 64398008, 66673003, 66767006, 70418001, 72408002, 74341002, 76078009, 76181002, 77661009, 81142005, 81387001, 81518000, 84124004, 85787009, 85859006, 86258000, 86895006, 87756006, 87796008, 89469000, 89748001, 90257004, 90489006 |
|  | ICD-10 | ,K25.X-K28.X |
| Mild liver disease | ICD-9 | 070.20, 070.23, 070.32, 070.33, 070.44, 070.54, 070.6, 070.9, 570.X, 571.X, 572.2, 573.3, 573.4, 573.8, 573.9, 582.8, V42.7,070.22 |
|  | SNOMED | 103611000119102, 10807061000119103, 1082611000119101, 1085021000119106, 1092801000119102, 109819003, 111371005, 1116000, 123604002, 123605001, 123606000, 12368000, 123716002, 123717006, 15230009, 153091000119109, 15999000, 1761006, 17890003, 1861000, 186639003, 192811002, 197279005, 197284004, 197286002, 197291001, 197293003, 197294009, 197296006, 197299004, 197301006, 197303009, 197305002, 197310003, 197315008, 197321007, 197359004, 197362001, 197364000, 199117000, 199118005, 19943007, 2043009, 230800004, 235856003, 235859005, 235869004, 235871004, 235875008, 235878005, 235880004, 235881000, 235895002, 235896001, 235897005, 235899008, 235901004, 235902006, 243978007, 266468003, 266469006, 266470007, 266471006, 271440004, 27156006, 276552008, 28698006, 307757001, 31712002, 328383001, 33144001, 347891000119103, 36631002, 370889009, 371139006, 37688005, 38662009, 41309000, 413438002, 41889008, 419728003, 420054005, 424340000, 425413006, 427022004, 43904005, 447058001, 45256007, 50167007, 50325005, 536002, 57339008, 58008004, 59229005, 6183001, 61977001, 62484002, 703866000, 713181003, 713370005, 713529007, 713966008, 716203000, 722866000, 723829000, 725416005, 725938001, 725939009, 725940006, 735733008, 737202006, 76301009, 76783007, 78208005, 79720007, 831000119103, 831000119103, 86454000, 89580002, 95557003, 9953008 |
|  | ICD-10 | B18.X, B19.11, K70.0X-K70.3X, K71.3X-K71.5X, K71.7, K72.91, K73.X, K74.X, K76.0, K76.2X-K76.4X, K76.8, K76.9, Z94.4, K70.9 |
| Diabetes without chronic complication | ICD-9 | 250.0X-250.3X, 250.9, 250.8 |
|  | SNOMED | 111231000119109, 111552007, 123763000, 137931000119102, 140401000119104, 140411000119101, 140521000119107, 1481000119100, 190330002, 190331003, 190368000, 190372001, 190388001, 190389009, 199229001, 199230006, 201250006, 23045005, 237599002, 237601000, 237604008, 237611007, 237613005, 237618001, 237619009, 237651005, 24203005, 28032008, 31321000119102, 313435000, 313436004, 314771006, 314893005, 314903002, 314904008, 359642000, 408539000, 420270002, 420868002, 421075007, 422228004, 426875007, 42954008, 44054006, 441628001, 443694000, 46635009, 530558861000132104, 59079001, 609561005, 609562003, 609565001, 609568004, 609569007, 609570008, 609571007, 609572000, 609573005, 609574004, 609575003, 609576002, 609577006, 609578001, 701000119103, 703136005, 703138006, 70694009, 709147009, 716362006, 719216001, 720519003, 722454003, 724136006, 724876003, 73211009, 75682002, 761000119102, 791000119109, 81531005, 8801005, 91352004, 9859006 |
|  | ICD-10 | E10.1, E10.6, E10.8, E10.9, E11.0, E11.1, E11.6, E11.8, E11.9, E12.0, E12.1, E12.6, E12.8, E12.9, E13.0, E13.1, E13.6, E13.8, E13.9, E14.0, E14.1, E14.6, E14.8, E14.9, E10.0 |
| Diabetes with chronic complication | ICD-9 | 250.4X-250.7X |
|  | SNOMED | 102781000119107, 103981000119101, 104941000119109, 104951000119106, 104961000119108, 109171000119104, 110996009, 126531000119109, 126591000119108, 127013003, 127014009, 127991000119101, 128001000119105, 138881000119106, 138891000119109, 138901000119108, 138911000119106, 138921000119104, 138941000119105, 140101000119109, 140111000119107, 140121000119100, 1491000119102, 1501000119109, 1551000119108, 18521000119106, 193349004, 193350004, 197605007, 232020009, 232021008, 232022001, 232023006, 236499007, 236500003, 243421000119104, 25412000, 28331000119107, 309426007, 310387003, 311366001, 311782002, 31211000119101, 312903003, 312904009, 312905005, 312906006, 312907002, 312908007, 312909004, 312910009, 312912001, 314010006, 314011005, 314014002, 314015001, 314902007, 368521000119107, 368711000119106, 368721000119104, 368741000119105, 38046004, 39058009, 390834004, 399862001, 399863006, 399864000, 399865004, 399866003, 399868002, 399869005, 399870006, 399871005, 399872003, 399873008, 399874002, 399875001, 399876000, 399877009, 401110002, 408409007, 408410002, 408411003, 408412005, 408413000, 408414006, 414894003, 414910007, 417677008, 420279001, 420486006, 420514000, 420715001, 420756003, 420789003, 420918009, 421305000, 421365002, 421779007, 421893009, 421895002, 421920002, 421986006, 422034002, 422166005, 425455002, 426907004, 427027005, 427571000, 43959009, 445170001, 4855003, 59276001, 60961000119107, 60971000119101, 60991000119100, 63510008, 711000119100, 712882000, 71701000119105, 71721000119101, 71791000119104, 721000119107, 731000119105, 82541000119100, 82571000119107, 82581000119105, 90721000119101, 90731000119103, 90751000119109, 90761000119106, 90771000119100, 90791000119104, 96441000119101, 97331000119101, 97341000119105,90741000119107 |
|  | ICD-10 | E10.2X-E10.5X, E11.2X-E11.5X, E11.7, E12.2X-E12.5X, E12.7, E13.2X-E13.5X, E13.7, E14.2X-E14.5X, E14.7, J44.X-J47.X, J60.X-J67.X, E10.7 |
| Hemiplegia or paraplegia | ICD-9 | 342.X, 343.X, 344.0X-344.6X, 344.9, 438.20,334.1 |
|  | SNOMED | 103761000119107, 140281000119108, 140701000119108, 140711000119106, 230702001, 24654003, 361000119103, 425491009, 432181000124104, 432191000124101, 441991000, 442024001, 442668000, 442676003, 442733008, 48601000119107, 54364001 |
|  | ICD-10 | G11.4, G80.1, G80.2, G81.X, G82.X, G83.0X-G83.4X, G83.9, I69.259, I69.359, G04.1 |
| Renal disease | ICD-9 | 403.11, 403.91, 404.02, 404.03, 404.12, 404.13, 404.92, 404.93, 582.X, 583.0X-583.7X, 585.X, 586.X, 588.0, V42.0, V45.1, V56.X,403.01 |
|  | SNOMED | 104931000119100, 111411000119103, 117681000119102, 120261000119101, 127991000119101, 128001000119105, 129151000119102, 129161000119100, 129171000119106, 129181000119109, 140101000119109, 140111000119107, 140121000119100, 14973001, 153851000119106, 153891000119101, 16218721000119107, 194780003, 236434000, 236435004, 236436003, 28119000, 284991000119104, 285011000119108, 285001000119105, 285081000119102, 285101000119109, 285841000119104, 285851000119102, 285861000119100, 285871000119106, 285881000119109, 285911000119109, 285921000119102, 286371000119107, 38481006, 428937001, 428982002, 429075005, 431857002, 433144002, 433146000, 434431000124103, 443143006, 443596009, 46177005, 473392002, 49220004, 57684003, 66052004, 66610008, 691401000119104, 691411000119101, 691421000119108, 698591006, 698810000, 700378005, 700379002, 704667004, 707324008, 711000119100, 712487000, 713696000, 714152005, 714153000, 71701000119105, 721000119107, 731000119105, 78544004, 8501000119104, 86234004, 90721000119101, 90731000119103, 90741000119107, 90751000119109, 90761000119106, 90771000119100, 90791000119104, 96441000119101, 96701000119107, 96711000119105, 96721000119103, 96731000119100, 96741000119109, 96751000119106 |
|  | ICD-10 | I13.1, N03.4X-N03.7X, N05.2X-N05.7X, N18.X, N19.X, N25.0, Z49.0X-Z49.2X, Z94.0, Z99.2, I12.0 |
| Any malignancy, including lymphoma and leukemia, except malignant neoplasm of skin | ICD-9 | 140.X-159.X, 161.X-172.X, 174.X-195.X, 200.X-208.X,238.6 |
|  | SNOMED | 109838007, 109839004, 109840002, 109841003, 109842005, 109843000, 109844006, 109962001, 109964000, 109965004, 109966003, 109967007, 109968002, 109969005, 109970006, 109971005, 109972003, 109975001, 109976000, 109977009, 109978004, 109979007, 109980005, 109988003, 109991003, 110000005, 110002002, 110004001, 110005000, 110006004, 110007008, 116711000119103, 116741000119104, 116811000119106, 116821000119104, 117061000119101, 117091000119108, 117111000119100, 118599009, 118600007, 118601006, 118602004, 118605002, 118606001, 118607005, 118608000, 118609008, 118610003, 118611004, 118612006, 118613001, 118614007, 118615008, 118617000, 118618005, 122881000119107, 122901000119109, 122951000119108, 122961000119105, 122981000119101, 12301000132103, 12311000132101, 123781000119107, 127070008, 127220001, 127225006, 127961000119108, 13048006, 133751000119102, 1701000119104, 184881000119106, 187757001, 187767006, 187769009, 187773007, 187776004, 187777008, 187786003, 187822008, 188269007, 188487008, 188489006, 188492005, 188493000, 188498009, 188500005, 188501009, 188502002, 188503007, 188504001, 188505000, 188506004, 188507008, 188510001, 188511002, 188512009, 188513004, 188514005, 188515006, 188516007, 188517003, 188524002, 188526000, 188529007, 188531003, 188534006, 188536008, 188537004, 188538009, 188541000, 188544008, 188547001, 188548006, 188551004, 188554007, 188558005, 188559002, 188562004, 188565002, 188566001, 188567005, 188568000, 188569008, 188570009, 188572001, 188575004, 188576003, 188577007, 188578002, 188579005, 188580008, 188582000, 188585003, 188586002, 188587006, 188589009, 188590000, 188591001, 188592008, 188593003, 188609000, 188612002, 188613007, 188627002, 188630009, 188631008, 188632001, 188633006, 188634000, 188635004, 188637007, 188640007, 188641006, 188642004, 188645002, 188648000, 188649008, 188662007, 188660004, 188663002, 188664008, 188665009, 188666005, 188667001, 188668006, 188669003, 188672005, 188674006, 188675007, 188676008, 188679001, 188718006, 188725004, 188726003, 188728002, 188729005, 188732008, 188736006, 188737002, 188738007, 188741003, 188744006, 188745007, 188746008, 188748009, 188754005, 188768003, 188770007, 190030009, 203436008, 232075002, 236512004, 236513009, 240531002, 253018005, 254582000, 254586002, 254601002, 254792006, 254994000, 255081007, 255101006, 255102004, 269475001, 269476000, 269533000, 269544008, 274902006, 274905008, 276811008, 276815004, 276822007, 276836002, 277473004, 277474005, 277545003, 277549009, 277550009, 277551008, 277567002, 277568007, 277569004, 277570003, 277571004, 277572006, 277573001, 277574007, 277575008, 277587001, 277589003, 277601005, 277602003, 277604002, 277609007, 277610002, 277611003, 277612005, 277613000, 277614006, 277615007, 277616008, 277617004, 277618009, 277619001, 277622004, 277623009, 277624003, 277625002, 277626001, 277627005, 277628000, 277629008, 277632006, 277637000, 277641001, 277642008, 277643003, 277651000, 277653002, 277664004, 278024000, 278051002, 278052009, 278189009, 278453007, 285312008, 285769009, 285776004, 285839005, 301756000, 302841002, 302842009, 302845006, 302848008, 302855005, 302856006, 303017006, 30305500, 303056000, 303057009, 307341004, 307592006, 307617006, 307622006, 307623001, 307624007, 307625008, 307633009, 307634003, 307635002, 307636001, 307637005, 307646004, 307647008, 307649006, 307650006, 308121000, 312111009, 312112002, 312113007, 312114001, 312115000, 314963000, 314965007, 314966008, 314997007, 314998002, 315058005, 359631009, 359640008, 359648001, 363350007, 363351006, 363406005, 363407001, 363408006, 363409003, 363410008, 363412000, 363413005, 363414004, 363491008, 363495004, 363510005, 369448007, 369449004, 369450004, 369451000, 369452007, 369453002, 369454008, 369455009, 369456005, 369457001, 369458006, 369459003, 369460008, 369461007, 371012000, 371134001, 371977004, 373168002, 3854002, 397008008, 397009000, 397011009, 400122007, 402881008, 402882001, 404106004, 404107008, 404108003, 404109006, 404110001, 404111002, 404112009, 404113004, 404114005, 404115006, 404116007, 404117003, 404118008, 404119000, 404120006, 404121005, 404122003, 404123008, 404124002, 404128004, 404129007, 404130002, 404131003, 404132005, 404134006, 404136008, 404137004, 404138009, 404139001, 404140004, 404141000, 404142007, 404143002, 404144008, 404145009, 404147001, 404148006, 404149003, 404150003, 404151004, 404152006, 404153001, 404154007, 404155008, 404157000, 404169008, 404172001, 408645001, 408646000, 413389003, 413441006, 413442004, 413446001, 413656006, 413842007, 413843002, 413847001, 414780005, 414785000, 415112005, 415287001, 420302007, 420519005, 420524008, 420788006, 421246008, 421283008, 422052002, 422853008, 425178004, 425688002, 425749006, 425869007, 425941003, 426071002, 426124006, 426217000, 426248008, 426336007, 426370008, 426642002, 426885008, 427056005, 427141003, 427642009, 427658007, 430338009, 433511000124108, 433541000124107, 433551000124109, 433561000124106, 433571000124104, 433581000124101, 436321000124102, 436331000124104, 436341000124109, 436351000124106, 441559006, 441962003, 442537007, 443487006, 444597005, 444910004, 444911000, 445227008, 445269007, 445448008, 447100004, 447109003, 447656001, 447658000, 447766003, 447805007, 447806008, 447989004, 448212009, 448372003, 448213004, 448217003, 448220006, 448231003, 448254007, 448269008, 448317000, 448319002, 448354009, 448371005, 448376000, 448384001, 448386004, 448387008, 448447004, 448465000, 448468003, 448553002, 448555009, 448560008, 448561007, 448607004, 448609001, 448663003, 448666006, 448672006, 448709005, 448738008, 448774004, 448865007, 448867004, 448994001, 448995000, 449058008, 449059000, 449063007, 449065000, 449074003, 449075002, 449108003, 449173006, 449176003, 449177007, 449216004, 449217008, 449218003, 449219006, 449220000, 449221001, 449222008, 449292003, 449307001, 449318001, 449386007, 449418000, 449419008, 450521000124109, 61291000119103, 61301000119102, 61311000119104, 61321000119106, 681601000119101, 698646006, 699818003, 702446006, 702786004, 702977001, 703387000, 709471005, 713325002, 713483007, 713516007, 713718006, 713897006, 714251006, 714463003, 715664005, 715950008, 716648006, 716654007, 716788007, 718200007, 721305008, 721306009, 721308005, 721310007, 721313009, 721314003, 721555001, 721695008, 721696009, 721699002, 721716004, 721762007, 722795004, 722953004, 722954005, 723889003, 724644005, 724645006, 724647003, 724648008, 724649000, 724650000, 725390002, 725437002, 726721002, 733598001, 733627006, 733834006, 734066005, 737058005, 739301006, 84811000119107, 84831000119102, 91854005, 91855006, 91856007, 91857003, 91858008, 91860005, 91861009, 92508006, 92509003, 92510008, 92511007, 92512000, 92513005, 92514004, 92515003, 92516002, 92811003, 92812005, 92813000, 92814006, 92817004, 92818009, 93133006, 93134000, 93135004, 93136003, 93137007, 93138002, 93139005, 93140007, 93141006, 93142004, 93143009, 93144003, 93145002, 93146001, 93147005, 93148000, 93149008, 93150008, 93151007, 93152000, 93169003, 93182006, 93183001, 93184007, 93185008, 93186009, 93187000, 93188005, 93189002, 93190006, 93191005, 93192003, 93193008, 93194002, 93195001, 93196000, 93197009, 93198004, 93199007, 93200005, 93201009, 93202002, 93203007, 93204001, 93205000, 93206004, 93207008, 93208003, 93450001, 93451002, 93487009, 93488004, 93489007, 93492006, 93493001, 93494007, 93495008, 93496009, 93497000, 93498005, 93500006, 93501005, 93505001, 93506000, 93507009, 93509007, 93510002, 93514006, 93515007, 93516008, 93518009, 93519001, 93520007, 93521006, 93522004, 93523009, 93524003, 93525002, 93526001, 93527005, 93528000, 93530003, 93531004, 93532006, 93533001, 93534007, 93536009, 93537000, 93541001, 93542008, 93543003, 93545005, 93546006, 93547002, 93548007, 93549004, 93550004, 93551000, 93552007, 93554008, 93555009, 93683002, 93761005, 93771007, 93772000, 93826009, 93870000, 93899005, 93900000, 93901001, 93902008, 93903003, 93904009, 93905005, 93906006, 93907002, 93908007, 93909004, 93910009, 93911008, 93912001, 93913006, 93980002, 93984006, 94006002, 94071006, 94072004, 94105000, 94148006, 94686001, 94687005, 94688000, 94690004, 94707004, 94708009, 94709001, 94710006, 94711005, 94712003, 94714002, 94715001, 94716000, 94718004, 94719007, 95186006, 95187002, 95188007, 95192000, 95193005, 95194004, 95209008, 95210003, 95214007, 95224004, 95225003, 95226002, 95230004, 95231000, 95260009, 95261008, 95263006, 95264000, 96281000119107, 96981000119102, 98981000119103,449053004 |
|  | ICD-10 | C30.X-C34.X, C37.X-C41.X, C43.X, C45.X-C58.X, C60.X-C76.X, C81.X-C85.X, C88.X, C90.X-X97.X, C00.X-C26.X |
| Moderate or severe liver disease | ICD-9 | 456.0X-456.2X, 572.2X-572.8X,570 |
|  | SNOMED | 1082621000119108, 11350001, 13923006, 153091000119109, 197268000, 197269008, 197270009, 197356006, 213230009, 235881000, 235884008, 235886005, 24807004, 26485002, 302126000, 37292007, 405542005, 413438002, 435091000124105, 435101000124104, 51292008, 53104002, 55294001, 59927004, 61250001, 62216007, 65617004, 707167006, 708248004, 720461006, 722864002, 724361001, 81675001, 87248009, 89835009,17709002 |
|  | ICD-10 | I85.9, I86.4, I98.2, K70.4, K71.1, K72.00, K72.1, K72.9, K76.5, K76.6, K76.7, I85.0 |
| Metastatic solid tumor | ICD-9 | 196.X-199.X |
|  | SNOMED | 116821000119104, 1691000119104, 236512004, 239898008, 277664004, 285610008, 285611007, 285612000, 285613005, 314997007, 314998002, 369448007, 369449004, 369450004, 369451000, 369452007, 369453002, 369454008, 369455009, 369456005, 369457001, 369458006, 369459003, 369460008, 369461007, 404122003, 422282000, 433511000124108, 433521000124100, 433531000124102, 433541000124107, 433551000124109, 433561000124106, 433571000124104, 436321000124102, 436331000124104, 436341000124109, 448922007, 608874000, 713572001, 94179005, 94235004, 94260004, 94271003, 94272005, 94328005, 94349006, 94365007, 94381002, 94418000, 94419008, 94420002, 94421003, 94422005, 94423000, 94424006, 94425007, 94426008, 94427004, 94428009, 94429001, 94430006, 94431005, 94432003, 94509004, 94513006, 94538001, 94604000, 94643001, 96981000119102,116811000119106 |
|  | ICD-10 | C77.X-C80.X |
| AIDS/HIV | ICD-9 | 042.X-044.X |
|  | SNOMED | 10746341000119109, 111880001, 186706006, 186707002, 186708007, 186709004, 186717007, 186718002, 186719005, 186721000, 186723002, 186725009, 186726005, 230180003, 230201009, 230598008, 235009000, 235726002, 240103002, 276666007, 315019000, 359791000, 397763006, 398329009, 402915006, 402916007, 405631006, 40780007, 421508002, 421510000, 421529006, 421571007, 421597001, 421660003, 421666009, 421671002, 421695000, 421706001, 421708000, 421710003, 421766003, 421827003, 421851008, 421874007, 421883002, 421929001, 421983003, 421998001, 422003001, 422012004, 422074008, 422089004, 422127002, 422136003, 422177004, 422189002, 422194002, 422282000, 422337001, 442134007, 442537007, 445945000, 48794007, 52079000, 5810003, 62246005, 62479008, 697904001, 697965002, 700053002, 713260006, 713275003, 713278001, 713297001, 713298006, 713299003, 713300006, 713316008, 713318009, 713320007, 713325002, 713339002, 713340000, 713341001, 713342008, 713349004, 713444005, 713445006, 713446007, 713483007, 713484001, 713487008, 713488003, 713489006, 713490002, 713491003, 713497004, 713503007, 713504001, 713505000, 713506004, 713507008, 713508003, 713510001, 713511002, 713523008, 713526000, 713527009, 713530002, 713531003, 713532005, 713533000, 713543002, 713544008, 713545009, 713546005, 713570009, 713571008, 713572001, 713695001, 713696000, 713718006, 713722001, 713729005, 713730000, 713731001, 713732008, 713733003, 713734009, 713844000, 713845004, 713880000, 713881001, 713887002, 713897006, 713964006, 713967004, 714083007, 714464009, 719522009, 721166000, 722557007, 72621000119104, 733834006, 733835007, 735521001, 735522008, 735523003, 735524009, 735525005, 735526006, 735527002, 735528007, 76981000119106, 76991000119109, 77070006, 80191000119101, 81000119104, 86406008, 87117006,442134007 |
|  | ICD-10 | B24.X, B20.X-B22.X |

**Supplemental Table 10**. List of codes used to define mental health conditions

| **Mental Health Disorder** | **Code Type** | **Code(s)** |
| --- | --- | --- |
| Anxiety Disorders | ICD-9 | 293.84, 300.10, 300.20, 300.21, 300.22, 300.3, 300.0X, 308.X |
|  | SNOMED | 48694002, 197480006, 21897009, 192404005, 21897009, 52910006, 160332003, 154882009, 268752000, 191706008, 192401002, 192459009, 191720001, 111487009, 16265701000119107, 17496003, 192398000, 192400001, 192405006, 197480006, 154884005, 191703000, 192192006, 65673007, 126943008, 192393009, 192397005, 192399008, 192403004, 268714001, 436001000124105, 69479009, 371631005, 386810004, 25501002, 52039009, 191736004, 71478004, 1376001, 191739006, 192411009, 192406007, 192410005, 192394003, 414371008, 111490003, 111491004, 11941006, 1380006, 1816003, 191722009, 191723004, 19766004, 22230001, 24781009, 30059008, 3158007, 31781004, 32388005, 34116005, 35607004, 38328002, 43150009, 4932002, 49564006, 50983008, 53956006, 5509004, 56576003, 59923000, 61212007, 61569007, 63701002, 63909006, 64060000, 65064003, 70691001, 72861004, 74010007, 76812003, 76868007, 8185002, 82415003, 82494000, 82738004, 83631006, 87798009, 89948007, 154885006 |
|  | ICD-10 | F06.4, F40.0X, F41.X, F42.X |
| Depression | ICD-9 | 296.92, 300.4, 309.1, 311, 296.2X, 296.3X, 296.5X |
|  | SNOMED | 25922000, 35489007, 320751009, 36923009, 370143000, 42925002, 69392006, 63778009, 87512008, 79298009, 48589009, 832007, 15639000, 73867007, 75084000, 430852001, 77911002, 20250007, 76441001, 19527009, 14183003, 300706003, 231499006, 321717001, 63412003, 30605009, 42810003, 70747007, 726772006, 320751009, 36923009, 370143000, 10811121000119102, 10811161000119107, 42925002, 69392006, 63778009, 87512008, 79298009, 16265951000119109, 720455008, 720454007, 832007, 15639000, 16266831000119100, 719592004, 720453001, 450714000, 73867007, 33736005, 60099002, 75084000, 251000119105, 430852001, 77911002, 20250007, 76441001, 16266991000119108, 19527009, 191606003, 191601008, 191602001, 191604000, 2506003, 19694002, 83176005, 38451003, 67711008, 85080004, 3109008, 36170009, 78667006, 2506003, 19694002, 83176005, 38451003, 67711008, 85080004, 3109008, 36170009, 191659001, 192080009, 35489007, 40379007, 191610000, 40379007, 191610000, 18818009, 191611001, 18818009, 191611001, 36474008, 281000119103, 36474008, 39809009, 191613003, 28475009, 33078009, 15193003, 39809009, 191613003, 28475009, 33078009, 15193003, 68019004, 33135002, 46244001, 191615005, 40568001, 38694004, 274948002, 38694004, 2618002, 191616006, 66344007, 319768000, 71336009, 268621008, 2618002, 720451004, 720452006, 66344007, 319768000, 71336009, 268621008 |
|  | ICD-10 | F31.4, F31.5, F31.75, F31.76, F31.77, F31.78, F31.81, F32.0, F32.1, F32.2, F32.3, F32.4, F32.5, F32.9, F32.A, F33, F34.1, F31.3X, F31.6X, F33.X |
| ADD/ADHD | ICD-9 | 314.X |
|  | SNOMED | 406506008, 35253001, 35253001, 192127007, 31177006 |
|  | ICD-10 | F90.X |
| Bipolar Disorder | ICD-9 | 296.X |
|  | SNOMED | 191627008, 191618007, 192362008, 191634005, 191629006, 191630001, 191632009, 191625000, 191620005, 191621009, 191623007, 13746004, 41836007, 5703000, 85248005, 371596008, 29929003, 21900002, 75752004, 87203005, 31446002, 17782008, 55516002, 73471000, 65042007, 83225003, 48937005, 16295005, 43568002, 22407005, 34315001, 30687003, 51637008, 1196001, 49468007, 22121000, 49512000, 53607008, 68569003, 30935000, 63249007, 45479006, 13313007, 41552001, 71294008, 74686005, 71984005, 43769008, 191636007, 191643001, 191638008, 191639000, 191641004, 16506000, 111485001, 36583000, 35481005, 79584002, 28884001, 35846004, 66631006, 82998009, 40926005, 371600003, 4441000, 70546001, 26530004, 53049002, 371599001, 14495005, 371604007, 30520009, 19300006, 20960007, 81319007, 12969000, 67002003, 35722002, 59617007, 54761006, 26203008, 61403008, 28663008, 78640000, 33380008, 162004, 10981006, 64731001, 10875004, 46229002, 191627008, 191618007, 192362008, 191634005, 191629006, 191630001, 191632009, 191625000, 191620005, 191621009, 191623007, 13746004, 41836007, 5703000, 85248005, 371596008, 87203005, 31446002, 17782008, 55516002, 73471000, 65042007, 83225003, 48937005, 16295005, 43568002, 22407005, 34315001, 30687003, 51637008, 1196001, 49468007, 22121000, 49512000, 53607008, 68569003, 30935000, 63249007, 45479006, 13313007, 41552001, 71294008, 74686005, 71984005, 43769008, 191636007, 191643001, 191638008, 191639000, 191641004, 16506000, 111485001, 36583000, 35481005, 79584002, 28884001, 35846004, 66631006, 82998009, 40926005, 133091000119105, 371600003, 4441000, 70546001, 26530004, 53049002, 371599001, 14495005, 371604007, 19300006, 20960007, 81319007, 261000119107, 61403008, 23741000119105, 28663008, 78640000, 33380008, 162004, 271000119101, 10981006, 64731001, 10875004, 46229002, 81319007 |
|  | ICD-10 | F31.X |
| Schizophrenic/Psychotic Disorders | ICD-9 | 295.X, 297.X, 298.X |
|  | SNOMED | 58214004, 191527001, 191567000, 69322001 |
|  | ICD-10 | F20.X, F22.X, F23.X, F24.X, F28.X, F29.X |
| PTSD | ICD-9 | 309.81 |
|  | SNOMED | 47505003, 313182004, 443919007, 318784009, 192042008 |
|  | ICD-10 | F43.1X |
| Other | ICD-9 | 301.X, 307.X, 308.X, 309.0X, 309.1X, 309.2X, 309.3X, 309.4X, 312.X, 313.X |
|  | SNOMED | 72366004, 439960005, 33449004, 55341008, 20010003, 80711002, 698700004, 20010003, 191765005, 4306003 |
|  | ICD-10 | F30.X, F39.X, F43, F43.0, F43.2, F43.8, F43.9, F44.X, F45.X, F48.X, F50.X, F53.X, F54.X, F60.X, F63.X, F65.X, F68.X, F69.X, F91.X, F93.X, F94.X, F95.X, F98.X, F99.X |

**Supplemental Table 11.** List of codes used to define chronic pain

| **Code Type** | **Codes** |
| --- | --- |
| ICD 9 | 053.12, 053.13, 53.12, 53.13, 260.6X, 274.X, 307.8X, 337.0, 337.1, 337.2X, 338.0, 338.2, 338.2X, 338.4, 339.X, 346.X, 349, 350.X, 352.1, 353.X, 354.X, 355.X, 356.X, 357.X, 377.X, 710-739.X, 780.96, 784.0, |
| ICD 10 | B02.22, B02.23, B02.29, D48.1X, E08.42, E09.42, E10.42, E11.42, E13.42, F45.4X, G43.X, G44.X, G50.X, G51.X, G52.X, G53.X, G54.0X, G54.1X, G54.2X, G54.3X, G54.4X, G54.5X, G54.6X, G54.8X, G54.9X, G56.0X, G56.4X, G57.X, G58.7, G58.9, G60.X, G62.X, G63.X, G64.X, G65.X, G89.0, G89.2, G89.2X, G89.4, G90.0X, G90.5X, G97.1, G99.0, H46.X, H47.X, M00.X, M01.X, M02.X, M05.X, M06.X, M07.X, M08.X, M1A.X, M10.X, M11.X, M12.X, M13.X, M14.X, M15.X, M16.X, M17.X, M18.X, M19.X, M20.X, M21.X, M22.X, M23.X, M24.X, M25.X, M30-M99.X, R26.2X, R26.4X, R29.898, R51.X, R52, S12.X, S13.X, S14.X, S22.X, S23.X, S24.X, S32.X, S33.X, S34.X, S39.012X, S39.023X, S39.092X, S83.0X, S83.1X, S83.2X, S83.3X, S83.4X, S83.5X, S83.6X, S83.8X, S83.9X, M43.06 |
| SNOMED | 100491000119103, 10181000119102, 102481003, 10713006, 1073681000119109, 1073691000119107, 1073701000119107, 1073711000119105, 1073721000119103, 1073731000119100, 1073741000119109, 1073751000119106, 1073761000119108, 1073771000119102, 1073781000119104, 1073791000119101, 1073801000119100, 1073811000119102, 1073821000119109, 1073831000119107, 1073861000119104, 1073871000119105, 1073881000119108, 1073891000119106, 108523005, 109771000119103, 11055151000119108, 111218008, 111220006, 111221005, 111237004, 111985007, 1121000119107, 115491000119105, 117216004, 11892641000119101, 121881000119102, 122061000119109, 122141000119109, 122161003, 123798002, 12396006, 124001000119104, 12400141000119103, 12400181000119108, 12400221000119100, 12400261000119105, 12400301000119102, 12400341000119100, 124041000119102, 124071000119109, 124161000119104, 124171000119105, 128079007, 129501000119107, 129511000119105, 1304004, 133731000119108, 134407002, 136791000119103, 137741002, 137808009, 138752005, 138835002, 13888000, 139311007, 141481000119102, 14150005, 143245009, 143441000119108, 143557001, 145611000119107, 148071003, 148454008, 154931007, 155046006, 155047002, 155048007, 155072002, 155090001, 15633361000119103, 156471009, 156472002, 156473007, 156474001, 156475000, 156476004, 156477008, 156478003, 156479006, 156480009, 156481008, 156483006, 156625009, 156626005, 156649007, 156727006, 15685921000119102, 15685961000119107, 15686001000119104, 15686281000119101, 15686321000119106, 15687201000119107, 15687321000119109, 15687841000119108, 15691161000119108, 15691721000119102, 15691761000119107, 15691801000119104, 15743521000119108, 15743561000119103, 15744441000119101, 15744481000119106, 15744961000119106, 15749801000119104, 15968741000119100, 16002671000119106, 16002871000119105, 16002911000119108, 160342001, 160413008, 16041671000119109, 161481007, 161567008, 162040008, 16206661000119108, 16208561000119106, 16208721000119109, 16580691000119107, 165841003, 16839401000119104, 170845006, 171239004, 1715006, 17205007, 18347007, 192028000, 192499007, 193027003, 193029000, 193030005, 193031009, 193032002, 193033007, 193034001, 193035000, 193036004, 193037008, 193039006, 193040008, 193041007, 193105005, 193119005, 193125009, 193157005, 193180002, 193184006, 193250002, 194493009, 194494003, 194502007, 194503002, 194508006, 1961000, 197834003, 198407008, 201763001, 201764007, 201765008, 201766009, 201767000, 201768005, 201769002, 201770001, 201771002, 201772009, 201773004, 201774005, 201775006, 201776007, 201777003, 201778008, 201779000, 201780002, 201781003, 201782005, 201783000, 201784006, 201785007, 201787004, 201790005, 201791009, 201796004, 201798003, 201799006, 201800005, 201808003, 201810001, 201811002, 201813004, 201819000, 201826000, 201829007, 201831003, 201832005, 201833000, 201834006, 201836008, 201837004, 201847001, 201849003, 201850003, 201851004, 201852006, 201854007, 201855008, 202674000, 202675004, 202676003, 202677007, 202678002, 202679005, 202680008, 202693003, 202694009, 202695005, 202673006, 202696006, 202737009, 20279300, 202794004, 203082005, 203138004, 203490000, 203492008, 203493003, 203729008, 203730003, 203732006, 203746006, 206796000, 207219003, 207621000, 2103002, 21430002, 22193007, 227588009, 22902007, 230462002, 230463007, 230464001, 230465000, 230468003, 230469006, 230470007, 230471006, 230481005, 23056005, 230575000, 23186000, 232284007, 235841007, 237067000, 23894005, 239791005, 239792003, 239793008, 239794002, 239795001, 239801005, 239862000, 24271000087103, 24281000087101, 24291000087104, 24301000087100, 24311000087103, 24331000087108, 24351000087104, 24361000087101, 247365004, 25343008, 254779008, 26002007, 26150009, 267699004, 267887009, 267888004, 267972003, 267984001, 268052008, 2700007, 273206005, 274665008, 274669002, 275902004, 278860009, 279032003, 279039007, 28, 287006005, 287007001, 287008006, 287009003, 287010008, 28736004, 287984007, 290531000119102, 290541000119106, 3, 3061000119102, 307177001, 308143008, 311804006, 313501000119105, 314097005, 316692001, 318871000119107, 318881000119105, 318941000119109, 318951000119106, 319031000119108, 319081000119109, 319111000119104, 319841000119107, 320281000119104, 320291000119101, 322837006, 322838001, 33262002, 33952002, 35074008, 363558001, 373621006, 37796009, 37895003, 389320000, 390055001, 392680008, 393605009, 393638000, 394707001, 396275006, 398057008, 40089004, 402431009, 40709005, 408276001, 408381007, 408662006, 408954001, 408955000, 408956004, 408957008, 40913006, 410795001, 410796000, 410797009, 416666007, 417291007, 41907006, 419568001, 420454001, 421107001, 4222000, 423279000, 423683008, 424699007, 425007008, 425365009, 425936006, 426135001, 426566004, 426628005, 427419006, 427770001, 429181000124108, 429192004, 430696004, 431061000124100, 431481001, 431601000124105, 431707004, 432615008, 433228003, 434011000124101, 436771000124102, 437931000124100, 441711008, 442194005, 442277000, 442521001, 443349002, 443539004, 443728000, 444009006, 444060007, 444746004, 445322004, 445479007, 445985006, 446043004, 4473006, 448701000124105, 45064008, 458071000124104, 458081000124101, 459911000124100, 459921000124108, 46960006, 48210000, 49605003, 50642008, 51049005, 51777006, 51881000119109, 54314008, 56097005, 57160007, 58156007, 59185006, 59292006, 608837004, 631000119102, 64309007, 674051000119103, 69896004, 699314009, 701602006, 702840008, 703182002, 711545001, 712537009, 7126001, 713911007, 714252004, 722981005, 722982003, 724429004, 725058003, 726678001, 734947007, 734989004, 7355002, 735599007, 735600005, 735644008, 735935009, 735936005, 736464002, 737305006, 737306007, 74391003, 745233009, 75822003, 75879005, 7607008, 762451005, 762452003, 762454002, 762589002, 762590006, 762591005, 762593008, 762594002, 762595001, 762596000, 762597009, 762598004, 762599007, 762600005, 762601009, 762602002, 762603007, 764528008, 764529000, 76462000, 764894008, 764895009, 764896005, 764897001, 764898006, 764899003, 764900008, 764901007, 764902000, 764903005, 764904004, 764905003, 764906002, 773908008, 77994009, 781206002, 782661001, 788891004, 79267007, 80843008, 81455003, 82300000, 82304009, 82423001, 83351003, 83793004, 86119004, 86219005, 86345004, 90378003, 90641006, 90860001, 95417003, 95653008, 95655001, 95656000, 95657009, 95658004, 96531000119109, 98611000119104, 98611000119104 |

**Supplemental Table 12.** List of codes used to define benzodiazepine prescription

| **Code Type** | **Codes** |
| --- | --- |
| NDC | 5515433270, 5570062230, 6725390009, 5515449790, 6725390010, 6725390011, 6725390050, 6725390110, 6725390111, 6725390150, 6725390210, 6725390211, 6725390250, 6725390310, 6725390350, 5515454510, 5515454990, 4999903200, 4999903230, 4999903260, 4999903290, 4999903930, 4999903960, 4999903990, 6807118843, 6807118846, 6807118849, 6807118881, 6807118882, 6807118883, 6807118886, 6807118889, 6807119293, 6807119296, 6807130492, 6807130493, 6807130496, 6807130499, 6516280903, 6516280906, 6516280950, 6516281003, 5515455530, 5515458850, 6729607211, 6729609581, 5515468800, 6729613032, 781106101, 781106105, 781106110, 781107701, 781107705, 781107710, 781107901, 781107905, 81107910,  6516281006, 6516281050, 6516281203, 6516281206, 6516281250, 6516281303, 6516281306, 6754400515, 6729613131, 6729613792, 6754441415, 6754441430, 6754441445, 6754441453, 6754441460, 6754441470, 7133500980, 7133500981, 7133500982, 7133500983, 7133500984, 7133500985, 7133500986, 7133500987, 7133500988, 6807119299, 6807119661, 6807119663, 6807119666, 6807119669, 5528992006, 5528992030, 5528992040, 5528992060, 781108901, 781108905, 5043609032, 6626701315, 6626701330, 6626701360, 6626701390, 6626701430, 6626701460, 6076081330, 3326150000, 3326150002, 3326150010, 3326150015, 3326150030, 3326150042, 3326150045, 3326150060, 3326150090, 3326159400, 3326159402, 3326159410,  3326159415, 3326159430, 3326159442, 4306360930, 4306360940, 4306360960, 4306360990,  4306382840, 6754441480, 6754441515, 6754441520, 6754441530, 6754441545, 6754441553,  6754441555, 6754441560, 3326159445, 3326159460, 3326159490, 3326174107, 3326174120,  3326174130, 3326174145, 3326174160, 6754441570, 6754441575, 6754441580, 6754441592,  6318744330, 6318744360, 6754400530, 6754400545, 6754400553, 6754400560, 6754400570,  6754400580, 6754412415, 6754412430, 6318744390, 6318744430, 6318744460, 6318744490,  6626701490, 6626701491, 6626721906, 6626721930, 6626756430, 6626756460, 6626756490,  6626776030, 6626776060, 6626776003, 6626776006, 6626782006, 3326174190, 7133507613,  7133507615, 7133507610, 7133507617, 7133507619, 7133507611, 7133507616, 7133507612,  6754412445, 6754412453, 6754412460, 6754412470, 6318791630, 6318791660, 6318791690,  6754429015, 6754429030, 6754429045, 6754429053, 6754429055, 6754429060, 6754429070,  6754429075, 6754429080, 6754429090, 6754434715, 6754434730, 6754434745, 6754434753,  6754434760, 6754434770, 6754434775, 5570031330, 5570031360, 6318797190, 7133504273,  5570062260, 6807144472, 6807144479, 179009330, 179009350, 7133507614, 4306306106,  4306308806, 228202710, 228202750, 228202796, 228202910, 228202950, 228202996,  6807144476, 7133506733, 7133506735, 7133506804, 7133506806, 7133506801, 7133506808,  7133506732, 7133506736, 228203110, 228203150, 228203196, 228203910, 228308306, 228308406, 228308606, 228308706, 4306336406, 4306336430, 4306336440, 4306336460, 4306336490, 4306336498, 4306336706, 4306336715, 4306336730, 4306336760, 4306336790, 4306336798, 4306337005, 4306337006, 4306337020, 7133506800, 7133506802, 7133506807, 7133506809, 7133506731, 7133506734, 7133506803, 7133506805, 4586542430, 4306337030, 228401911, 228402211, 228402411, 228402511, 4306337060, 4306337090, 4306337098, 5570031390, 7133504271, 5570042230, 5570042260, 5570042290, 5570049130, 5570049160, 5570049190, 6878899972, 6878899973, 6878899976, 6878899978, 6878899979, 6318796630,  6318796660, 6318796690, 6808464701, 6754434780, 6754434790, 378400105, 378400305, 378400505, 378400701, 7133500989, 6878897273, 6878897276, 6878897279, 6878894302, 6878894303, 6878894306, 6878894309, 4586542460, 4586552160, 4586565315, 4586565360, 4586569630, 4586569660, 4586569690, 6050513311, 615779339, 615779439, 615779539, 615799939, 615800039, 6318797130, 6318797160, 179009430, 179009450, 179196430, 179196450, 179196530, 179196550, 1263452500, 1263452540, 1263452571, 6050513313, 6050513315, 6050513316, 6050513318, 6050513321, 6050513323, 6050513325, 6050513326, 6050513328, 6050513331, 6050513333, 6050513335, 6050513336, 6050513338, 6050513341,  6050513343, 6050513345, 6050513346, 6050513348, 6050526340, 6050526341, 6050526343, 6050526346, 6050526348, 6050526350, 6050526351, 6050526353, 6050526356, 6050526358, 6050526360, 6050526361, 6050526363, 6050526366, 6050526368, 904585861, 904585961, 904586061, 5570060230, 6878863582, 6878863583, 6878863586, 6878863589, 7133507618, 6807144473, 7133508131, 7133508133, 7133508134, 7133508136, 7133508138, 7133504227, 7051800000, 6191937930, 6878896823, 6878896826, 6878896829, 7133508135, 5043646332, 5043646333, 5043646341, 7133502150, 7133502151, 7133502152, 7133502153, 7133502154, 7133502155, 7133502156, 6050526370, 6050526371, 6050526373, 6050526376, 6050526378,  7051809660, 7133501631, 7133501632, 6808467201, 4988411074, 4988411174, 4988421374, 4988421474, 1263452580, 1263452585, 1263452591, 1263452595, 1263452596, 1263460771, 6878868652, 6878868653, 6878868656, 7133508132, 6878869433, 6878869436, 7133501633, 7133501634, 7133501635, 7133501636, 7133501637, 7133501638, 7133501639, 6878863812, 6878863813, 7133506737, 7133504487, 7133504272, 7133504274, 7133502157, 7133502158, 7133502159, 6178672302, 7133502470, 7133502471, 7133502472, 7133502473, 7133502474, 5295996515, 5295996530, 5295996560, 5300203402, 5300203403, 7133502475, 7133502476, 7133502477, 7133502478, 7133502479, 7133502520, 7133502521, 7133502522, 7133502523,  3326100400, 3326100402, 3326100410, 3326100415, 3326100430, 3326100442, 3326100445, 3326100460, 3326100490, 3326100500, 3326100502, 3326100510, 3326100515, 3326100530, 6878863816, 6878863818, 6878863819, 6586245460, 6586245471, 6586245499, 6586245560, 6586245571, 5043646342, 5043646351, 5043646352, 5043646361, 5043646362, 5570060260, 6318722530, 6318722560, 6878869439, 5976200571, 5976200591, 5976200661, 5976200681, 5976237191, 5976237193, 5976237194, 3326100542, 3326100545, 3326100560, 3326100590, 6498039601, 6498039610, 6498039650, 6498039701, 6878868659, 6373964410, 6373966910, 6373967410, 6754462915, 5976237201, 5976237203, 5976237204, 5976237211, 5976237213,  5976237214, 5976237221, 5976237223, 6498039710, 6498039750, 6498039801, 6498039810, 6498039850, 6498039901, 6498039950, 7133504221, 7133504222, 6754462920, 6754462930, 6754462945, 6754462953, 6754462955, 6754462960, 6754462970, 6754462975, 6586245599, 6586245660, 6586245671, 6586245699, 6586245760, 6586245771, 6586245799, 7133504223, 7133504224, 7133504225, 7133504226, 7133504481, 7133504482, 7133504483, 7133504484, 7133504485, 5528952301, 5528952330, 5528952340, 5528952360, 7133508139, 7133508137, 3535674400, 6754462980, 6754462990, 6754462992, 6516281350, 5300241801, 5300241802, 5300241803, 6318722590, 5528952390, 7133502524, 7133502525, 7133502526, 7133502527,  7133502528, 7133502529, 6191945630, 6704695930, 7133504486, 5107978820, 5107978920, 5107979020, 6586267601, 6586267605, 6586267699, 6586267701, 6586267705, 6586267799, 6586267801, 6586267805, 6586267899, 6586267901, 6586267905, 6586267999, 3535674410, 3535674430, 3535674460, 3535674490, 6704696930, 6704697030, 54306844, 2423652302, 5456961870, 5009022560, 5009022561, 5009022564, 5009017120, 5009017121, 5009017122, 5009017123, 5009017130, 5009023461, 5009024250, 5009017132, 5009017133, 5009017590, 5009017591, 5009017592, 5009017593, 5009017741, 5009028961, 1659000515, 1659000520, 5009024251, 5009024252, 5009024253, 1659000530, 1659000560, 1659000572, 1659000590,  1659000615, 1659000628, 1659000630, 1659000660, 1659000671, 1659000672, 1659000690, 5009006230, 5009006231, 5009006232, 5009006233, 5009004650, 5009004651, 5009006236, 5009006631, 5009014832, 1659044730, 5009004652, 5009004654, 5009004660, 5009004661, 5009004662, 5009004663, 1659044760, 1659044790, 1659058830, 1659058860, 1659058872, 1659058890, 5456937550, 5456937551, 5456937552, 5456937554, 5456937555, 5456937560, 5456937561, 5456937562, 5456937563, 5456937564, 5456946190, 5456946191, 5456946192, 5456946193, 5456946196, 5456949000, 5456949001, 5456949002, 5009028191, 6729604311, 6729604312, 6729605291, 4225403303, 4225403314, 4225403320, 4225403330, 6633693210,  6633693230, 6633693260, 6633693290, 2169524730, 2169524760, 2169524790, 2169524800, 2169524815, 2169524830, 2169524860, 2169524930, 2169524945, 2169524960, 2169525030, 6318727830, 6318733760, 6318744530, 6318744560, 6318744590, 6318751830, 5486829290, 5486829291, 5486829292, 5486829293, 5486829295, 5486829296, 5486829297, 5486829298, 5486829300, 5486829301, 5486829302, 5486829303, 5486829304, 5486829305, 5486829307, 5486846631, 5486846632, 5486846634, 5486846635, 5486846636, 5486846637, 4225403360, 4225403390, 4225404860, 5486829309, 5486830051, 5486830052, 5486830053, 5486830054, 5486830057, 5486830058, 4225421690, 6318751860, 6318751890, 6878805752, 6878805753, 6878805756, 6878805758, 6878805759, 6878820272, 6878820273, 6878820276, 6878820279,  6878820292, 6878820293, 2169525060, 2169560260, 2169560360, 2169560460, 2169560560, 6362953641, 6362953642, 1054402930, 1054405430, 1054411830, 6042950212, 6042950215, 6042950218, 6042950230, 6042950260, 6042950290, 6042950312, 6042950315, 6042950318, 6042950330, 6042950345, 6042950360, 6042950390, 6042950412, 6042950415, 6042950418, 6042950430, 6042950460, 6042950490, 6042950515, 6878820296, 6878820298, 6878820299, 6042950518, 6042950560, 1054415530, 1054415560, 1054415590, 6945211020, 6945211030, 1054410430, 1054410630, 1054410730, 6945211032, 6945211120, 6945211130, 6945211132, 6945211220, 6945211230, 6945211232, 6945211320, 6945211330, 6191963960, 6945211332, 6191957660, 6191957690, 6362929420, 6362929421, 6362929422, 6362929423, 6362929424,  6362929425, 6362929426, 6362929427, 6362929428, 6472506041, 6191900630, 6191900660, 6362915411, 6362915412, 6362915413, 6362915414, 6362915415, 6362915416, 6362915417, 6362915418, 6362915419, 6362929429, 6362933081, 6362933082, 6362933083, 6362933084, 6362933085, 6362933086, 7592190130, 6704696030, 6704696130, 6704696230, 6704696330, 6704696430, 6704696530, 6840502426, 6840503426, 1659093830, 1659093860, 1659093872, 1659093890, 6840503226, 9002901, 9002902, 9002914, 9002946, 9005501, 9005503, 9005515, 9005546, 9005707, 9005907, 9006607, 9006807, 9009001, 9009004, 9009013, 9009401, 9009403, 6191908960, 6191971120, 4988426501, 4988426601, 378021101, 378021105, 378027701, 378027705, 5515450880, 5515450890, 5515456520, 4306304603, 4306304606, 4306304612, 4306304615, 555003302, 555003305, 555015802, 555015804, 6807121453, 4229121090, 4229121190, 4229121290, 6918906221, 6362940271, 6362940272, 6362940273, 6362940274, 5107914120, 5107937420, 5107937520, 2423651702, 4335396953, 555015902, 555015904, 6042955401, 6042955501, 6042955601, 2423680002, 5321718330, 7051801570, 6704690907, 6704690914, 6704690915, 6704690920, 6704690921, 6704690928, 6704690930, 6704690960, 4306304619, 4306304624, 6626705130, 6626777106, 7051806630, 5528906106,  5528906160, 6704691230, 6704693530, 4335369930, 4335369953, 4335369960, 4354725110, 4354725210, 4354725310, 5456920954, 5456920956, 6472501591, 6362946691, 6362946692, 6362946693, 7051800150, 7051800151, 5486800700, 5486800702, 5486823610, 5486823613, 5486823614, 5486824630, 5486824631, 6704691030, 4249440901, 6918904891, 5009015280, 6815125001, 5129360701, 5129360710, 5129360766, 6868240910, 5456904300, 4258230010, 4258230016, 4258230020, 4258230110, 4258230116, 1013562201, 4258230120, 5009001310, 7643930210, 187410010, 67386313, 67386314, 67386315, 43063788, 43063794, 43063794, 43063797, 43063797, 504360195, 504360195, 504360195, 504360913, 51407525, 67544412, 67544412, 67544412, 67544412, 67544412, 67544412, 63187273, 6158002, 6158048, 63187888, 63187888, 63187888, 67544289, 67544289, 67544289, 67544289, 67544289, 67544289,  67544289, 67544289, 67544289, 67544289, 67544289, 67544289, 67544289, 67544301, 67544301, 67544301, 67544301, 67544301, 67544301, 67544301, 67544301, 51407525, 51407526, 55700564, 55700564, 55700564, 63187273, 63187273, 57664783, 57664783,  57664783, 57664784, 57664784, 57664784, 67544301, 67544301, 67544301, 43547406, 43547407, 43547406, 43547408, 43547407, 57664785, 57664785, 57664785, 57664786, 57664786, 57664786, 57664787, 51407525, 51407525, 51407525, 67544256, 67544288,  49999328, 71610040, 71610040, 551545452, 551545462, 551546831, 71610040, 60760300, 33261340, 33261340, 33261340, 691890493, 66267708, 66267708, 66267708, 66267708, 66267708, 66267748, 66267748, 66267748, 504360913, 504360913, 504360915, 504360915,  504362948, 504362948, 2283003, 2283003, 2283004, 66267748, 66267748, 66267748, 66267748, 66267904, 2283004, 2283005, 2283005, 57664273, 57664273, 57664273, 57664274, 57664274, 680714021, 680714021, 680714021, 680714021, 6032948, 6032948, 6032948,  6032948, 33261628, 1790164, 1790164, 1850063, 1850063, 1850063, 1850064, 1850064, 1850064, 1850065, 1850065, 1850065, 680714135, 680714135, 680714135, 680714135, 57664274, 57664275, 57664275, 57664275, 5550094, 5550095, 5550096, 5550097, 43547408,  43547407, 7818018, 7818018, 51407524, 51407524, 6157796, 6158001, 680714135, 43353277, 43353277, 43353277, 43353277, 43353277, 43353277, 43353277, 57664787, 57664787, 67544529, 67544529, 43353799, 43353799, 43353799, 713350113, 6032948, 6032948,  6032948, 6032948, 6032948, 6032948, 71610061, 687889940, 67544256, 67544256, 67544256, 67544288, 67544288, 705181199, 504361360, 687889378, 687889940, 687889940, 687889940, 687889940, 687889941, 687889941, 687889941, 687889941, 9046101, 9046102, 9046103, 3781910, 3781910, 3781910, 3781912, 3781912, 687889378, 687889378, 687889379, 687889379, 687889379, 687889495, 687889495, 687889495, 43353277, 43353277, 43353277, 43353277, 43353307, 43353308, 43353308, 71610061, 687889495, 687889495, 7815567, 7815567, 7815567, 7815569, 7815569, 7815569, 71610039, 71610040, 49884306, 49884307, 49884308, 49884309, 45865575, 45865575, 60429524, 60429524, 713350001, 713350001, 713350001, 713350001, 713350001, 713350001, 713350001, 713350001, 713350001, 60429524, 60429524, 60429524, 60429524, 60429524, 60429525, 60429525, 60429525, 687886989, 687886989, 687886989, 687886989, 687886989, 687887394, 687887394, 687887394, 713350001, 713350022, 713350022, 713350022, 713350022, 713350022, 713350022, 713350022, 713350022, 49884310, 713350113, 713350113, 713350333, 713350113, 713350113, 713350333, 713350333, 713350113, 60429525, 60429525, 60429525, 60429526, 60429526, 60429526, 49349319, 7818018, 713350113, 3781912, 3781914, 3781914, 61919485, 71610061, 51407526, 636293339, 636293339, 636293339, 687887394, 51407525, 51407524, 51407524,  51407525, 51407526, 63187603, 63187603, 63187603, 61786577, 5550098, 51407524, 705180644, 705180154, 705180259, 16714750, 16714750, 16714751, 16714751, 16714752, 16714752, 16729136, 636293339, 636293339, 636293339, 53217247, 53217247, 53217247,  63187226, 63187226, 63187226, 51079881, 51407524, 51407526, 71610023, 71610023, 71610023, 713350022, 713350022, 63739263, 63739264, 67544697, 67544697, 67544697, 67544697, 636291340, 636291340, 636291340, 636291340, 636291340, 636291340, 16729136,  16729137, 16729137, 16729138, 16729138, 51407524, 67544529, 67544529, 71610061, 67544256, 67544256, 67544256, 67544288, 67544256, 51655865, 33261029, 33261029, 33261029, 67544288, 67544288, 67544529, 67544529, 67544529, 52125958, 16714469, 16714469, 51079882, 51079883, 43547406, 53217179, 53217179, 53217179, 53217179, 930832, 930832, 930832, 930833, 930833, 930833, 930834, 930834, 67544697, 67544697, 67544697, 67544697, 530021306, 530021306, 530021306, 530021451, 53217326, 51407526, 35356719,  35356719, 53217179, 53217189, 53217189, 53217189, 53217189, 530021451, 530021451, 16714470, 16714470, 16714471, 16714471, 713350113, 713350333, 705181069, 713350333, 713350333, 51407525, 51407524, 51407524, 636291340, 636291340, 713350113, 71610039,  71610039, 67544256, 67544529, 71610061, 71610061, 67046914, 67046914, 67046914, 67046914, 67046914, 67046914, 67046914, 67046914, 67046916, 67046916, 67046916, 67046916, 67046916, 67046916, 67046916, 67046916, 35356719, 35356719, 35356722,  35356722, 35356722, 35356722, 67046918, 67046920, 67046920, 67046920, 67046920, 67046920, 67046920, 67046920, 67046920, 67046921, 67046922, 40058, 40068, 40098, 65084308, 65084309, 63304552, 63304552, 63304552, 63304552, 63304553, 63304553,  63304553, 63304553, 43063030, 43063030, 43063030, 43063030, 43063164, 43063350, 43063382, 43063382, 43063382, 43063382, 516724042, 516724042, 516724042, 516724043, 516724043, 516724043, 3780030, 3780030, 3780040, 3780040, 3780070, 67046904,  67046904, 67046904, 67046904, 67046904, 67046904, 67046904, 67046904, 67046907, 67046907, 67046907, 67046907, 67046907, 63304554, 63304554, 63304554, 67046907, 67046907, 67046907, 516724044, 516724044, 516724044, 55292304, 6649065020, 187065820, 187065920, 4306371804, 4306371806, 4306371809, 4306371810, 4306371812, 4306371815, 4306371821, 4306371830, 4306371860, 5515450740, 6729600841, 6729600842, 6729600843, 6729600844, 6729600846, 6729602912, 5515455540, 5515456530, 6318717302, 5515463428,  5515463448, 5515469635, 5515469638, 6729613491, 6729613492, 6729613495, 6729613496, 4306371890, 4306375130, 378027101, 378027105, 378034501, 378034505, 378047701, 378047705, 5515414920, 6754401915, 6754401920, 6754401930, 6754401940, 6754401945, 6754401953, 6754401955, 6754401960, 6754401970, 6754401975, 6754401980, 6754401990, 6754402015, 6754402030, 6754402045, 6754402053, 6318730910, 6318730915, 5043635921, 5043635922, 5043635924, 5043635925, 5043635931, 5043635932, 5258421312, 5258427332, 615804939, 6626706830, 6626706860, 6626706890, 6626706910, 6626706915, 6626706930, 6626706990, 6626777604, 6626777606, 6807133301, 6807133303, 6807133305, 172392560, 172392570, 172392660, 172392670, 172392680, 172392760, 172392770, 172392780, 6318730920, 6318730930, 6318730960, 6318730990, 6318754415, 6318754420, 6318754430,  5186206201, 5186206205, 5186206210, 5186206301, 5186206305, 5186206310, 5186206401, 5186206405, 5186206410, 5570043910, 5570043920, 5570043930, 5570043960, 5570043990, 5570044202, 5570044230, 5570044260, 179142920, 179142930, 179142950, 179143120,  179143130, 179143150, 527176765, 527176836, 6807141811, 4335327615, 4335378315, 4335378320, 4335378330, 4335378345, 4335378353, 4335378355, 4335378360, 4335378370, 603321502, 603321510, 603321516, 603321520, 603321521, 603321522, 603321528, 603321532, 603321534, 4335327630, 4335327645, 4335327653, 4335327660, 4335327670, 4335327675, 4335327680, 4335327815, 4335327820, 4335327830, 4335327845, 4335327853, 4335327855, 4335327860, 4335327870, 4335327875, 4335378375, 4335378380, 4335378390, 4335382030, 4335382053, 4335382060, 4335382070, 4335382530, 4335327880, 4335327890, 4335328130, 4335328153, 4335328160, 4335328170, 5570044290, 6807132701, 6807132702, 6807132703, 6807132704, 6807132706, 615780039, 6868265020, 6878892070, 6878892071, 6878892072, 6878892073, 6878892074, 6878892075, 6878892076, 6878892078, 6878892079, 6878899360, 6878899361, 6878899362, 6878899363, 6878899364, 6878899365, 6878899366,  6878899368, 6878899369, 6878899370, 6878899371, 6878899372, 6878899373, 6878899374, 6878899375, 6878899376, 6878892080, 6878892081, 6878892082, 6878892083, 6878892084, 6878892085, 6878892086, 6878892088, 6878892089, 6754402060, 6754402070, 6754402075, 6754402080, 6754402090, 1263452955, 1263452980, 1263452991, 4335382553, 4335382560, 4335382570, 904588061, 1263469859, 1263469871, 1263469891, 1263452996, 7133506161, 7133506162, 7133506163, 7133506164, 7133506165, 7133506166, 7133506167, 7133506168, 7051800140, 7051801620, 6318784606, 6318784610, 6318784615, 6318784620, 6318784630, 3326171700, 3326171760, 409321312, 409321310, 7133501420, 7133501421, 7133501422,  7133501423, 6868265220, 6868265520, 6318754460, 6318754490, 6178699002, 6878868510, 6878868511, 6878868512, 6318784660, 6318784690, 6318786715, 6318786730, 6318786760, 6318786790, 5009022494, 6878869420, 6878869421, 6878869422, 6878869423, 6878869424,  6878869425, 6878869426, 6878869428, 6878869429, 7133504950, 7133504951, 7133504952, 7133504953, 7133504954, 7133504955, 7133504956, 7133504957, 7133504958, 7133504959, 6878868513, 6878868514, 6878868515, 6878868516, 6878868518, 6878868519, 6191908830, 6191908860, 6191908872, 6878899378, 6878899379, 409127332, 6318717310, 6318717315, 6318717320, 6318717330, 6318717360, 6318717390, 5009001930, 5009001932, 5009001933, 5009001934, 5009001935, 5009001938, 5009001944, 5009001956, 6191937760, 1170460001, 3326103405, 3326103407, 3326103410, 3326103414, 3326103429, 3326103430, 3326103431, 3326103460, 3326103490, 3326103505, 3326103507, 3326103508, 3326103510, 3326103520, 5009026310, 7133501424, 7133501425, 7133501426, 7133501427, 7133501428, 7133501429, 5528909201, 5528909202, 5528909206, 5528909210, 5528909220, 5528909225, 5528909230, 5528909240, 5528909260, 5528909290, 6362915230, 6362915231, 6362915232, 6362915233,  6362915234, 6362915235, 6362915236, 6362915237, 6362915238, 5107928420, 5107928520, 5107928620, 7133504440, 7133504441, 7133504442, 7133504443, 7133504444, 6807133306, 6807133309, 6807140303, 6807140305, 6807140306, 6807140309, 6809475062, 6178637702, 6178678208, 6373907310, 6754472015, 6754472030, 6754472045, 6754472053, 6754472060, 6754472070, 6362915239, 5321718400, 5321718402, 5321718405, 5321718407, 5321718408, 5321718410, 5321718414, 5321718420, 5321718429, 5321718430, 5321718431, 5321718460, 5321718490, 5321718500, 5321718502, 5321718505, 5321718507, 5321718508, 5321718510, 5321718514, 5321718520, 5321718529, 5321718530, 5321718531, 5321718560, 5321718590,  5528909298, 3535671602, 3535671605, 3535671610, 3535671615, 3535671620, 3535671630, 3535671660, 3535671690, 3535672400, 3535672401, 3535672402, 3535672420, 3535672430, 3535672460, 6754472075, 6754472080, 6754472090, 7133504445, 7133504446, 7133504447, 7133504448, 7133504449, 93613732, 93613832, 93613932, 6191908890, 6191913190, 6191940930, 3326103530, 3326103560, 5300233401, 5300233402, 5300233403, 6318717321, 3535672490, 5043656201, 5043656202, 1785631882, 6362973281, 6362973282, 4306384030, 6704694307, 6704694314, 6704694315, 6704694320, 6704694321, 6704694328, 6704694330, 6704694360, 6704694407, 6704694414, 6704694415, 6704694420, 6704694421, 6704694428,  6704694430, 6704694460, 6704694507, 6704694514, 6704694515, 6704694520, 6704694521, 6704694528, 6704694530, 6704694560, 6704694630, 6704694730, 54318863, 5456909360, 5456909362, 5456909363, 5456909364, 5456909365, 5456909368, 5456909470, 5456909476, 5456909490, 5456909491, 5456909492, 5456909493, 5456909494, 5456909495, 5456909497, 5456960440, 5456966400, 5009019131, 4254955420, 4254955430, 4254955460, 4254955490,  4254955492, 4254960320, 4254960330, 4254960360, 4254960390, 4254960392, 5009023150, 5009019132, 5009019134, 5009019135, 5009019591, 5009019592, 5009019594, 5009019595, 5009020634, 1659006930, 1659006960, 1659006990, 1659007002, 1659007003, 1659007015, 1659007020, 1659007030, 1659007045, 1659007060, 1659007072, 1659007075, 1659007090, 1659007104, 1659007110, 1659007115, 1659007120, 1659007128, 1659007130, 1659007145, 1659007156, 1659007160, 1659007162, 1659007172, 1659007175, 1659007190, 5456953544, 6918939251, 6918939255, 5456947644, 6729605061, 6633603394, 6633647802, 6633647806, 6633647810, 6633647815, 6633647820, 6633647830, 6633647860, 6633647890, 6633647894, 6633657930, 2169526330, 2169526390, 2169526402, 2169526410, 2169526412, 2169526415, 2169526420, 2169526430, 2169526445, 6191936730, 6191936760, 1054416130, 1054416160, 2169526460, 2169526490, 2169526505, 2169526515, 2169526530, 2169526548, 2169526560, 2169526590, 6472539251, 6472539261, 6191936790, 2169588910, 6472556201, 5486800590, 5486800591, 5486800592, 5486800593, 5486800594, 5486800596, 5486800598, 5486806171, 5486806172, 5486809880, 5486809881, 5486809882, 5486809883, 5486809884, 5486800599, 6704694130, 6704694230, 5486809885, 5486809886, 5486809887, 5486809888, 5486809889,  5486821264, 5486821265, 5486821266, 5486821267, 54318544, 6840505426, 140000401, 140000501, 140000514, 140000601, 140000614, 6508431210, 6508431310, 5528911706, 5528911712, 5528911720, 5186206901, 5186207001, 591074401, 591074501, 2169522030, 143336701, 143336705, 143336730, 143337001, 143337005, 143337030, 6878804083, 6878804086, 6878804089, 5295923614, 5295923615, 5295923620, 5295923624, 5295923630, 5295923660, 5456908980, 5456923762, 2169536330, 378441501, 378443001, 5528903830, 5486800921, 5486800922, 641600010, 641600125, 641600210, 641600325, 187006301, 187006310, 187006350, 187006401, 187006410, 187006450, 187006501, 187006510, 187006550, 6508446310, 5456909271, 1659081330, 1659081360, 1659081372, 1659081390, 1659093730, 1659093760, 1659093772, 1659093790, 1310708505, 1310708310, 1310708401, 1310708501, 1310708510, 6729601431, 6729601432, 1310708399, 5515455500, 5515455900, 5515456550, 1310708599, 4999912210, 4999912230, 4999912260, 4999912290, 4999926630,  6807130103, 6807130104, 6807130106, 121077001, 6318753606, 6318753615, 6318753620, 6318753630, 6318753660, 6754414215, 6754414230, 6754414245, 6754414253, 6754414260, 6754414270, 6754419115, 6754419120, 6754419130, 6754419145, 6754419153, 6754419170, 6754419175, 6754419180, 6754419190, 6754419192, 6754426110, 6754426115, 6754426130, 6754426140, 6754426145, 6754426153, 6754426155, 6754426160, 615800339, 615800439, 615805039, 615807539, 615807639, 615807739, 641604425, 641604525, 641604610, 641604710, 641604825, 6729610601, 6729610603, 6729612281, 6729612771, 6729613321, 6729615391, 6729615401, 6330477230, 6330477290, 6330477301, 6330477305, 6330477310, 6330477330, 6330477390, 6330477401, 6330477405, 6330477410, 6330477430, 641604925, 641605010, 641605110, 641620725, 7133502777, 7133503297, 7133503291, 7133502042, 7133502044, 7133503296, 7133503298, 7133502045, 7133502771, 7133502048, 7133503294, 7133502047, 7133503293, 5043639601, 5043639602, 7051812550, 6330477201, 6330477205, 6330477210, 4306304804, 4306304806, 4306304815, 6076045730, 4306305706, 6807130963, 6807130964, 6807131216, 6318753690, 5038370530, 6626713330, 6626713345, 6626742730, 6626742760, 6626742790, 6626760845, 6626760860, 7133502046, 7133502049, 7133503292, 7133503295, 7051810450, 5212593160, 409677805, 6878874111, 6878874118, 179013130,  179013160, 179014830, 179014860, 179016330, 179016360, 179018530, 179018560, 79018630, 179018660, 6808473601, 6808474201, 6754426170, 6754426175, 6754426180, 6754426192, 1263486700, 1263486771, 1263486791, 1310708305, 6626760890, 6626779806, 6626783504, 6626783506, 4335358110, 6807130109, 4999980530, 6807132271, 6807132272, 6807132273, 6808475401, 591024001, 591024005, 591024010, 591024101, 591024105, 591024110,  591024201, 6807132275, 6807132276, 6807132277, 6807132279, 6807132423, 6807132424, 6807132425, 6807132428, 1310708410, 6878874113, 6878874116, 3326136430, 3326136440, 3326136460, 6878898391, 6878898393, 6878898396, 6878898398, 5570061430, 6878898399, 6878898401, 6878898403, 6878898406, 6878898408, 6878898409, 603424621, 603424628, 603424632, 4335358170, 4335358175, 4335358180, 4335358192, 904598061, 904598161, 904598261, 904600740, 904600760, 904600761, 904600860, 904600861, 904600960,  904600961, 378232101, 378232105, 4335358115, 4335358120, 4335358130, 4335358145, 4335358153, 4335358155, 4335358160, 6330477490, 615780539, 781537101, 781537105, 781537701, 781537705, 781537901, 781537905, 781540401, 6878892581, 6878892583, 6878892586, 781540405, 781540601, 781540605, 781540801, 781540805, 6878895111, 6878895113, 6878895115, 6878895116, 5009031410, 7133506554, 7133506556, 7133506551,  7133506553, 7133506552, 7133506555, 7133506557, 6878895118, 6878895119, 7133502041, 7133502773, 7133502775, 6318757603, 6318757630, 6318757660, 6878802411, 6878802413, 6878802415, 6878802416, 6878802418, 6878802419, 7051801550, 7051801560, 6318786803, 6318786805, 6318786806, 6318786810, 6318786820, 6318786830, 7133502040, 7133502043, 6318786860, 591024205, 591024210, 6878874119, 6068735501, 6362929531, 6362929532, 6362929533, 6362929534, 378245701, 378245710, 378277701, 378277705, 7051804450, 7051804860, 5321720802, 5321720805, 5321720830, 603424721, 603424728, 603424732, 6878899821, 6878899823, 6878899826, 6878899828, 6878899829, 7133508063, 6318786890, 7051802750, 6318757602, 409153931, 6178642860, 6178672602, 6178690202, 6178690260, 6178691802, 6318757690, 6191906260, 7133502772, 7133502774, 6373949904, 6373949910, 6373950004, 1310708405, 409198530, 7133508068, 409677802, 409677902, 409678002,  1747804001, 6373950010, 6373950110, 6373980241, 6373980242, 6373980243, 6373980244, 6373980341, 6373980342, 6373980343, 6362929535, 6362929536, 6362929537, 6362929538, 3326106805, 3326106830, 3326106860, 3326106930, 3326106960, 5321720845, 5321720860, 5321720890, 5321721615, 5321721630, 5321721645, 5321721660, 5321721690, 6754498215, 6754498230, 6754498245, 6754498253, 6754498260, 6754498270, 6754498275, 6754498280, 6754498290, 5009002730, 6362912330, 6362912331, 6362912332, 6362912333, 6362912334, 6362912335, 6362912336, 6362912337, 6362912338, 6362912339, 6373980344, 6373980441, 6373980442, 6373980443, 6754487010, 6754487015, 6754487020, 6754487030, 6754487045, 6754487053, 6754487060, 6754487070, 6754487075, 6754487080, 6754487092, 6754487910, 6754487915, 6754487920, 6754487930, 6754487945, 6754487953, 6754487955, 6754487960, 6754487970, 6878892588, 6878892589, 7133508061, 7133508065, 7133508066, 5165582253,  5212591302, 5212593102, 6068736701, 7161000810, 7161000815, 7161000830, 7161000845, 7161000853, 7161000860, 7161000870, 5528940230, 5528940290, 5528948702, 5528948704, 5528948706, 5528948708, 5528948709, 5528948715, 5528948720, 5528948730, 5528948760, 5528948779, 5528948790, 5528948798, 5528959401, 5528959430, 5528959460, 7632982611, 6754498292, 5107938620, 5107938656, 5107938720, 5107941720, 5107941756, 6508444114, 6508444118, 6508444120, 6508444136, 6508444214, 6508444218, 6508444220, 6508444236, 6508444314, 6508444318, 3535684405, 3535684415, 3535684430, 3535684460, 3535684490, 5528959490, 6516268784, 6931590401, 6931590405, 6508444320, 6931590410, 6931590501,  6931590505, 6931590510, 6931590601, 6931590605, 6931590610, 6754487975, 6754487980, 6754487992, 1310708301, 1310708499, 5300252901, 5300252902, 5300252903, 5300277201, 5300277202, 5300277203, 7133508064, 7133508067, 7133508062, 7133502776, 54353244, 4254955830, 4254965505, 4254965510, 1659014315, 1659014330, 1659014360, 1659014372, 5009002720, 5009002721, 5009002750, 6918942461, 6918942465, 1659058415, 1659058430, 1659058460, 1659058472, 1659058490, 5456915850, 5456915851, 5456915852, 5456915855, 1659014390, 5456954010, 5456921730, 5456921734, 5456926870, 5456926874, 5486860100, 6729600421, 6729603061, 5515458780, 5515458860, 6729607261, 6318741730, 6318741760, 6318741790, 5515414820, 2169523800, 2169523815, 2169523830, 2169523860, 2169523890, 2169523903, 4225409620, 4225409630, 4225409660, 4225409690, 4225411130, 2169523915, 2169523930, 2169523960, 2169523990, 2169524003, 2169524030, 2169524060, 4225411190,  4225411305, 4225411315, 4225411330, 4225411360, 4225411390, 4225425403, 5486835660, 5486835661, 5486835662, 6633604790, 6633636344, 6633680090, 1054408530, 1054424430, 6191958430, 6191958490, 6472502421, 6472507721, 6472507731, 6178631060, 6178691902, 6191914330, 6191914360, 6318720830, 6472560441, 3335822130, 1054426910, 6412590401, 6412590405, 6412590410, 6412590501, 6412590505, 6412590510, 6412590601, 7592124130, 6412590605, 6412590610, 5486813380, 5486813381, 6318720860, 6318720890, 5486800612, 5486800613, 5486813382, 5486813383, 5486813384, 5486813386, 5486813387, 5486813388, 5486813389, 5486821450, 5486821452, 5486800614, 5486800615, 5486800616, 5486821453, 5486821454, 5486821455, 5486821456, 5486821459, 6704698030, 6704698130, 6704698230, 641605610, 641605710, 641605725, 641605910, 641606010, 641606110, 641606125, 258405501, 5258405601, 5258459603, 5766463445, 641606310, 641606325, 641619010,  641620925, 641621010, 641621110, 4778158868, 4778158917, 1747852302, 1747852305, 1747852310, 1747852325, 1747852355, 1747852401, 1747852402, 1747852405, 6332341110, 6332341112, 6332341125, 6332341200, 1747852410, 1747852415, 7086060002, 7086060105, 7086060110, 4778158991, 6332341202, 6332341205, 6332341210, 6332341225, 5258405901, 7604500120, 7604500210, 7604500320, 5515428895, 5766463343, 5766463444, 5009006830, 5009006831, 5009007380, 5009007381, 6529341125, 6529341210, 6529341310, 6529341410, 6529341510, 6529341625, 6529341725, 5253300116, 5253300172, 5253300105, 5253300101, 5253300160, 5253300175, 5253300131, 5253300177, 5253300171, 5253315775, 5253315745, 5258430801, 5258430802, 5564876201, 5564876202, 5564876203, 5564876204, 5564876205, 5564876206, 5564876301, 5564876302, 5564876303, 5564876304, 5564876305, 5564876306, 5564876307, 5564876308, 5564876401, 5564876402, 5564876403, 5564876404, 5564876501,  6809476262, 6330420518, 1785601461, 6808310601, 6808310801, 6808310802, 6809476462, 409230521, 409230550, 409230505, 409230517, 409230612, 409230662, 409230760, 409230849, 409230850, 409230801, 409230802, 409258705, 409259603, 6467976201, 6467976202, 6467976203, 6467976204, 6467976205, 6467976206, 6467976301, 6467976302, 6467976303, 6467976304, 6467976305, 6467976306, 6467976307, 6467976308, 6467976401, 6467976402, 6467976403, 6467976404, 6467976501, 6467976502, 6467976503, 5564876502, 5564876503, 5564876504, 1785602051, 1785602052, 1785635661, 1785635663, 2315560041, 2315560141, 2315560142, 409259605, 409259652, 409259653, 5515447435, 5515478315,  6467976504, 574015004, 54356699, 5456952550, 5456952551, 5456951160, 5456951161, 5486857110, 6825889731, 6825889811, 6754411730, 6754411753, 6754411760, 6754411770, 6754426830, 6754426853, 6754426860, 6754426870, 228206710, 228206750, 228206910, 228206950, 228207310, 781280901, 781280905, 781280910, 4335396530, 4335396553, 4335396560, 4335398030, 781281001, 781281005, 781281010, 6178691002, 6754429430, 6754429453, 6754429460, 4335307430, 6258481201, 6258481301, 6258481401, 1659030430, 1659030460, 1659030490, 6182516510, 7199326510, 5456965680, 5009014740, 406991403, 406991501, 406991503, 406991601, 406991701, 6840501306, 6754437310, 6754437315, 6754437320, 6754437328, 6754437330, 6754437353, 6754437359, 5528966001, 5528966030, 5528966060, 5528966090, 6754403810, 6754403814, 6754403815, 6754403820, 4999934615, 4999934630, 4999934660, 6754403828, 6754403830, 6754403845, 6754403853, 6754403860,  6754412715, 6754412720, 6754412728, 6754412730, 6754412753, 6754412759, 6754412760, 6754413610, 6754413614, 6754413615, 6754413620, 6754413628, 6754413630, 6754413645, 6754413653, 6754413660, 6754421710, 6754421714, 6754421715, 5515450900, 5515455910, 781220101, 781220105, 781220201, 781220205, 5348964801, 5348964803, 5348964805, 5348964806, 5348964807, 5348964810, 5348964901, 5348964903, 5348964905, 5348964906, 5348964907, 5348964910, 5348965001, 5348965003, 5348965005, 5348965006, 5348965007, 5348965010, 5348965101, 6754421720, 6754421728, 6754421730, 6754421745, 6754421753, 6754421760, 6754428710, 6754428715, 6754428720, 6754428728, 6754428730,  6754428753, 6754428759, 6754428760, 4335331215, 4335331230, 179147001, 179147030, 179147050, 6807140833, 6807141001, 228207610, 228207650, 228207710, 228207750, 615780839, 615780939, 378311001, 378312093, 6878871630, 6878871639, 6878898641, 6878898643, 6878898646, 6878898649, 6878898651, 6878898653, 904643604, 904643660, 378401001, 378401005, 378401077, 440847401, 440847405, 378505001, 378505005, 378505077, 440847430, 440847501, 440847505, 440847515, 440847530, 440847601, 5348965103, 5348965105, 5348965106, 5348965107, 5348965110, 440847605, 440847615, 440847630, 440847701, 440847705, 440847730, 406995903, 406996001, 7133503151, 7133503154, 7133503153, 7133503155, 6626732130, 6626741530, 6626741560, 6878869861, 6878869863, 6878869866, 6878869868, 6878869869, 7133505271, 7133505272, 7133505273, 7133505274, 7133505275, 7133505276, 7133505277, 7133505278, 6754437360, 179004570, 6318771815, 6318771820, 4229179701, 6042950601, 6042950605, 6042950701, 6042950705, 6878871636, 6878871633, 5321720530, 5321720560, 5321720590, 7133503156,  6878869041, 6878869043, 6878869046, 6878869048, 6878869049, 6373912510, 6878898656, 6878898659, 4335371130, 4335371153, 4335371160, 7051806470, 5107941820, 7133505279, 5043646281, 5043646282, 5043646291, 5043646292, 5043646301, 7161000430, 7161000453, 5107941821, 5107941920, 6787714601, 6787714605, 6787714701, 6787714705, 6787714801, 6787714805, 6362916191, 6362916192, 6362916193, 6362916194, 6362916195, 6362916196, 6362916211, 6362916212, 6362916213, 6362916214, 6362916215, 6373923110, 6373987710, 6516255603, 6516255610, 6516255611, 6516255650, 6516255703, 6516255710, 6516255711, 6516255750, 6516258303, 6516258310, 6516258311, 6516258350, 6516258403, 7161000460,  5528919601, 5528919614, 5528919630, 5528919660, 5528919690, 6318771830, 6318771860, 6318771890, 4335331245, 4335331253, 4335331260, 6808454921, 7133503152, 6516258410, 6516258411, 6516258450, 3535666315, 3535666330, 3535666360, 3535666390, 3535666415, 6373900333, 3535682130, 6787714830, 6787714901, 6787714905, 6787714930, 3535666430, 3535666460, 3535666490, 6362916216, 6362916217, 6362916218, 6362916219, 6807141002, 6807141003, 6807141006, 6807141008, 4335315230, 4335315253, 4335315260, 4335326015, 4335326030, 4335326053, 5456909050, 4254956112, 4254956130, 4254956160, 4254956190, 5009023561, 5009023910, 1659021428, 1659021430, 1659021445, 1659021460, 1659021471, 1659021490, 5456917261, 5456917264, 5009001860, 5009002851, 4254965112, 4254965130, 4254965160, 4254965190, 2169528230, 2169528260, 2169528278, 2169528330, 2169528390, 6191921430, 6191926330, 6191926430, 6191943830, 6318732400, 6318749700, 6318749730,  6318749760, 6318749790, 5295945900, 5295945910, 5295945912, 5295945915, 5295945920, 5295945930, 5295945960, 5295953510, 5486800380, 5486800381, 5295953512, 5295953515, 5295953520, 5295953524, 5295953528, 5295953530, 5295953540, 5295953560, 5486800382, 5486800383, 5486800384, 5486800385, 5486800386, 5486800392, 5486800393, 5486800394, 5486800395, 6318713230, 4003212020, 4003212024, 4003212026, 3335833230, 4003212124, 4003212126, 4003212220, 4003212224, 4003212226, 4003212324, 4003212326, 6472514601, 6472522021, 5486800396, 9001702, 9001755, 9001758, 5043637181, 5043690361, 5976237174, 5976237179, 5976237183, 5976237184, 5976237189, 6754472530, 6754472553, 4335345130, 4335345153, 4999905530, 5528978702, 5528978730, 5528978760, 5009005220, 5009005221, 5456963760, 1659023630, 1659023660, 1659023690, 5456939660, 5456939661, 5456939663, 6362929560, 6362929561, 6362929562, 6362929563, 6362929564, 6362929565, 6362929566,  6362929567, 6362929568, 6362929569, 2169528400, 2169528410, 2169530310, 2169530330 |
| MMSL Synonym | 1130, 11976, 12083, 12084, 12099, 12120, 12127, 12128, 12164, 12215, 12283,  12295, 12552, 13184, 13202, 1327, 1345, 15468, 15469, 15543, 15544, 15703,  15753, 15754 158643, 1598, 16048, 16072, 16084, 16181, 16344, 16345, 16355,  16536, 16546, 16570, 16571, 16729, 16844, 16845, 17128, 17143, 17174, 17175,  17225, 17435, 17454, 17592, 17593, 17708, 17709 177187, 17756, 17757, 17774,  17869, 17870, 17871, 18085, 18187, 18396, 18413 184409, 18509, 18510, 185861,  1870 187714, 18787, 18833, 18835, 18894, 18895, 19073, 19096, 19313, 19375,  19376, 19580, 19843, 2, 20253 203101, 20356 205271, 20756, 20800, 20831,  20996, 20997, 2104, 21196, 21207, 21209, 21210, 21259, 21345, 21695, 21696,  22128, 22607, 22619, 22620, 2434, 27209, 27871, 28046 298277, 3018 319984,  32073, 330423, 330424, 330425, 344291, 350697, 3763, 377, 37749, 3795, 38475,  46261, 46262, 46265, 46266, 46267, 46268, 46269, 46277, 46834, 46836, 46837,  46838, 46839, 46840, 46841, 46842, 46843, 46897, 47582, 47583, 537, 569,  63780, 63781, 63782, 63783, 64351, 64352, 64396, 64397, 73245, 791, 90414 |
| MMSL Drug ID | d00040, d00148, d00149, d00168, d00189, d00197, d00198, d00238, d00301, d00384, d00397,  d00915, d04557, d03492, d05416, d03462 |

**Table 13a.** List of codes used to define medications for opioid use disorder (MOUD) treatment

| **MOUD type** | **Code Type** | **Codes** |
| --- | --- | --- |
| buprenorphine (Brixadi, Bunavail, Cassipa,  Probuphine, Sublocade, Suboxone, Subutex, Zubsolv) | NDC | 54017613, 54017713, 54018813, 54018913, 93537856, 93537956, 93572056, 93572156, 228315303, 228315403, 228315473, 228315503, 228315567, 228315573, 228315603, 378092393, 378092493, 378876716, 378876793, 378876816, 378876893, 406192303, 406192403, 406800503, 406802003, 490005100, 490005130, 490005160, 490005190, 781721606, 781721664, 781722706, 781722764, 781723806, 781723864, 781724906, 781724964, 12496010001, 12496010002, 12496010005, 12496030001, 12496030002, 12496030005, 12496120201, 12496120203, 12496120401, 2496120403, 12496120801, 12496120803, 12496121201, 12496121203, 12496127802, 12496128302, 2496130602, 12496131002, 16590066605, 16590066630, 16590066705, 16590066730, 16590066790, 3490927003, 23490927006, 23490927009, 35356000407, 35356000430, 35356055530, 35356055630, 2291017430, 42291017530, 42858050103, 42858050203, 43063018407, 43063018430, 43063066706, 3063075306, 43598057901, 43598057930, 43598058001, 43598058030, 43598058101, 43598058130, 3598058201, 43598058230, 47781035503, 47781035511, 47781035603, 47781035611, 47781035703, 7781035711, 47781035803, 47781035811, 49999039507, 49999039515, 49999039530, 49999063830, 9999063930, 50090292400, 50268014411, 50268014415, 50268014511, 50268014515, 50383028793, 0383029493, 50383092493, 50383093093, 52427069203, 52427069211, 52427069403, 52427069411, 2427069803, 52427069811, 52427071203, 52427071211, 52440010014, 52959030430, 52959074930, 3217013830, 53217024630, 54123011430, 54123090730, 54123091430, 54123092930, 54123095730, 4123098630, 54569549600, 54569573900, 54569573901, 54569573902, 54569639900, 54569640800, 4569657800, 54868570700, 54868570701, 54868570702, 54868570703, 54868570704, 54868575000, 5045378403, 55700014730, 55700018430, 55700030230, 55700030330, 58284010014, 59385001201, 9385001230, 59385001401, 59385001430, 59385001601, 59385001630, 60429058611, 60429058630, 0429058633, 60429058711, 60429058730, 60429058733, 62175045232, 62175045832, 62756045983, 2756046083, 62756096983, 62756097083, 63629402801, 63629403401, 63629403402, 63629403403, 3629409201, 63874108403, 63874108503, 63874117303, 65162041503, 65162041603, 66336001630, 8071138003, 68071151003, 68258299103, 68258299903, 68308020230, 68308020830 |
|  | MMSL Synonym | 1819, 12512, 24676, 45125, 45126, 45128, 45204, 45205, 45231, 45232, 45311, 45312, 45342, 45343, 72461, 72462, 72463, 176533, 176535, 176536, 176558, 186680, 196662, 196663, 196664, 196665, 203841, 204727, 204728, 204743, 204744, 204745, 206241, 227088, 227090, 227093, 227095, 227101, 227102, 227103, 233047, 233048, 236126, 236127, 236128, 236129, 237313, 237314,  237315, 237316, 237317, 237318, 237319, 241413, 241420, 241421, 254927, 254928, 267706, 267707, 267708, 267709, 267710, 351264, 351265, 351266, 351267, 352364, 352990, 904870, 904876, 904880, 1010600, 1010603, 1010604, 1010606, 1010608, 1307056, 1307058, 1307061, 1307063, 1431077, 1431083, 1431104, 1432969, 1542391, 1542997, 1544853, 1597570, 1597575, 1666385, 1996189, 199619 |
|  | MMSL Drug ID | d00840, d04819 |
|  | HCPCS | G2068, G2069, G2070, G2071, G2072, G2079, J0570, J0571, J0572, J0573, J0574, J0575, J0592 |
| naltrexone (Vivitrol, Revia, Depade) | NDC | 001850039, 004061170, 005550902, 009047036, 167290081, 422910632, 430630469,  430630591, 473350326, 500902866, 500903076, 500903929, 500904925, 504360105,  512240206, 521250727, 532170261, 548685574, 621350242, 636291046, 636291047,  636295304, 680712156, 680712721, 680840291, 680940853, 687887084, 691890499,  693643143, 705181146, 705181312, 705182718, 713350014, 713351480, 713352062,  721621566, 765191160, 657570300, 657570301, 512850275, 56001122, 56001130, 56001170, 56007950, 56008050, 185003901, 185003930, 406009201, 406009203, 406117001, 406117003, 555090201, 555090202, 16729008101, 16729008110, 42291063230, 43063059115, 47335032683, 47335032688, 50090286600, 50436010501, 51224020630, 51224020650, 51285027501, 51285027502, 52152010502, 52152010504, 52152010530, 54868557400, 63459030042, 63629104601, 63629104701, 65694010003, 65694010010, 65757030001, 65757030202, 68084029111, 68084029121, 68094085362, 68115068030 |
|  | MMSL Synonym | 167360, 12838, 72424, 228674, 18420, 167361, 167363, 167359, 167323, 81542, 11621, 136490, 136489, 228676, 187249, 167362, 137294, 244063, 167364, 81544, 81543, 1157, 15708 |
|  | MMSL Drug ID | d01406, d07135, d07472 |
|  | HCPCS | G0723, J2315 |
|  | ICD-10 Procedure | HZ84ZZZ, HZ94ZZZ |
| methadone (Methadose)^1^ | NDC | 00054039168, 00054039268, 00054070920, 00054070925, 00054071020,  00054071025, 00054121842, 00054355344, 00054355563, 00054355663, 00054454725, 00054457025, 00054457125, 00054855324, 00054855424, 00406052710, 00406054034, 00406151056, 00406254001, 00406345434, 00406575501, 00406575562, 00406577101, 00406577162, 00406872510, 00527192736, 00904653061, 42806031701, 49999084030,  49999084060, 54868440800, 60687021401 65100050102010, 66689069479,  66689069579, 66689071116, 66689071216, 66689089840, 67457021720,  67877011601, 68084073801, 68462080001, 68462080101, 70092116143 |
|  | MMSL Synonym | 109245, 11986, 152751, 16886, 169623, 17181, 17501, 17502, 177659, 177660, 17840, 17841, 1981,  198533, 1990745, 20893, 21360, 21577, 21764, 218337, 23072, 254686, 30953,  318373, 345780, 3910, 6813, 728129, 756132, 756133, 763509, 853463, 864706, 864708, 864712, 864714, 864718, 864720, 864737, 864761, 864769, 864978, 864980, 991147, 991149 |
|  | MMSL Drug ID | d00050 |
|  | HCPCS | G2067, G2078, H0020, S0109, J1230 |
|  | ICD-10 Procedure | HZ81ZZZ, HZ91ZZZ |
| Other types | ICD-10 Procedure | HZ82ZZZ, HZ85ZZZ, HZ86ZZZ, HZ92ZZZ, HZ95ZZZ, HZ96ZZZ |

**1** prescriptions included only from outpatient encounters

**Supplemental Table 13b.** List of codes used to define medications for alcohol use disorder (MAUD) treatment

| **MAUD type** | **Code Type** | **Codes** |
| --- | --- | --- |
| acamprosate (Campral) | NDC | 000935352, 002584000, 003786333, 009047213, 101350636, 422910104, 510790241,  606870121, 683820569, 684620435, 691890437, 707711057, 004563330, 681514760 |
|  | MMSL Synonym | 48384, 48401, 59284, 59283 |
|  | MMSL Drug ID | d04986 |
| disulfiram (Antabuse) | NDC | 000540356, 000540357, 000935035, 000935036, 003784140, 003784141, 006033432,  006033433, 427940028, 477810607, 604290196, 621350431, 621350432, 636296854,  649800171, 649800172, 681512694, 512850523, 512850524, 548685034 |
|  | MMSL Synonym | 12827, 18860, 18861, 17279, 17278 |
|  | MMSL Drug ID | d01389 |
|  | ICD-10 Procedure | HZ83ZZZ, HZ93ZZZ |
| topiramate (Topamax, Topiragen, Eprontia, Qudexy, Trokendi) | NDC | 000930155, 000937219, 000937220, 000937335, 000937336, 000937540, 001439755,  001439756, 001439757, 001439758, 002450707, 002450708, 002450709, 002450710,  003786101, 003786102, 003786103, 003786105, 004802356, 004802357, 004802358,  004802359, 006157562, 006157563, 006157564, 006157565, 006158138, 006158139,  006158140, 008320707, 008320708, 008320709, 008320710, 008321071, 008321072,  008321073, 008321074, 008321075, 009046016, 009046017, 009046018, 009046928,  009046929, 103700365, 103700366, 103700367, 103700368, 105440489, 105440628,  105440847, 126340083, 126340452, 126340453, 136680031, 136680032, 136680033,  136680034, 162520568, 162520569, 165710705, 165710706, 165710707, 165710708,  165900817, 165900824, 165900825, 216950162, 216950205, 216950348, 216950349,  272410227, 272410228, 272410229, 272410230, 293000115, 293000116, 293000117,  293000118, 317220181, 317220182, 317220183, 317220184, 317220278, 317220279,  317220280, 317220281, 332610106, 332610400, 332610480, 353560469, 353560470,  353560471, 353560472, 425490619, 425490620, 425490621, 430630094, 430630114,  430630189, 430630417, 430630436, 430630538, 430630573, 430630605, 430630612,  430630729, 430630734, 430630735, 430630997, 430630998, 433530695, 433530696,  433530697, 436020457, 436020458, 436020459, 436020460, 458650459, 458650551,  458650963, 473350707, 473350710, 473350711, 473350712, 493490116, 493490118,  493490142, 493490365, 493490395, 493490402, 493490705, 493490778, 493490802,  493490820, 493490907, 493490942, 493490993, 500901137, 500901138, 500901139,  500901676, 500901721, 500901741, 500901755, 500901962, 500902102, 500902203,  500902248, 500903389, 500903461, 500903752, 500903926, 500903931, 500903932,  500904614, 500905309, 500905853, 502680750, 502680751, 502680752, 502680753,  504360139, 504361220, 504369949, 504369950, 504369951, 504369952, 510790726,  510790727, 510790728, 516550032, 516550429, 516550606, 516550608, 516550609,  516550746, 516550848, 521250047, 521250061, 521250089, 521250463, 521250852,  521250876, 521250914, 529590441, 529590643, 529590994, 530021547, 532170274,  532170291, 532170315, 532170329, 538080921, 538080932, 538080967, 538081131,  538081132, 545696137, 545696138, 545696139, 548686014, 548686015, 548686016,  548686017, 551545371, 551545372, 551547142, 551547146, 557000210, 557000217,  557000226, 557000227, 557000228, 557000596, 557000691, 557000894, 557000995,  572370134, 572370135, 572370136, 572370137, 581180707, 581180710, 581180711,  581180712, 591150124, 591150125, 591150126, 591150127, 597621030, 597621031,  597621032, 597621033, 604290769, 604290770, 604290771, 604290772, 605052760,  605052761, 605052762, 605052763, 606870108, 607230031, 607230032, 607230033,  607600075, 607600094, 607600278, 607600279, 607600280, 607600287, 607600514,  607600577, 617860293, 617860298, 617860580, 617860611, 617860623, 617860668,  617860732, 617860736, 617860863, 617860996, 619190172, 619190185, 619190190,  619190212, 619190318, 619190369, 619190439, 619190679, 619190691, 619190817,  619190823, 619190824, 619190825, 619190883, 627560707, 627560710, 627560711,  627560712, 631870059, 631870060, 631870077, 631870118, 631870228, 631870230,  631870283, 631870479, 631870696, 631870758, 631870773, 631870801, 631870963,  633040778, 633040779, 633040780, 636293294, 636293994, 636293995, 636294947,  636296431, 636296871, 636298189, 647250707, 647250710, 658410647, 658410648,  658410649, 658410650, 658410651, 658410652, 658620171, 658620172, 658620173,  658620174, 680711612, 680711900, 680711960, 680711971, 680713012, 680713088,  680713196, 680714756, 680714760, 680714793, 680714877, 680715125, 680715165,  680840342, 680840343, 680840344, 680840345, 681800170, 681800171, 681800172,  681800173, 682583000, 682583001, 682583002, 682587056, 682587156, 682587159,  683820004, 683820005, 683820138, 683820139, 683820140, 683820141, 683820769,  683820863, 683820864, 683870558, 683870559, 683870560, 684620108, 684620109,  684620110, 684620153, 684620370, 684620371, 684620372, 684620373, 684620374,  687886366, 687886377, 687886417, 687886435, 687886770, 687886824, 687886916,  687887010, 687887016, 687887351, 687887460, 687887468, 687887568, 687888965,  687888967, 687889499, 687889532, 687889533, 687889730, 690970122, 690970123,  690970124, 690970125, 690970816, 690970817, 690970818, 690970819, 705180233,  705180333, 705180344, 705180370, 705180420, 705180517, 705180625, 705180887,  705181180, 705181196, 705181503, 705181651, 705181674, 705181718, 705181853,  705181937, 705181953, 705182001, 705182104, 705182322, 705182391, 705182419,  705182498, 705182517, 705182899, 705182955, 705183067, 705183460, 705183758,  707101039, 707101040, 707101041, 707101042, 707101043, 707711315, 707711316,  707711317, 707711656, 707711657, 707711658, 707711659, 707711660, 709340022,  709340133, 709340149, 709340420, 709340450, 709340606, 709340733, 709340748,  709340768, 709340772, 712050187, 712050195, 712050202, 712050214, 712050233,  712050819, 713350047, 713350325, 713350337, 713350359, 713350487, 713350494,  713350499, 713350538, 713350548, 713350925, 713351064, 713351116, 713351143,  713351147, 713351684, 713359727, 716100193, 716100485, 716100486, 716100492,  716100493, 726030120, 726030121, 726030122, 726030123, 726030124, 727890004,  762820278, 762820279, 762820280, 762820281, 764200276, 764200278, 764200279,  764200280, 804250092, 804250195, 804250202, 804250208, 804250214, 804250288,  820090135, 820090136, 820090137, 830080011, 830080012, 216950128, 216950129,  216950130, 504580639, 504580640, 504580641, 504580642, 504580645, 504580647,  548684672, 548684674, 548685190, 548685343, 552890433, 619190189, 636293321,  705181393, 705182165, 705182753, 526529001, 002451071, 002451072, 002451073,  002451074, 002451075, 177720101, 177720102, 177720103, 177720104 |
|  | MMSL Synonym | 205293, 18525, 19089, 353803, 343382, 205295, 19088, 162273, 19087, 205296, 213505, 343383, 13669, 49828, 192126, 343379, 205294, 192128, 19090, 343576, 205302, 343380, 192129, 192130, 192131, 65036, 65035, 49829, 6438, 16508, 17041, 17040, 13957, 170342, 170344, 170343, 170345, 170341, 353814, 353813, 343382, 213511, 343383, 213510, 213507, 343379, 213506, 213509, 213508, 343576, 343380, 205299, 205297, 205298, 205300, 205301 |
|  | MMSL Drug ID | d04115, d07881 |
| naltrexone (Vivitrol, Revia, Depade) | NDC | 001850039, 004061170, 005550902, 009047036, 167290081, 422910632, 430630469,  430630591, 473350326, 500902866, 500903076, 500903929, 500904925, 504360105,  512240206, 521250727, 532170261, 548685574, 621350242, 636291046, 636291047,  636295304, 680712156, 680712721, 680840291, 680940853, 687887084, 691890499,  693643143, 705181146, 705181312, 705182718, 713350014, 713351480, 713352062,  721621566, 765191160, 657570300, 657570301, 512850275, 56001122, 56001130, 56001170, 56007950, 56008050, 185003901, 185003930, 406009201, 406009203, 406117001, 406117003, 555090201, 555090202, 16729008101, 16729008110, 42291063230, 43063059115, 47335032683, 47335032688, 50090286600, 50436010501, 51224020630, 51224020650, 51285027501, 51285027502, 52152010502, 52152010504, 52152010530, 54868557400, 63459030042, 63629104601, 63629104701, 65694010003, 65694010010, 65757030001, 65757030202, 68084029111, 68084029121, 68094085362, 68115068030 |
|  | MMSL Synonym | 167360, 12838, 72424, 228674, 18420, 167361, 167363, 167359, 167323, 81542, 11621, 136490, 136489, 228676, 187249, 167362, 137294, 244063, 167364, 81544, 81543, 1157, 15708 |
|  | MMSL Drug ID | d01406, d07135, d07472 |
|  | HCPCS | G0723, J2315 |
|  | ICD-10 Procedure | HZ84ZZZ, HZ94ZZZ |

**Table 13c.** List of codes used to define medications for tobacco use disorder (MTUD), psychotropic and general SUD

| MTUD | Medications | NDC | 00024581030, 00024581130, 00069046856, 00069046903, 00069046912, 00069046956, 00069046997, 00069047102, 00069047103, 00069047197, 00093081001, 00093081005, 00093081101, 00093081105, 00093081201, 00093081205, 00093081301, 00093570301, 00115544513, 00115681102, 00115681108, 00115681110, 00121067816, 00173013555, 00173017755, 00173017855, 00173055601, 00173055602, 00173072200, 00173073001, 00173094755, 00185041001, 00185041005, 00185041060, 00185041501, 00185041505, 00185041560, 00185111160, 00187073030, 00187073090, 00187073130, 00187581030, 00187581130, 00187581230, 00378043301, 00378043305, 00378043501, 00378043505, 00378200905, 00378552101, 00406991003, 00406991103, 00406991203, 00406991303, 00527241532, 00527243032, 00591333105, 00591333119, 00591333130, 00591333205, 00591333230, 00591354005, 00591354060, 00591354105, 00591354160, 00591354260, 00591354360, 00781105301, 00781106401, 00781516960, 00781552810, 00781552910, 00904657304, 00904658561, 00904663561, 00904663661, 00904708404, 00904708461, 10370010103, 10370010150, 10370010203, 10370010250, 16571086250, 16590024630, 16729044310, 16729044315, 16729044316, 16729044410, 16729044416, 21695063356, 23155019101, 23155019201, 23490923006, 23490923103, 23490923203, 24689011901, 24979010207, 35356008730, 35356008760, 35356036960, 42806034809, 42806034905, 42806034909, 42806041501, 43547028810, 43547028910, 43547028950, 43547029010, 43598065530, 43598075105, 43598075160, 43598075201, 43598075360, 43598086360, 45963014205, 45963014290, 47781063730, 49884015576, 49884015676, 49884094499, 49909001030, 50268014013, 50268060315, 50268060415, 51079004720, 51079094320, 51079094420, 51407020605, 51672400101, 51672400201, 51672400205, 51672400305, 51672400401, 51862001505, 51862001601, 51862001605, 51862001610, 51862001705, 51862001801, 51862094505, 51862094601, 51862094605, 51862094705, 51862094801, 52427057530, 52959028530, 52959086930, 54868476300, 54868566400, 54868567400, 55289090015, 55289090030, 57866308305, 58016059990, 60505015701, 60505015801, 60505476505, 60505476606, 60687028101, 60687029301, 60687034001, 60687035101, 64679083001, 64980020101, 67767013305, 67767014190, 68001030800, 68001032203, 68001052004, 68084025221, 68084069701, 68084070825, 68180031902, 68180031906, 68180031909, 68180032002, 68180032006, 68180032009, 68382035305, 69097087512, 69097087712, 69097087812, 69097087907, 69097091707, 69097091807, 70436001002, 70436001004, 70436001006, 70436001102, 70436001104, 70436005901, 70436005902, 70436005922, 76282048130 |
| --- | --- | --- | --- |
|  |  | MMSL Drug ID | d00144, d00181, d05807, d08288 |
|  |  | MMSL Synonym | 2738, 4319, 6125, 7531, 7866, 10183, 10184, 12079, 12112, 16938, 16939, 16940, 16941, 17734, 18232, 18272, 18770, 20202, 20461, 20462, 20463, 20464, 23091, 25304, 25642, 25643, 26869, 26870, 27437, 31548, 42347, 42568, 43167, 44154, 44267, 47392, 47393, 47394, 47395, 47396, 47397, 47868, 82164, 82169, 82170, 82172, 82173, 82174, 82175, 82176, 110992, 147258, 147259, 162120, 162121, 162122, 162123, 162131, 166175, 166176, 186279, 186280, 188113, 188114, 193448, 193454, 193455, 198045, 198046, 198047, 203130, 203204, 209329, 209339, 209350, 209391, 227948, 228674, 228676, 312036, 317136, 328261, 329425, 329426,  329427, 351831, 351951, 351952, 361181, 361182, 581650, 591622, 636230, 636671, 636674, 636676, 637188, 637190, 749289, 749788, 794222, 795735, 795737, 835486, 993503, 993511, 993518, 993524, 993528, 993536, 993537, 993541, 993542, 993545, 993552, 993557, 993559, 993564, 993569, 993683, 993687, 993688, 993691, 993693, 993954, 1232585, 1232591, 1551467, 1551474, 1801289, 2572371, 2611266 |
|  | Procedures | ICD-10 | HZ80ZZZ, HZ87ZZZ, HZ90ZZZ, HZ97ZZZ |
| Psychotropic SUD | Procedures | ICD-10 | HZ88ZZZ, HZ98ZZZ |
| General SUD | Procedures | ICD-10 | HZ89ZZZ, HZ99ZZZ |

**Supplemental Figures 1A-1L.** Sample of characteristic distributional balance between individual treatments, summation of treatments, and combination of treatments in general SUD population


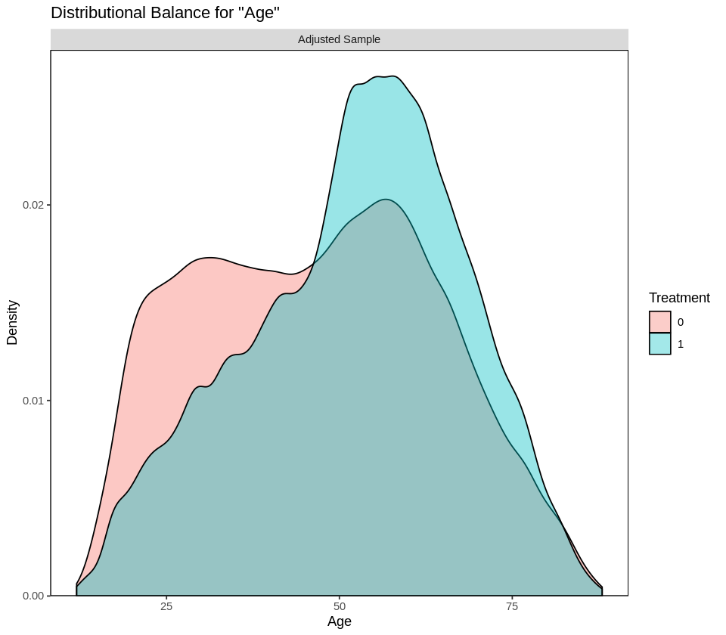

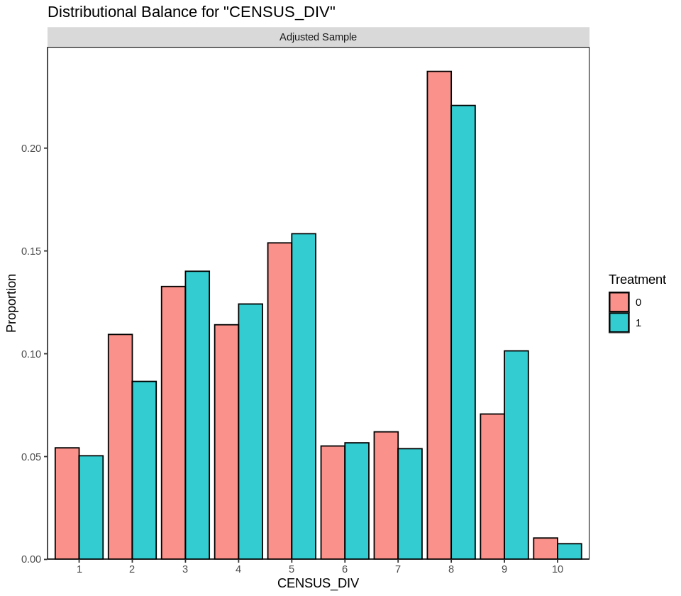


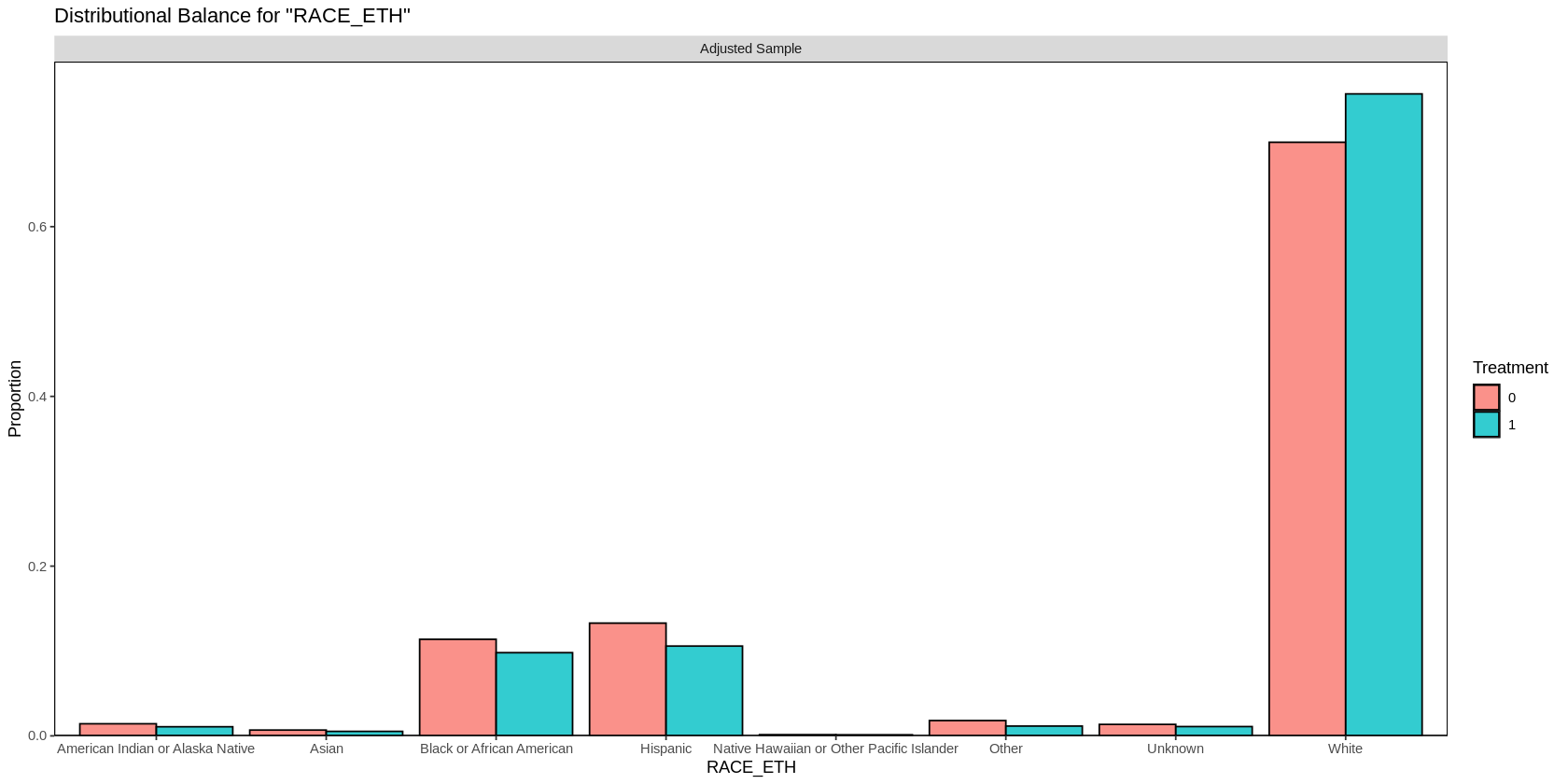


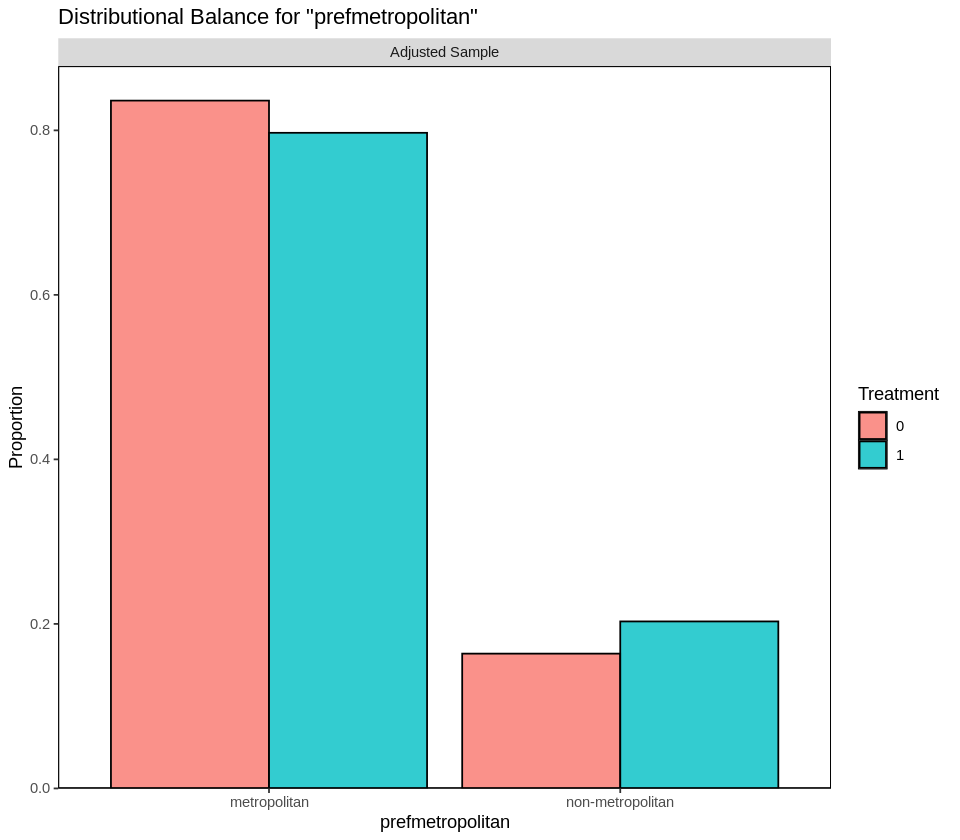

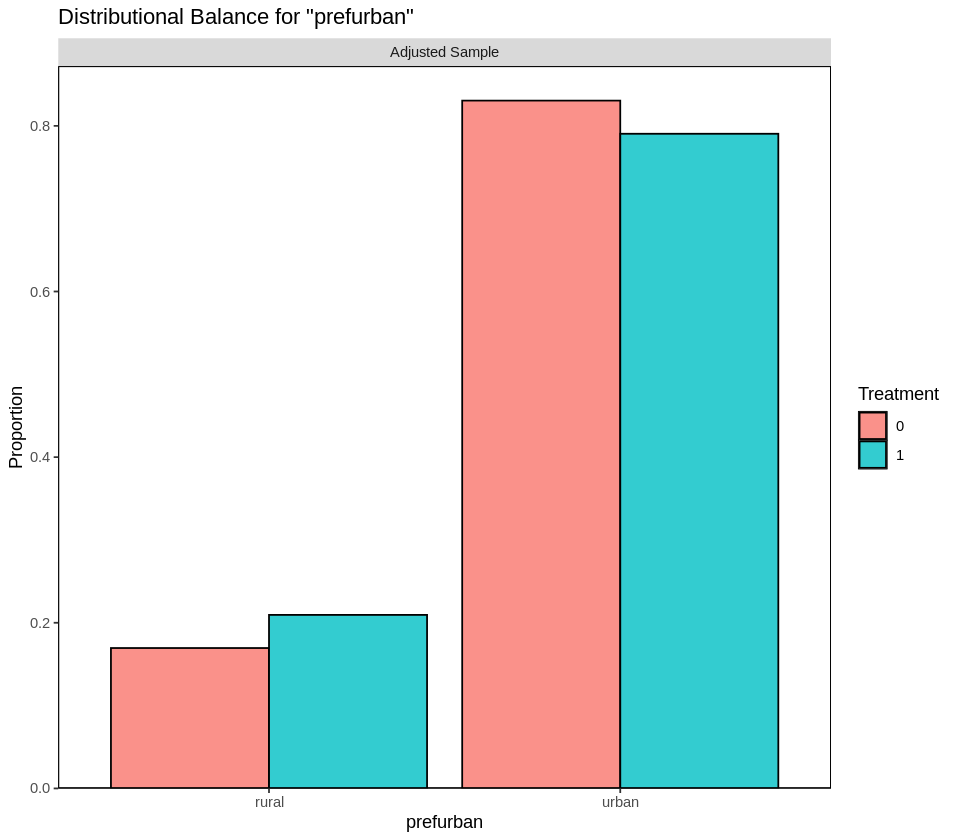


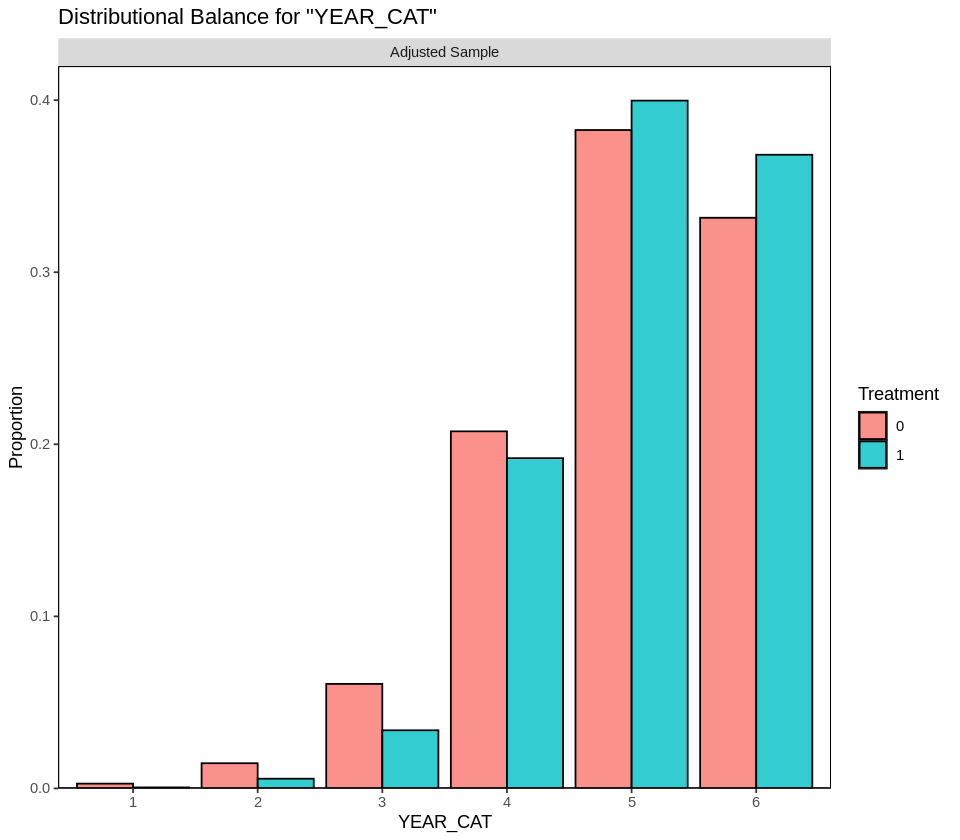

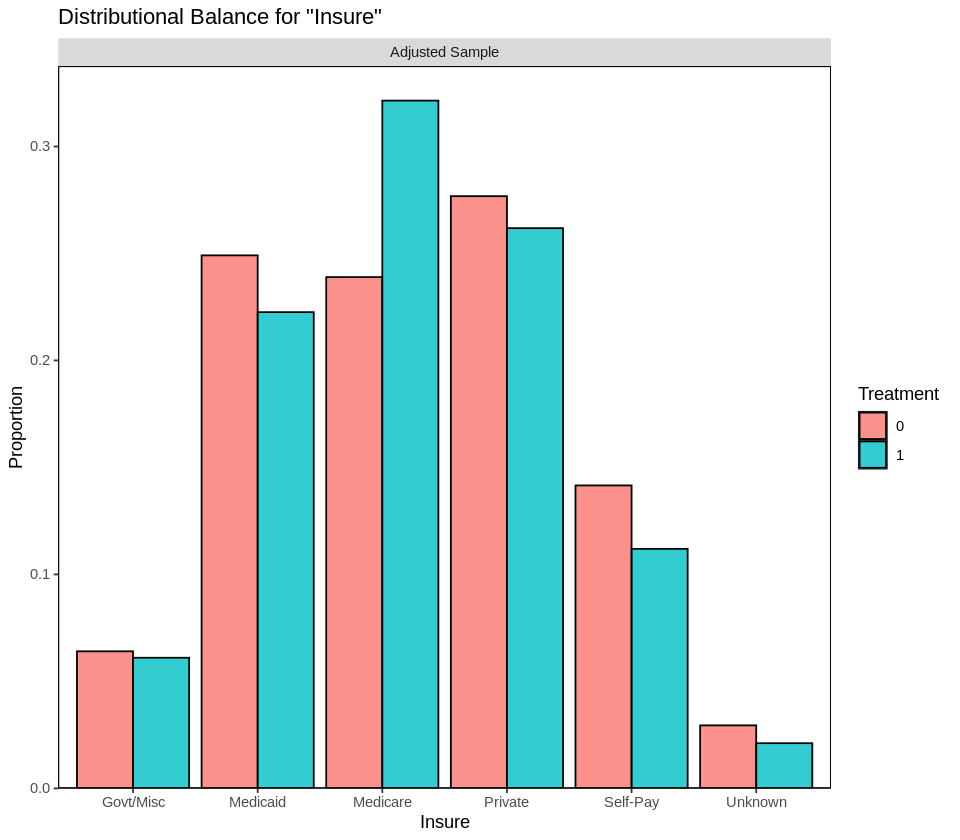


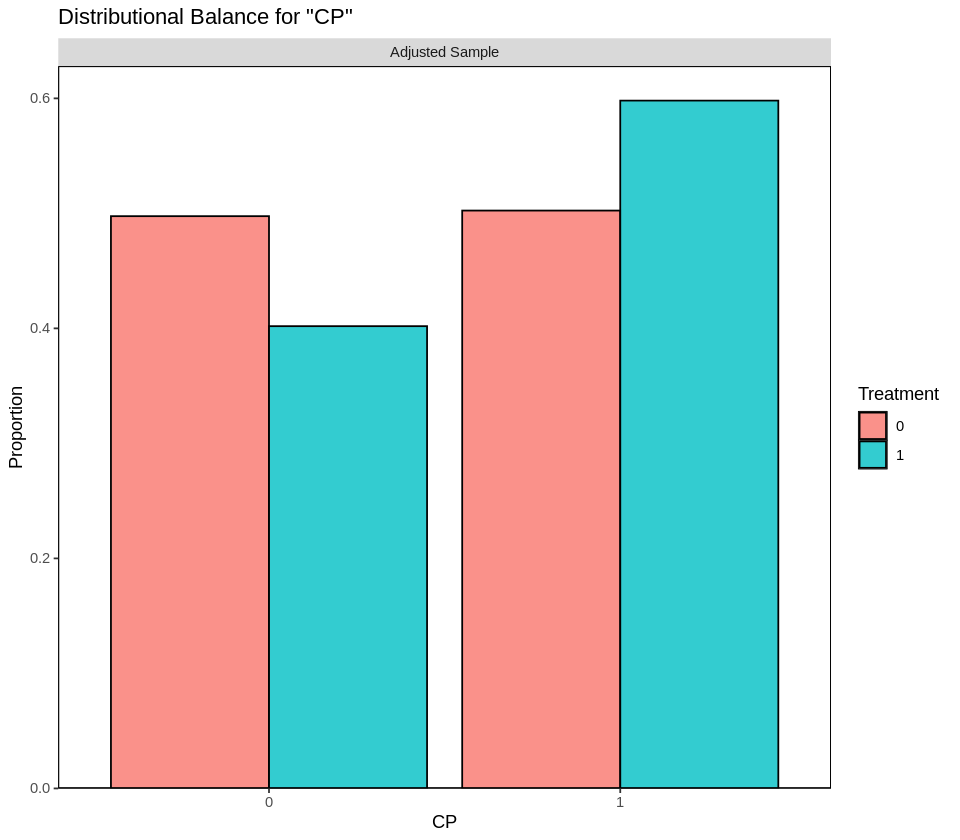

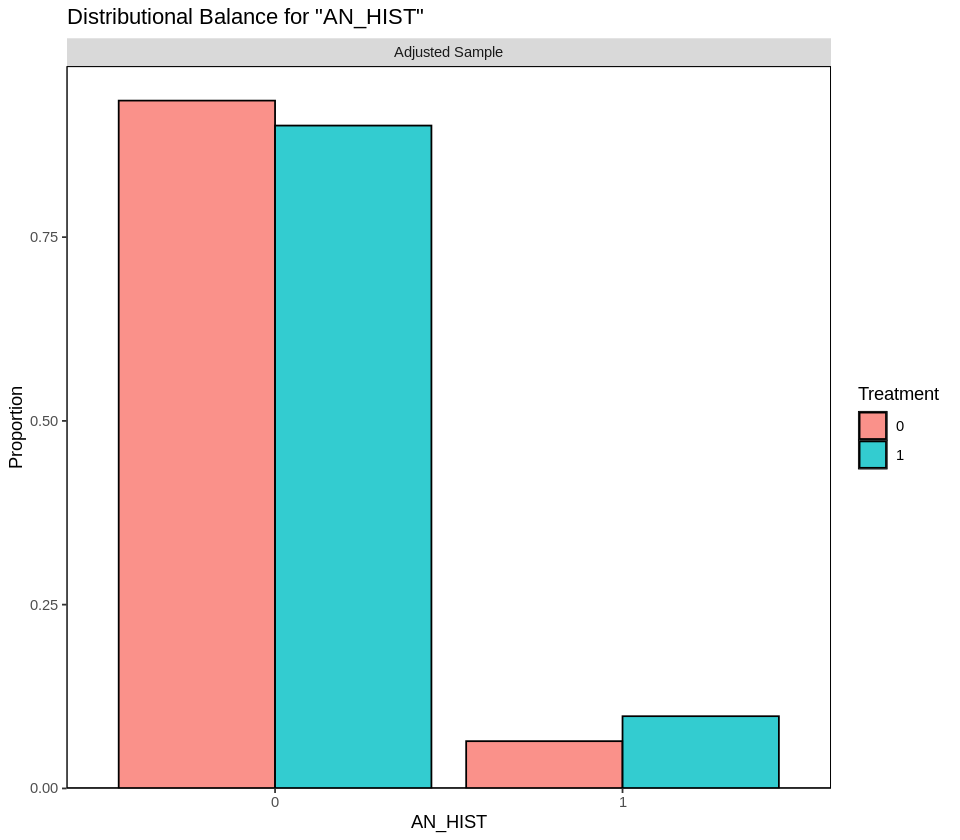


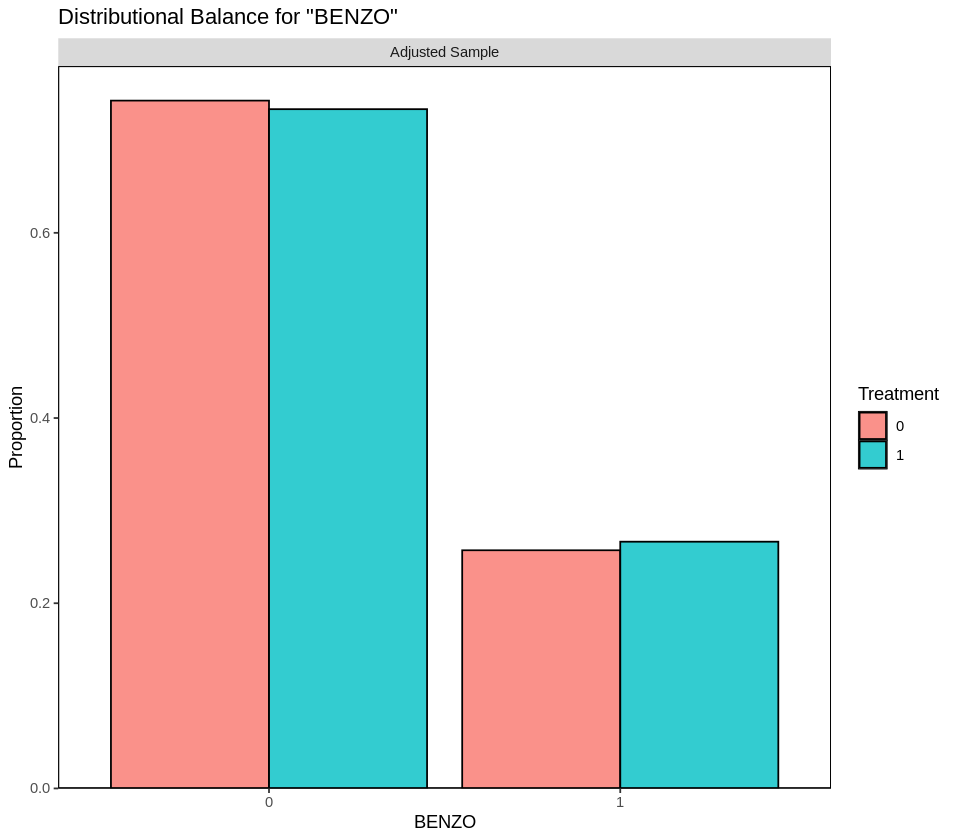

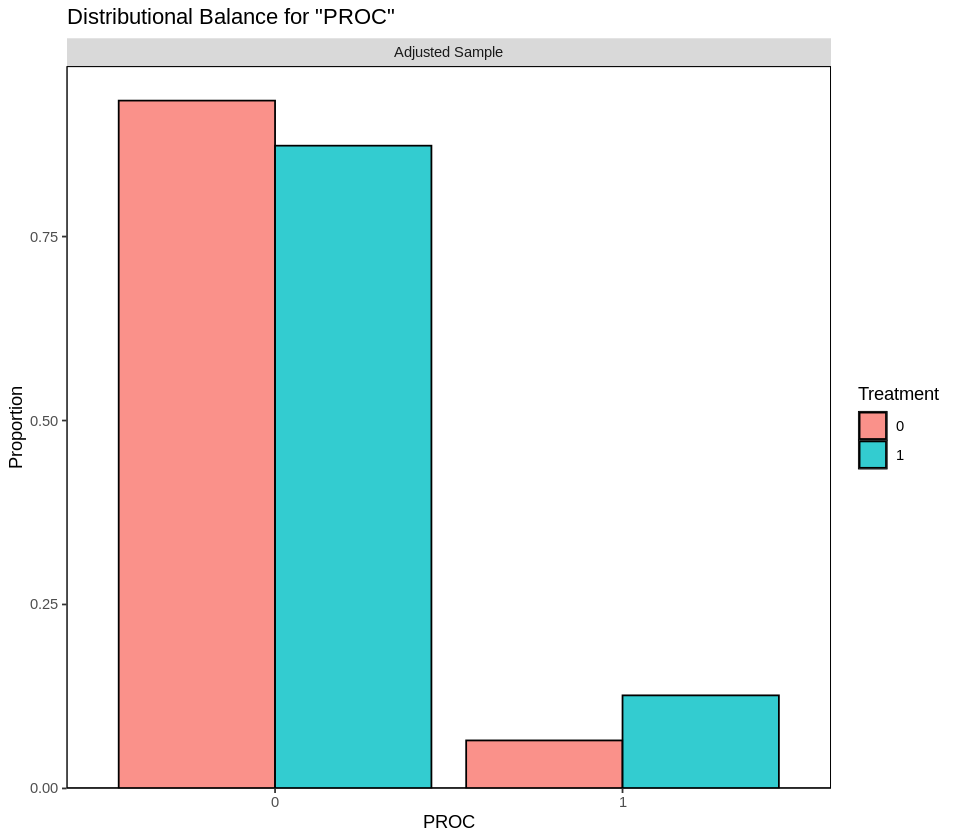


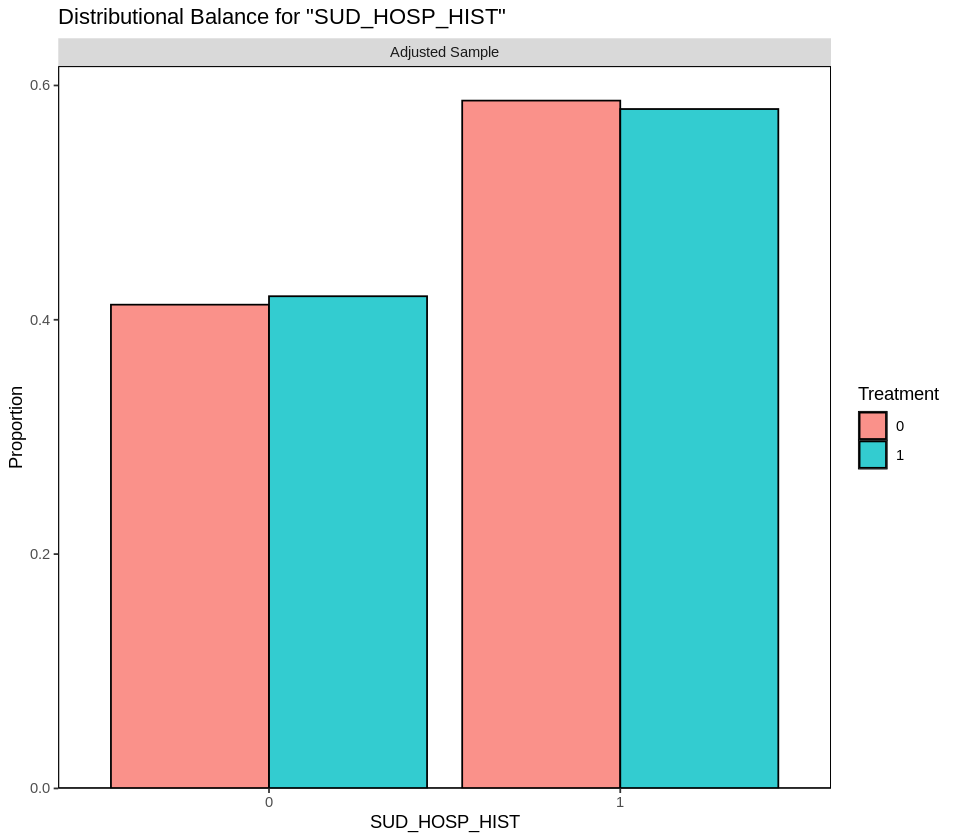


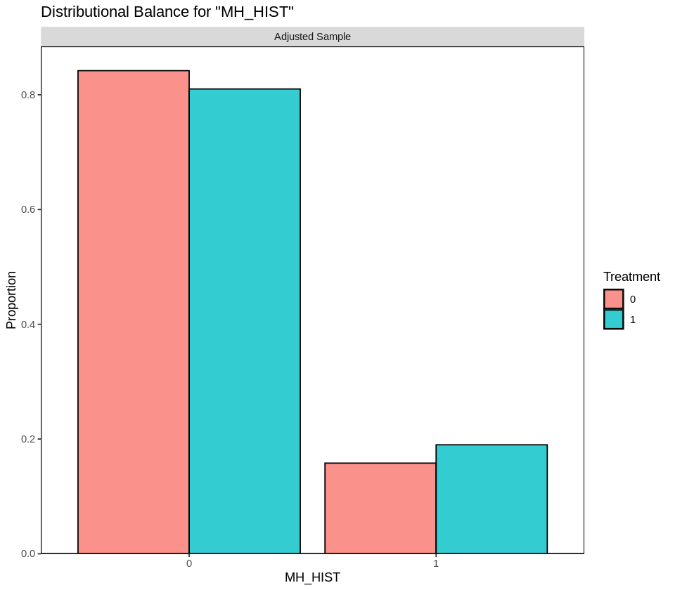

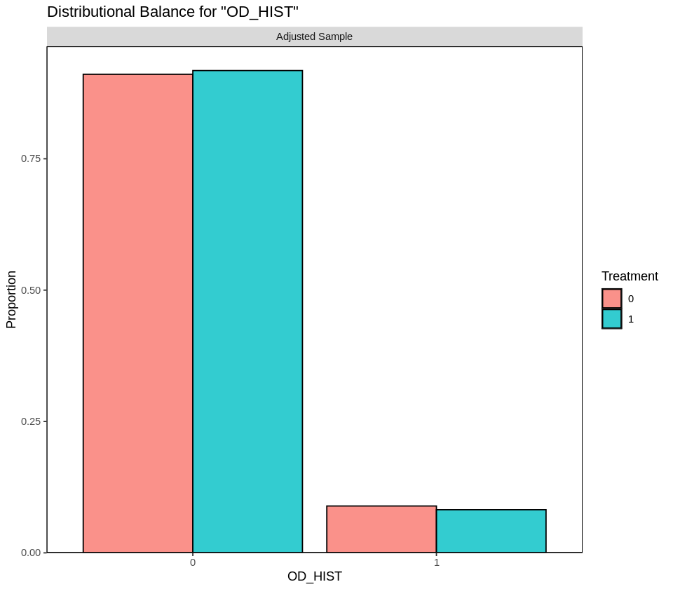


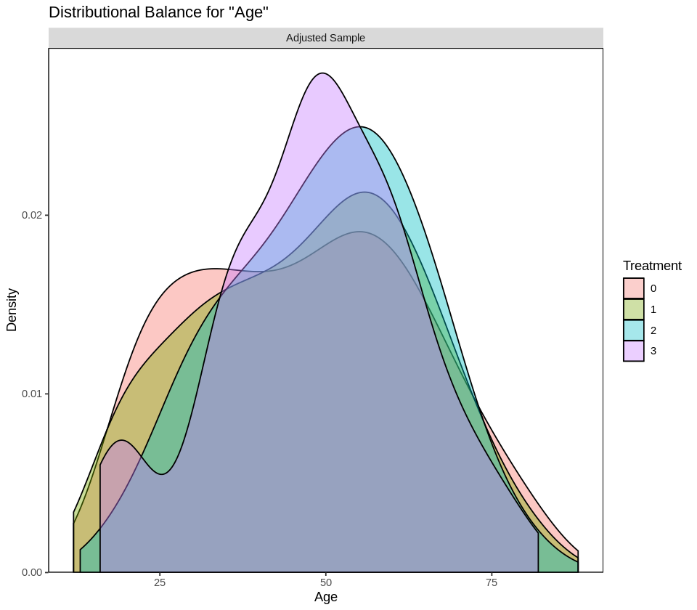

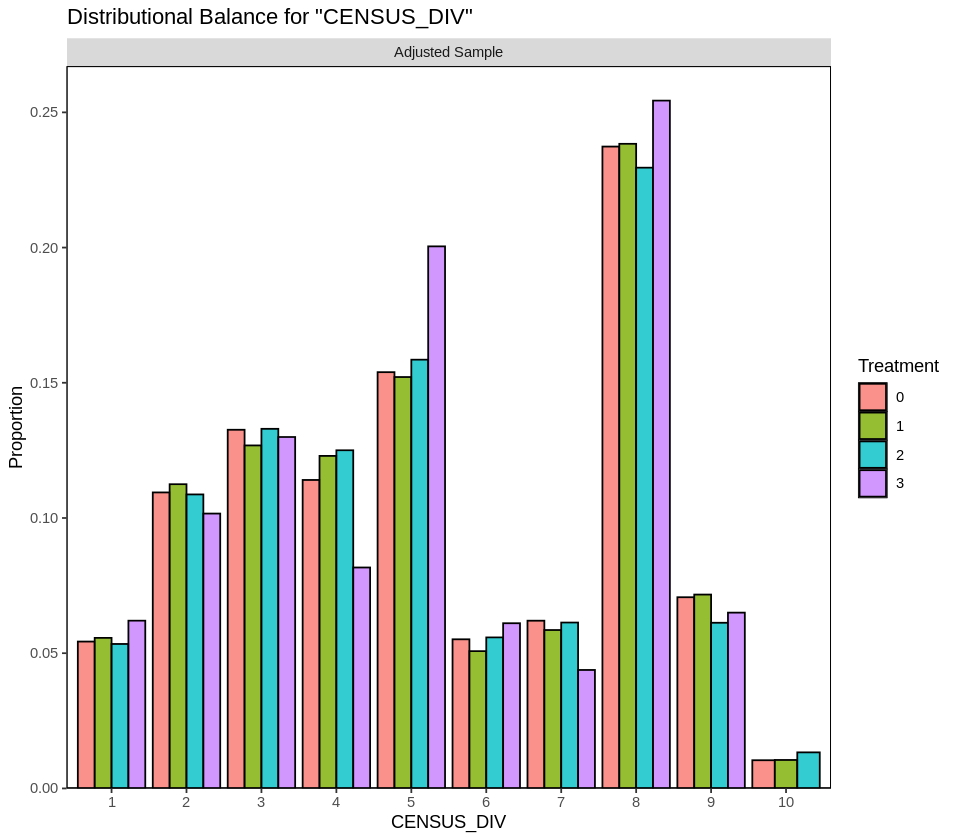


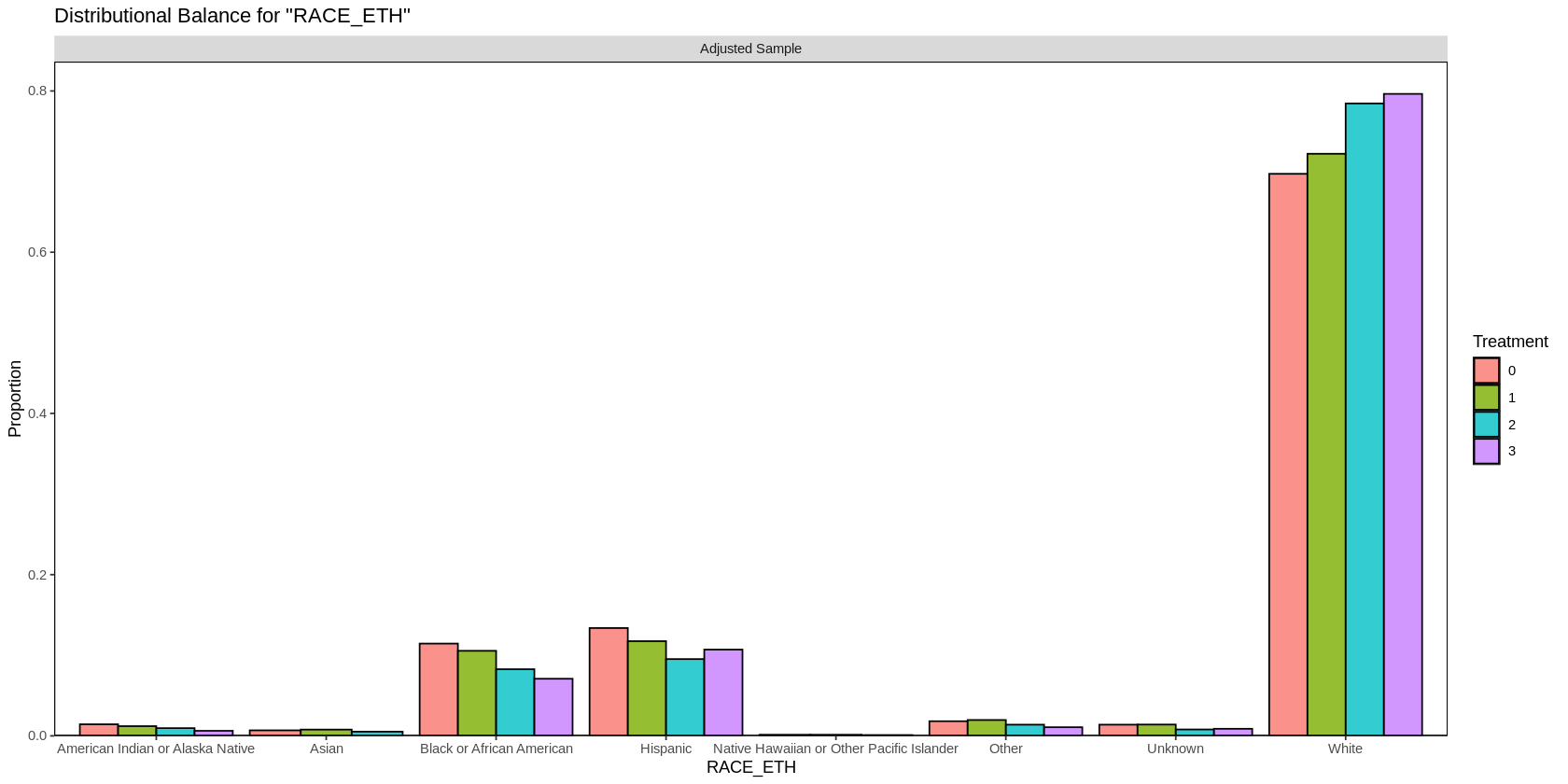


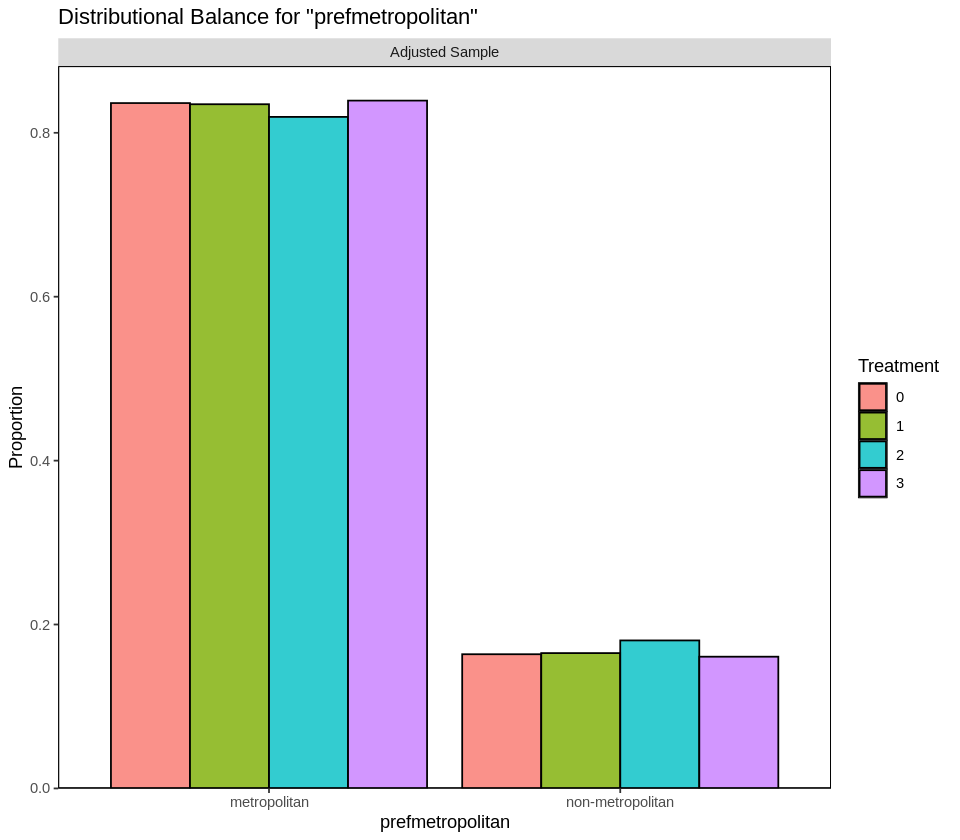

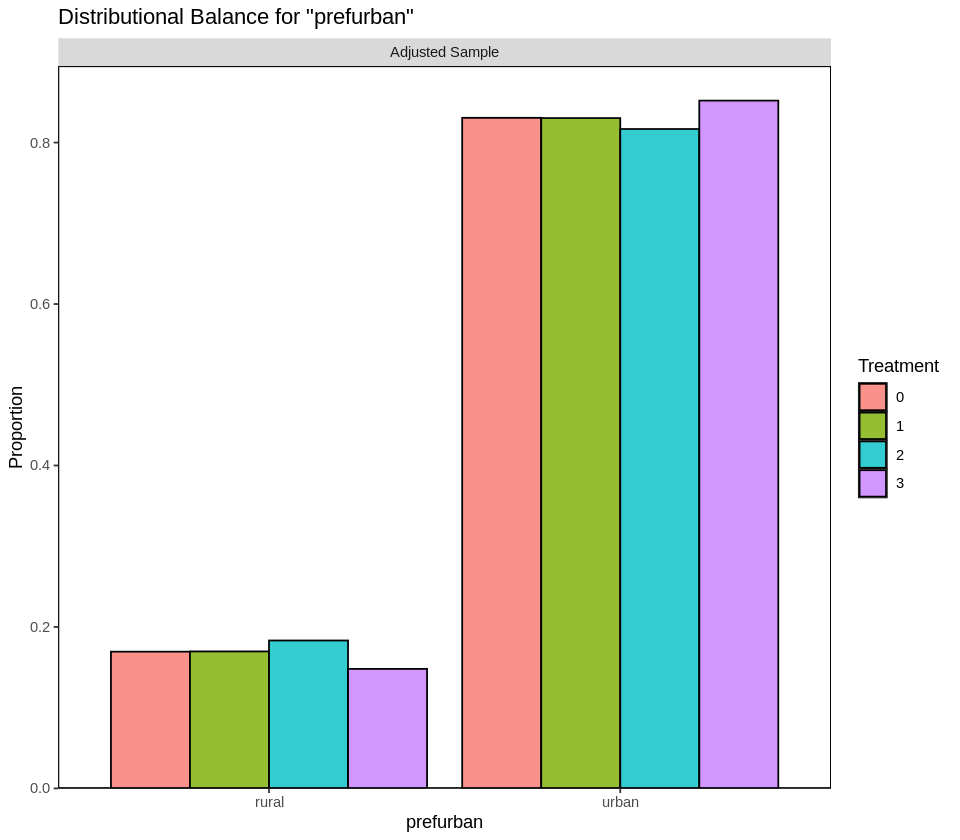


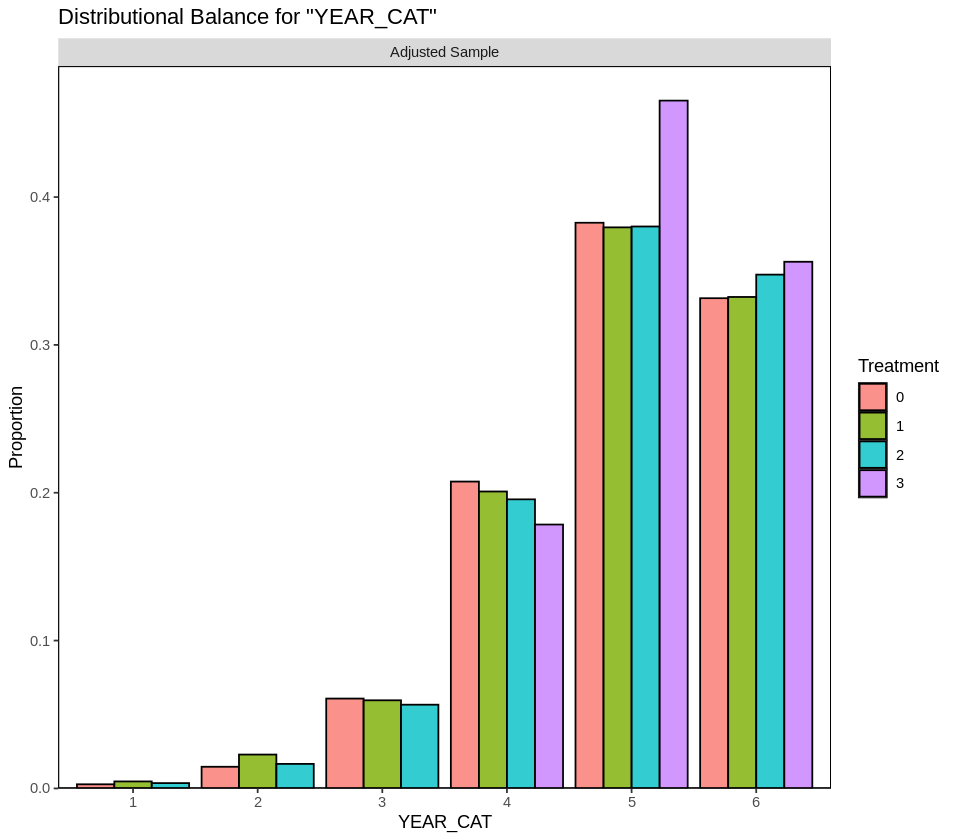

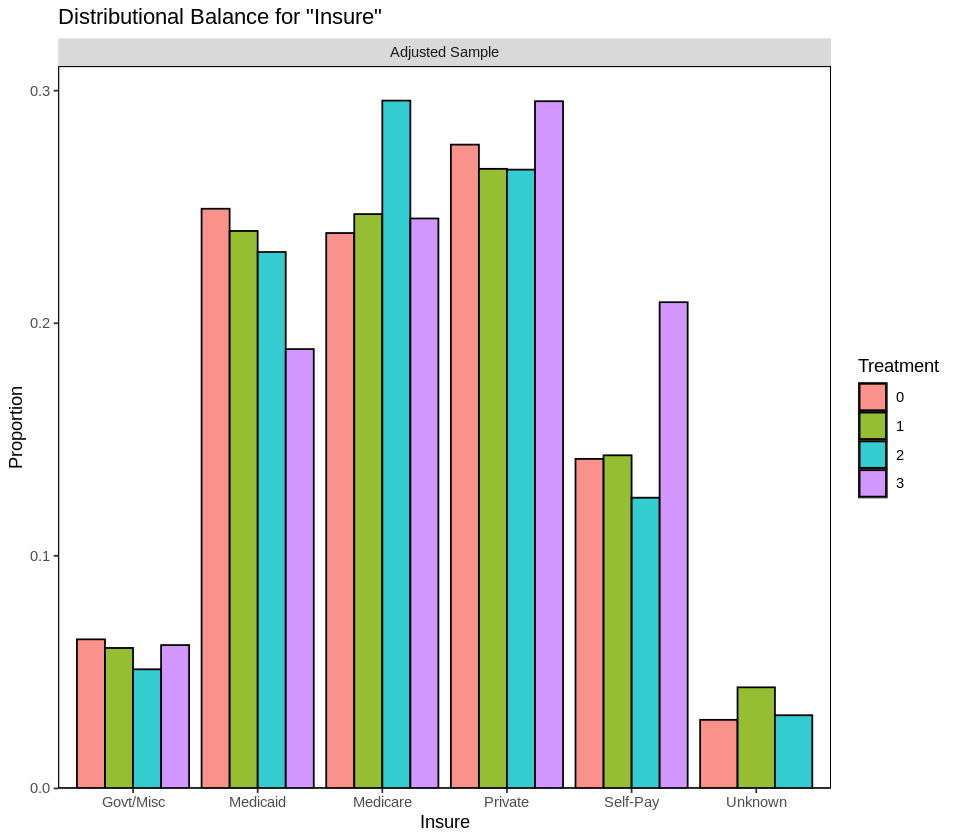


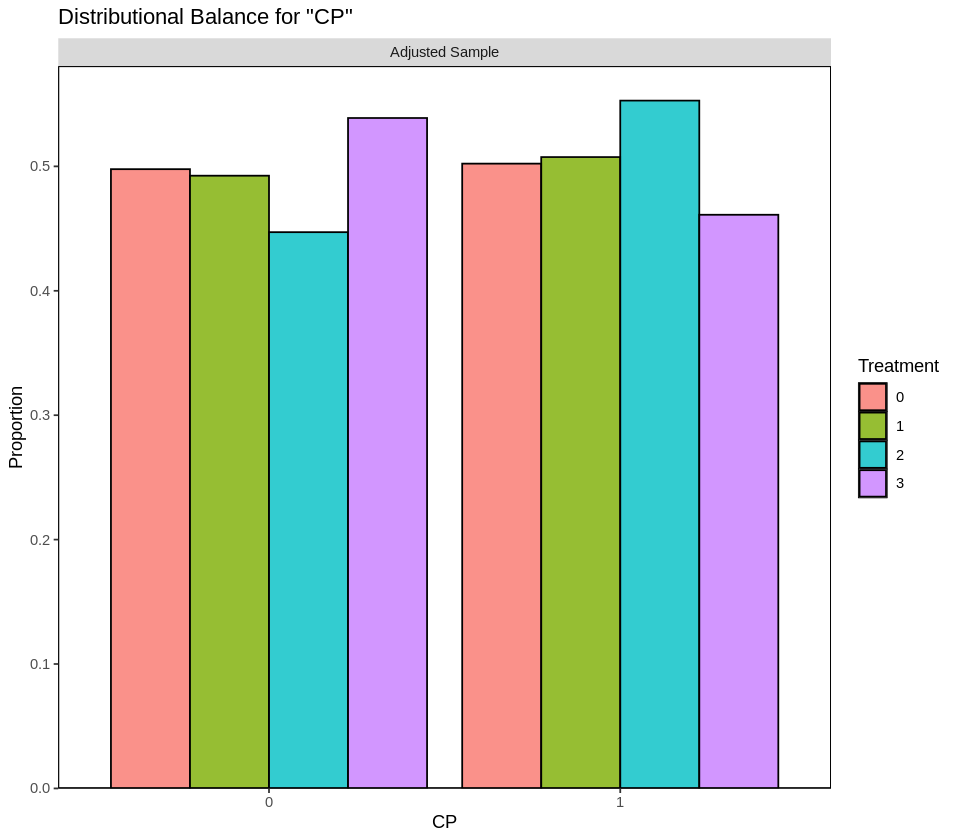

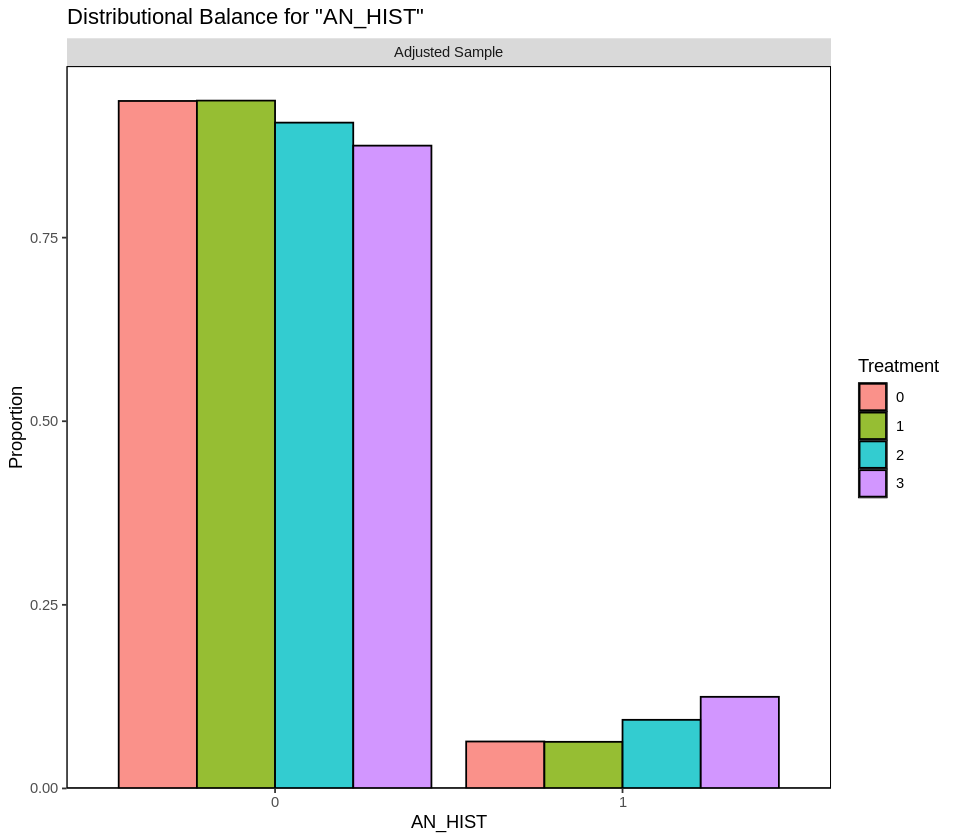


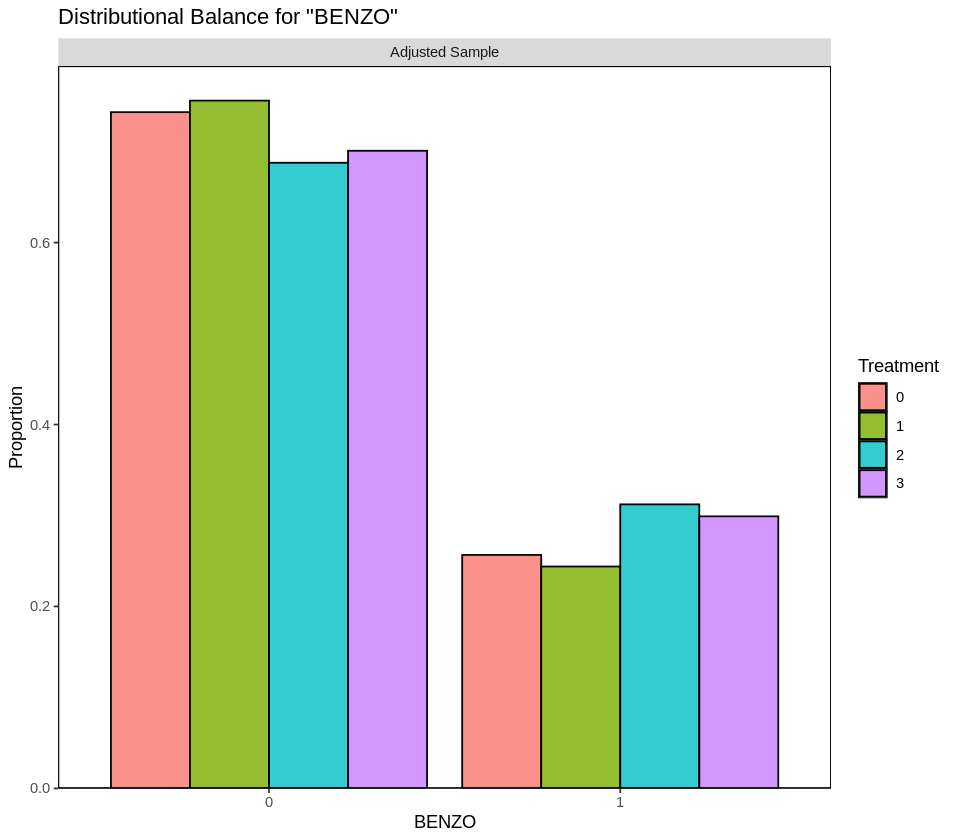

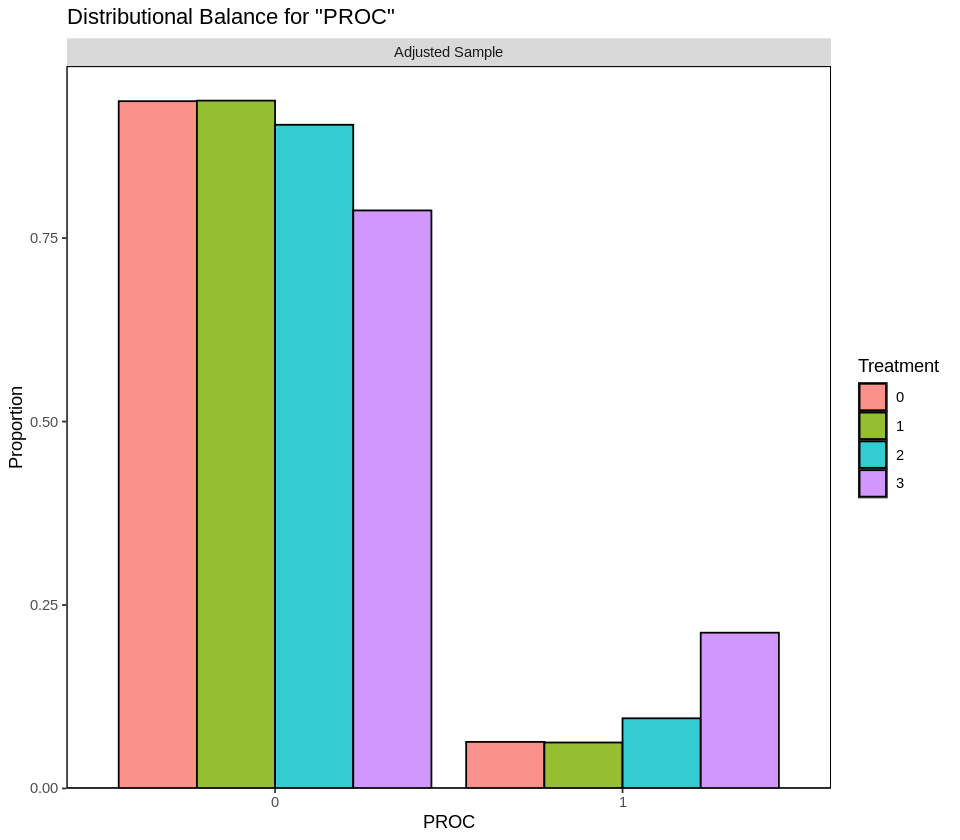


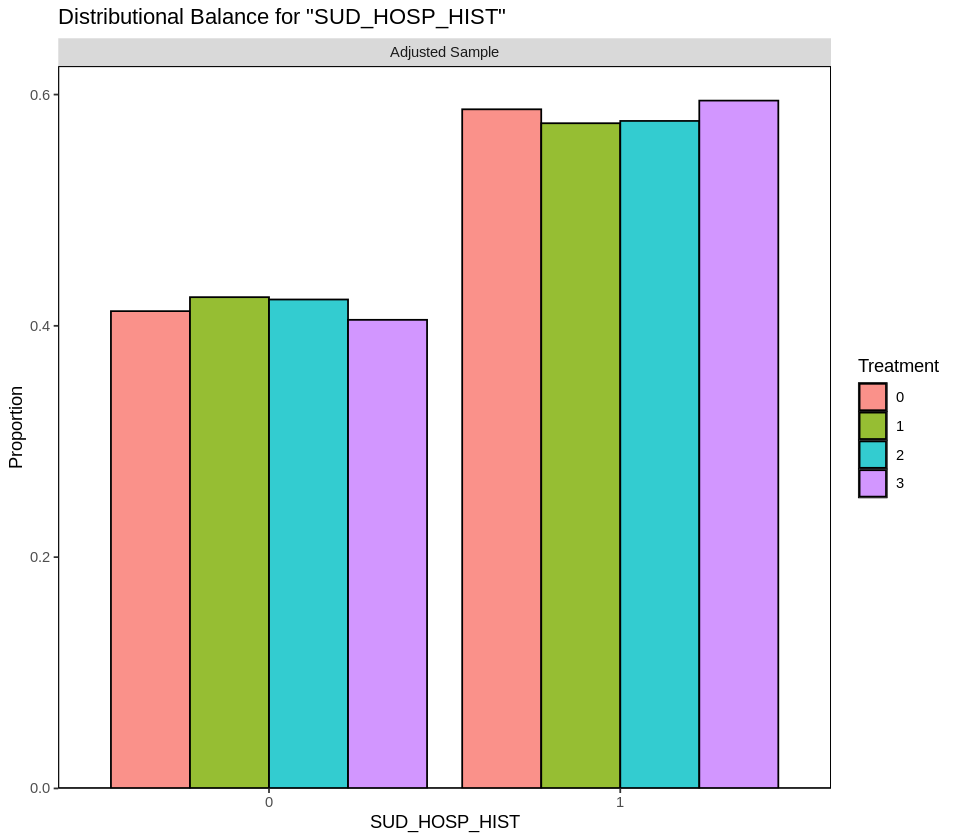


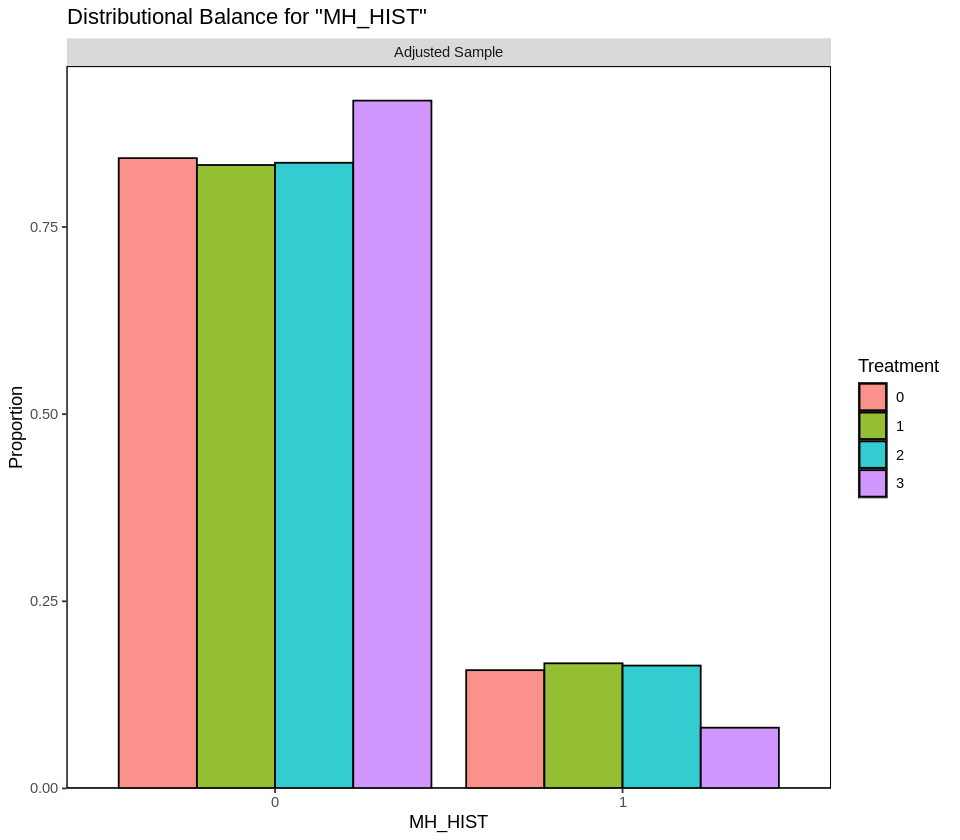

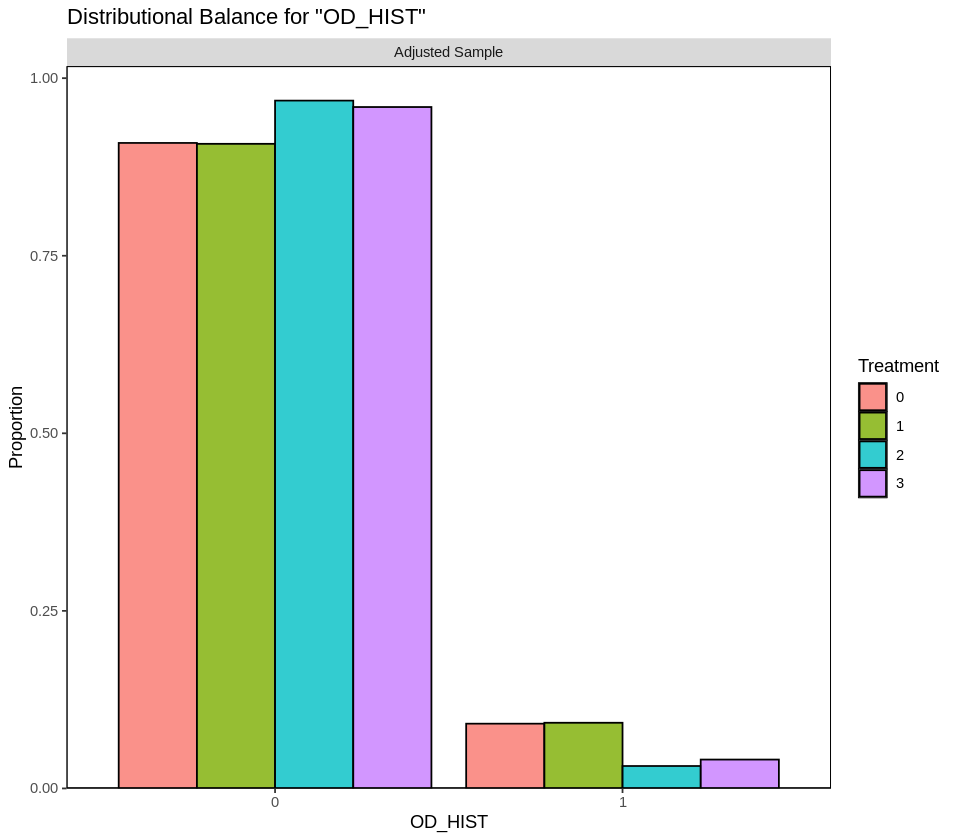


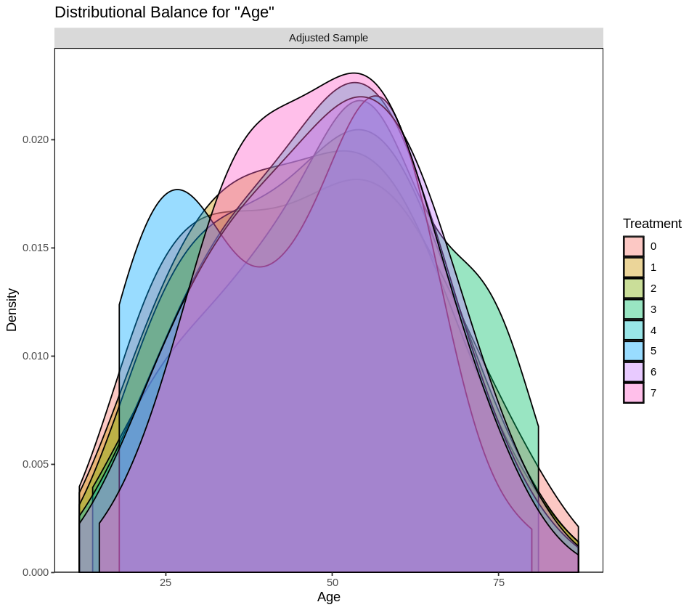

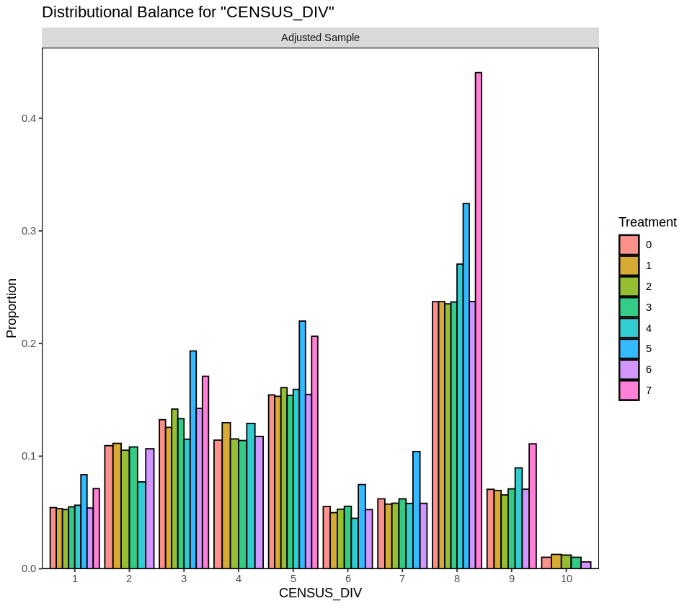


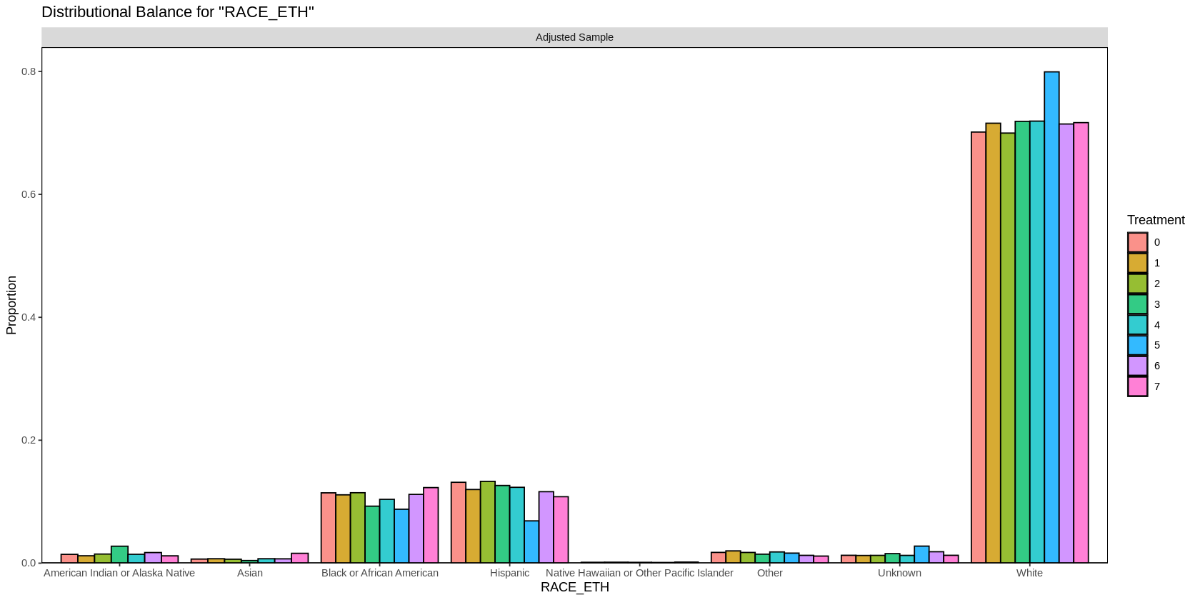


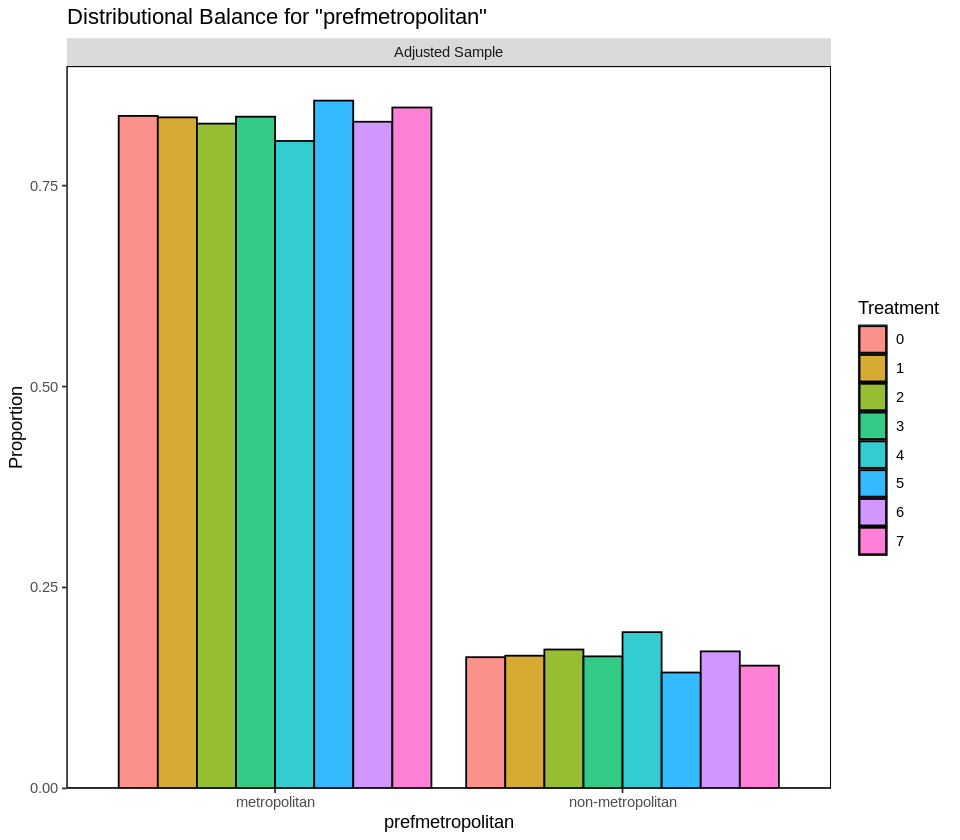

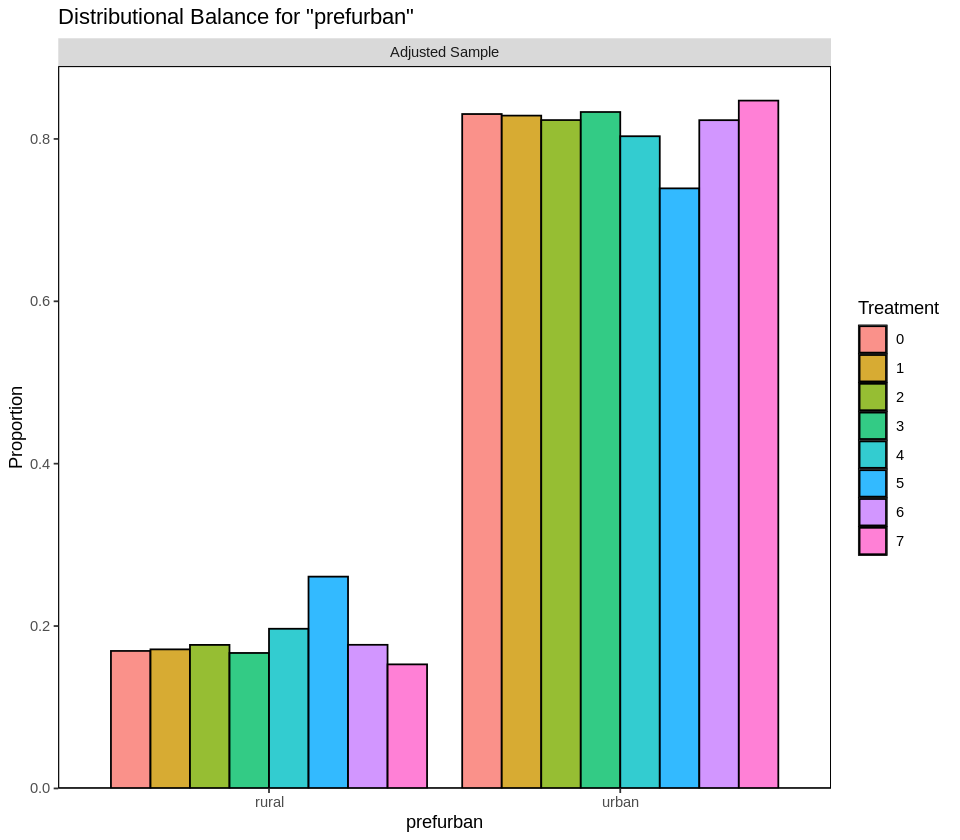


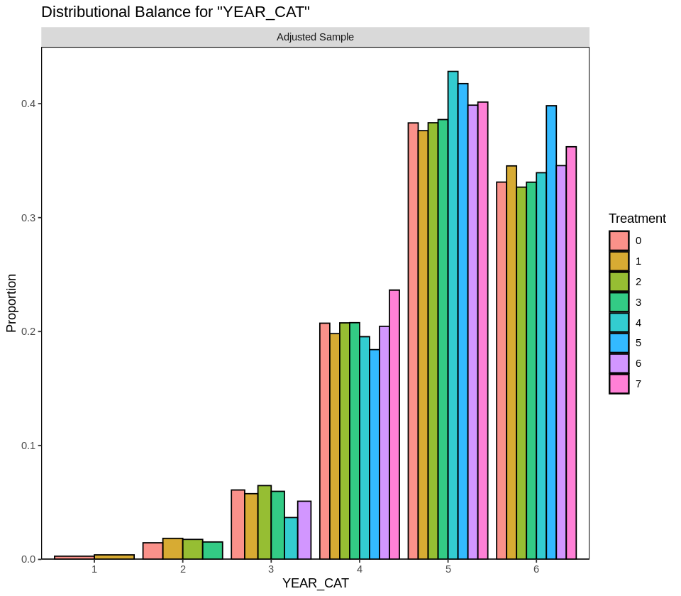

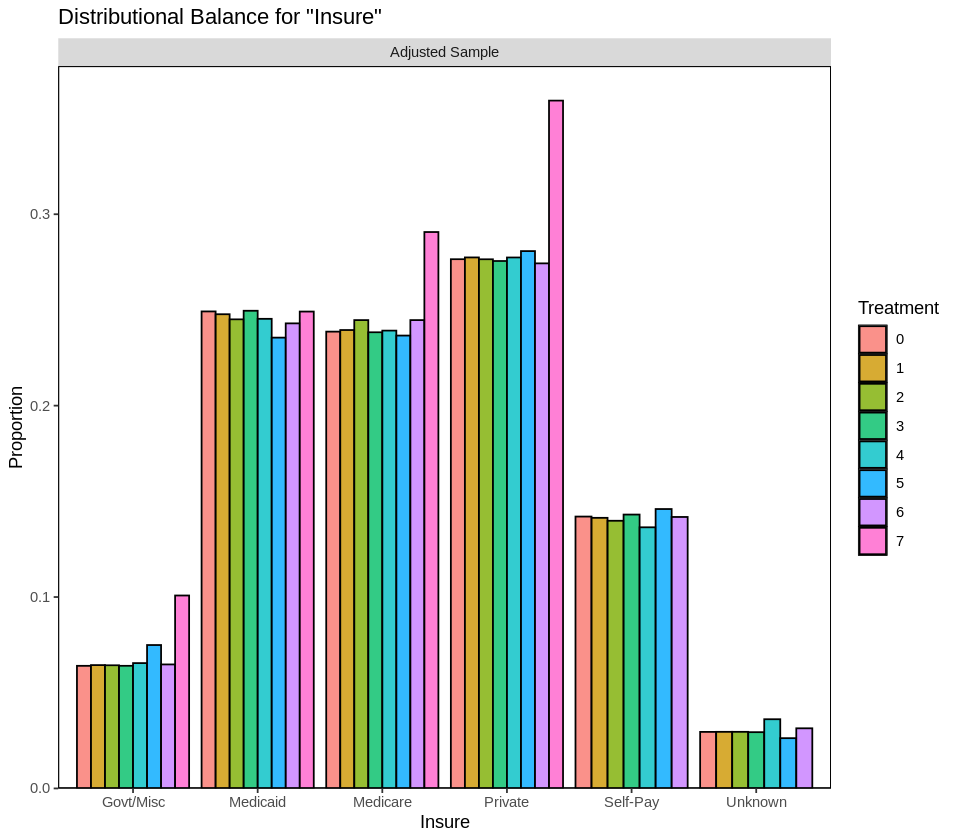


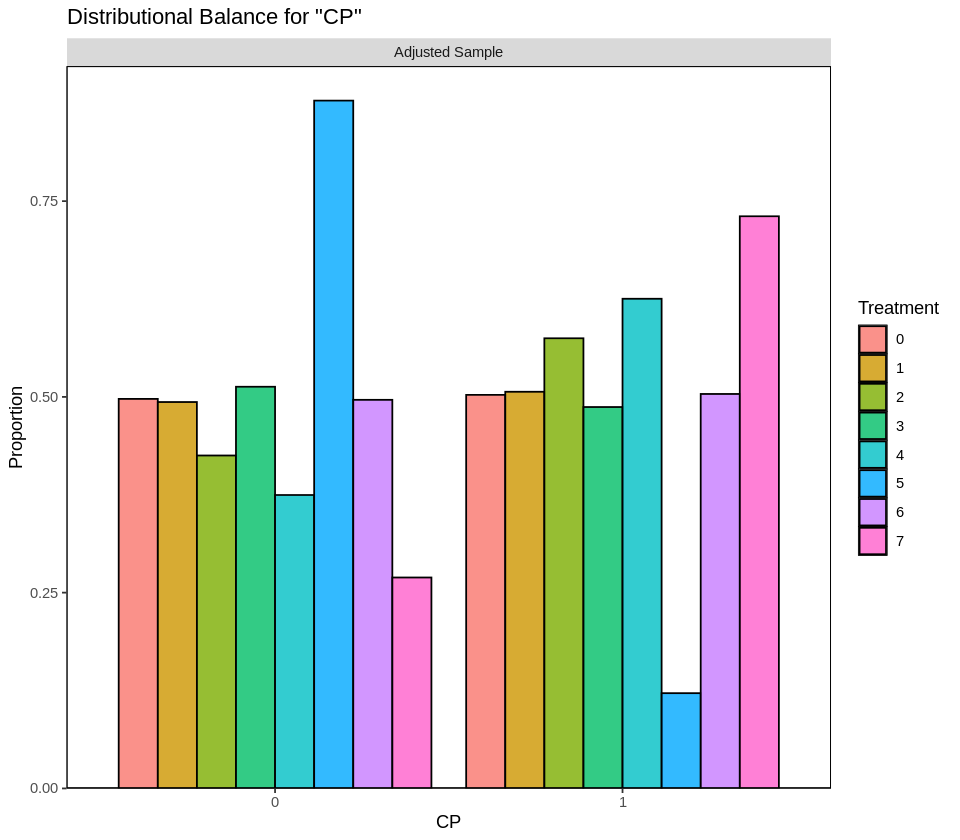

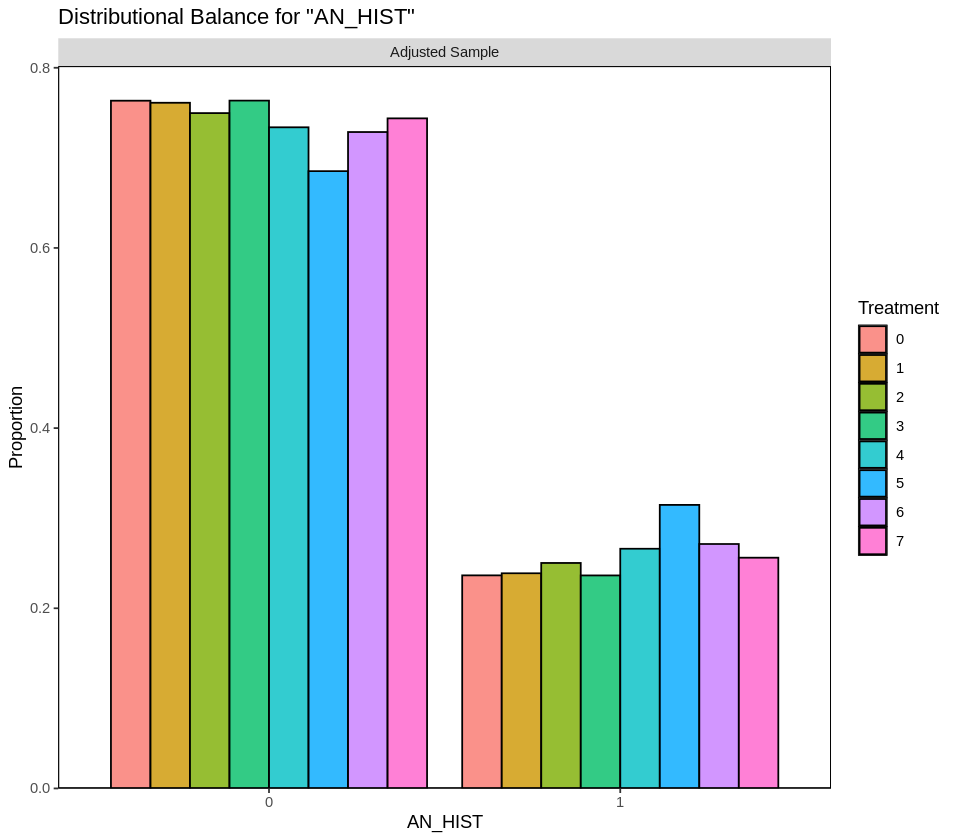


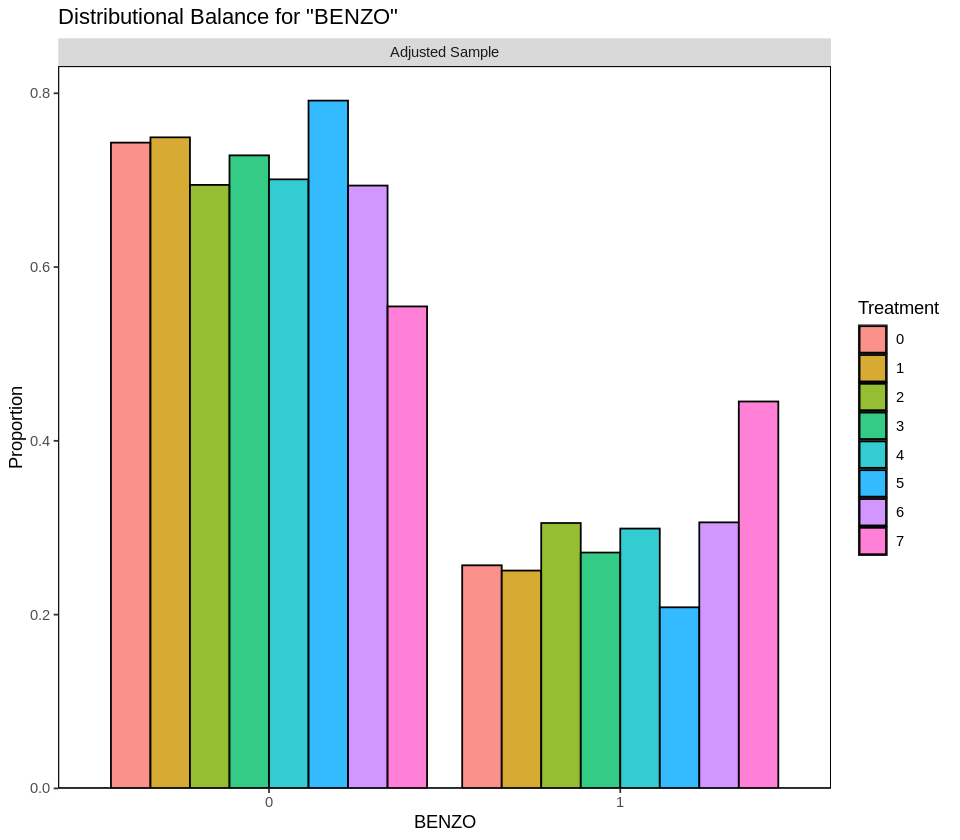

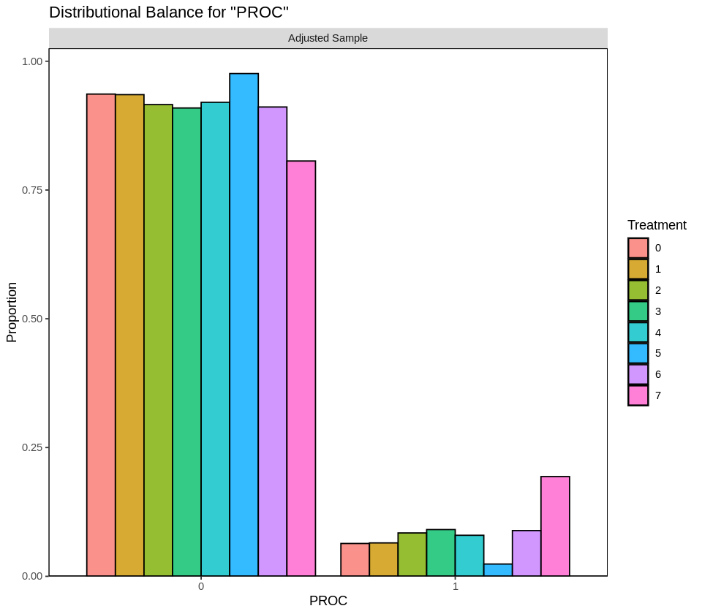


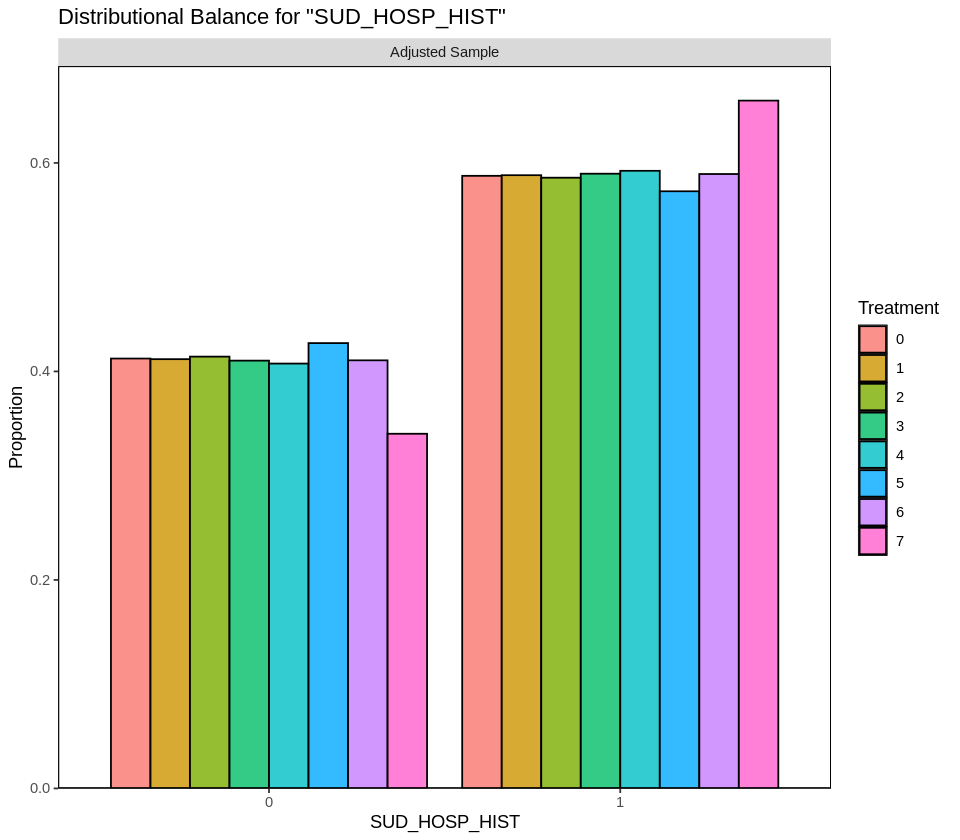

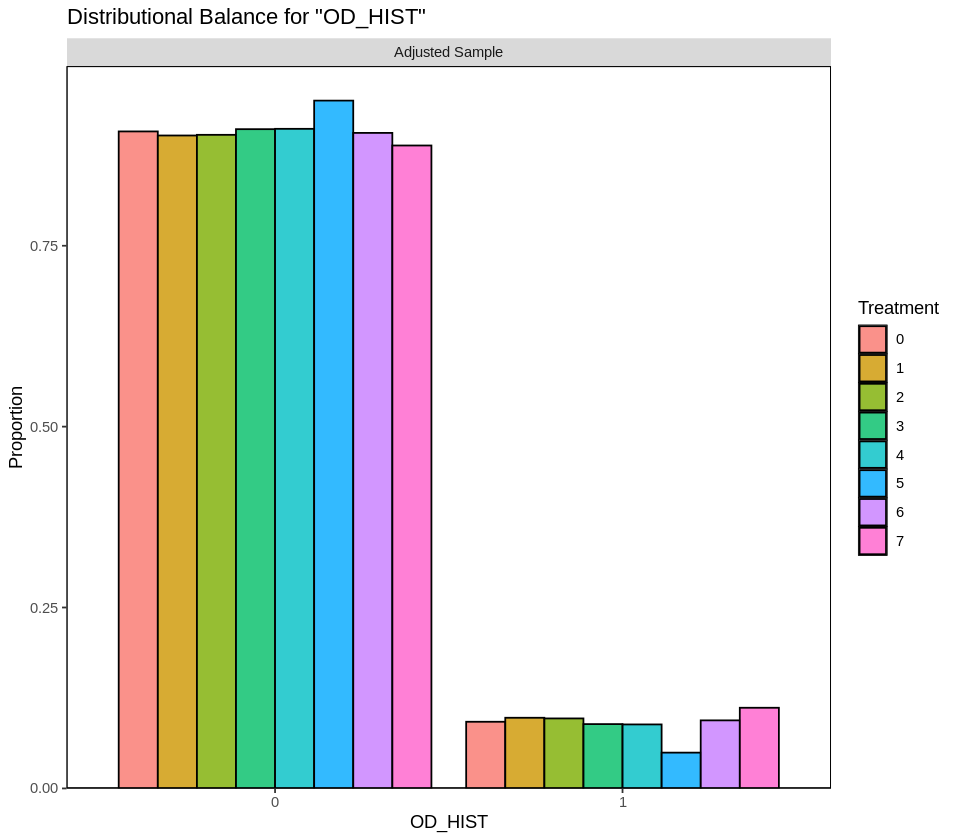

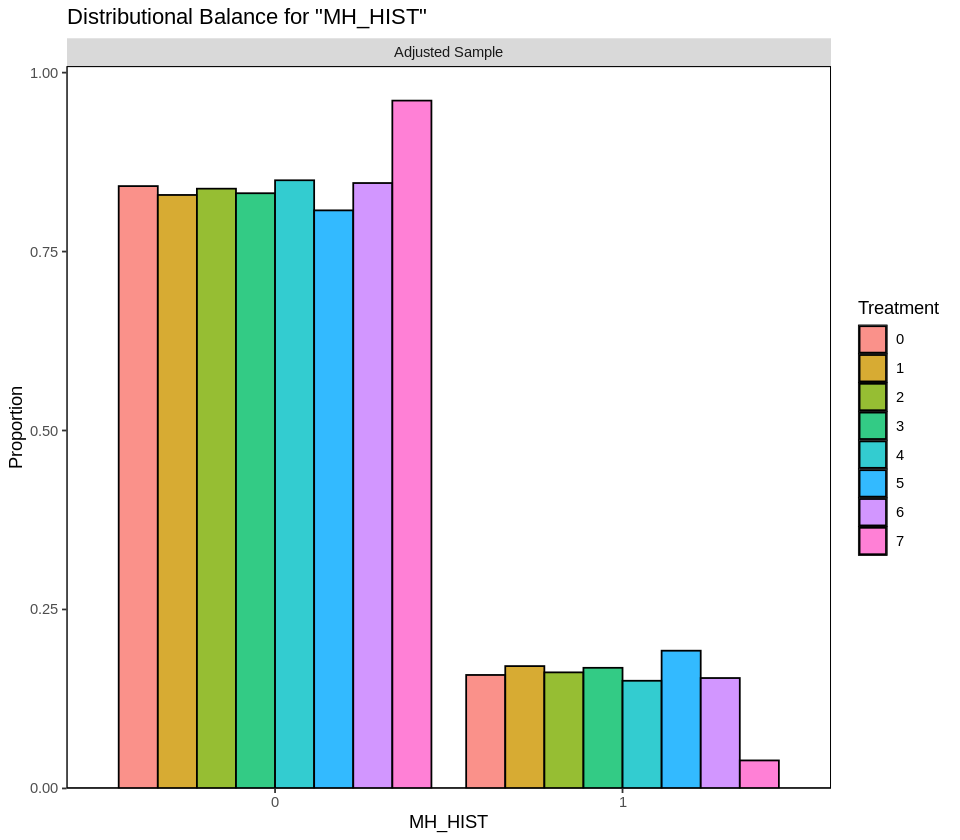


**Supplemental Table 14.**List of codes used to define anti-depressants

| **Code Type** | **Code(s)** |
| --- | --- |
| NDC | 00002300475, 00002323030, 00002400602, 00002400630, 00029320613,  00029320713, 00029320813, 00029321013, 00029321113, 00029321213  00029321313, 00029321548, 00029460613, 00029460713, 00049005001,  00049490030, 00049490041, 00049491030, 00049491041, 00049494023,  00049496030, 00054002213, 00054002313, 00054002413, 00054006258,  00054008246, 00093080701, 00093080756, 00093104201, 00093104219,  00093104301, 00093434656, 00093435601, 00093435619, 00093435693,  00093474001, 00093474101, 00093474105, 00093474150, 00093474201,  00093526256, 00093550456, 00093550556, 00093550656, 00093585001,  00093585005, 00093585101, 00093585105, 00093585201, 00093585205,  00093610812, 00093711498, 00093711556, 00093711598, 00093711656,  00093711698, 00093712198, 00093718801, 00093718810, 00093718856,  00093719801, 00093719805, 00093719856, 00121072104, 00121472105,  00143958005, 00143958009, 00143958105, 00143958109, 00143958209,  00143965405, 00143965430, 00143965505, 00143965530, 00143965609,  00143980801, 00143980901, 00172436370, 00185037101, 00185037201,  00185037301, 00247037200, 00247064407, 00378073401, 00378073501,  00378073593, 00378200305, 00378200393, 00378200405, 00378200493,  00378200593, 00378385577, 00378385610, 00378385677, 00378385710,  00378385777, 00378418605, 00378418701, 00378418705, 00378418805,  00378541028, 00378542028, 00378623101, 00378623105, 00378623201,  00378623205, 00378623301, 00378623305, 00378700110, 00378700193,  00378700210, 00378700293, 00378700310, 00378700393, 00378700410,  00378700493, 00378812101, 00378812105, 00378812701, 00440550400,  00440550600, 00440752107, 00440753807, 00440831530, 00440831630,  00440831730, 00456200501, 00456201001, 00456201063, 00456202001,  00456210108, 00456401001, 00456402001, 00456404001, 00456413008,  00555087154, 00574027930, 00713474001, 00713474005, 00713474101,  00713474105, 00713474201, 00713474205, 00777310402, 00777310502,  00777310507, 00777310530, 00777310730, 00777512058, 00781219331,  00781282201, 00781282210, 00781282301, 00781282310, 00781282401,  00832040211, 00832040330, 00904567661, 00904567761, 00904567861,  00904578461, 00904578561, 00904608461, 00904608561, 00904608661,  00904642661, 00904642761, 00904692461, 00904692561, 00904692661,  00904734661, 13107000501, 13107000505, 13107000601, 13107000605, 13107000701, 13107000705, 13107015405, 13107015430, 13107015490, 13107015505, 13107015530, 13107015590, 13107015599, 13107015630, 13107015690, 13107015699, 13107015705, 13107015730, 13107015790, 13107015799, 13668000401, 13668000501, 13668000530, 13668000550, 13668000601, 13668000610, 13668000901, 13668000905, 13668000909, 13668000930, 13668001001, 13668001005, 13668001006, 13668001030, 13668001101, 13668001105, 13668001108, 13668001130, 13668013501, 13668013510, 13668013601, 13668013605, 13668013610, 13668013701, 13668013705, 13668013710, 13668044301, 13668044391, 13668047301, 13668047330, 13668047391, 16590024930, 16590051430,  16714011201, 16714011301, 16714011401, 16714035101, 16714035201,  16714035202, 16714035203, 16714035301, 16714060102, 16714061101,  16714061105, 16714061106, 16714061201, 16714061204, 16714061205,  16714061206, 16714061301, 16714061304, 16714061305, 16714061306,  16714072002, 16729016801, 16729016817, 16729016901, 16729016917,  16729017001, 16729017017, 16729021510, 16729021515, 16729021615,  16729021616, 16729021715, 16729021716, 21695016030, 21695032100, 23155002910, 23155003001, 24658014110, 24658014210, 31722020601,  31722020701, 31722020705, 31722020710, 31722020801, 31722020805,  31722021205, 31722021230, 31722021290, 31722021305, 31722021330,  31722021390, 31722021405, 31722021430, 31722021490, 31722024990,  31722025090, 31722025190, 31722056924, 31722090401, 35356004530, 42291039601, 42291039610, 42291039710, 42291039801, 42291039850,  42543072505, 42543072510, 42543072601, 42543072605, 42543072610,  42543072701, 42543072705, 42543072730, 42806001901, 42806001910,  42806002001, 42806002010, 42806002101, 42806002110, 42858070303,  42858070503, 43063006360, 43063019790, 43547028010,  43547028011, 43547028110, 43547028111, 43547028210, 43547028211,  43547034703, 43547034709, 43547034711, 43547034750, 43547034803,  43547034809, 43547034811, 43547034850, 43547034903, 43547034909,  43547035003, 43547035009, 43547035011, 43547035050, 43547040903,  43598056601, 43598056630, 47781060030, 49884025011, 49884025111, 49884025311, 49884027711, 49884033501, 49884033511, 49884033601, 49884033611, 49884046811, 49884073401, 49884073510, 49884087201, 49884087205, 49909000530, 49999059730, 49999060115, 49999062730, 49999069030, 49999078030, 50111064701, 50111064702, 50111064703, 50111064801, 50111064802, 50111064803, 50111064844, 50228011330, 50228011410, 50228011430, 50228011501, 50228011505, 50228011530, 50268064215, 51079077420, 52343007330, 52343007430, 52343007490, 52343007499, 52343007630, 52343007690, 52427057630, 52427066430, 52427067230, 52427069130, 52817014090, 52817014100, 52817014190, 52959023350, 52959036012, 52959063804, 52959063930, 52959066530, 52959070430, 52959078730, 54458088910, 54458089210, 54458094410,  54458094510, 54458098010, 54458098110, 54458098810, 54458098910,  54766020101, 54766020401, 54838052340, 54838054070, 54838055170,  54868352600, 54868439400, 54868481700, 54868536500, 55111014701,  55111014801, 55111014810, 55111014930, 55111017505, 55111028448,  55111034201, 55111034205, 55111034230, 55111034301, 55111034305,  55111034330, 55111034401, 55111034405, 55111034430, 55289021522,  55289021630, 55289030814, 55289030830, 57664050713, 57664050788,  57664050813, 57664050818, 57664050888, 57664050913, 57664050918,  57664050988, 58016073100, 58016082890, 58016090700, 58160020100310,  58160020100320, 58160020100340, 58160020102020, 58160034100310, 58160034100320, 58160034100330, 58160040000110, 58160040000120, 58160040000140, 58160040000310, 58160040000320, 58160040000360, 58160040002020, 58160040006530, 58160060000310,  58160060000320, 58160060000330, 58160060000340, 58160060001820, 58160070100305, 58160070100310, 58160070100320, 58160070101320, 58864062815, 58864070730, 58864084930, 59746028001, 59746054305, 59746054401, 59746054405, 59746054601, 59746054605, 59762006701, 59762181201, 59762490001, 59762490002, 59762490003, 59762490004, 59762490005, 59762491001, 59762491003, 59762491004, 59762491005, 59762494001, 59762496001, 59762516001, 59762516003, 60429017310,  60429017410, 60429017510, 60505008301, 60505008302, 60505008304,  60505008401, 60505008402, 60505009701, 60505009702, 60505009704,  60505010101, 60505010102, 60505010104, 60505037401, 60505040205,  60505131603, 60505131703, 60505251903, 60505252001, 60505252003,  60505366303, 60505366403, 60505366503, 60505366603, 60505366803,  60505366903, 60505367003, 60505367303, 60505367403, 60505367503,  60505437703, 60505437803, 60505437903, 60505451703, 60505451803,  60505451903, 60505452003, 60687023101, 60687023111, 60687024201,  60687024477, 60687025301, 62175047032, 62175047132,  62175047141 62206040000320, 62332002231, 62332002291, 62332002331,  62332002391, 62332002430, 62332002431, 62332002491, 62332024230,  62332024231, 62332024330, 62332024331, 63304063201  63304063230, 63304068630, 63304068690, 63304068730, 63304068790,  63739088810, 63739096310, 63874057328, 63874059660, 63874060920,  63874076730, 64980040906, 65162005210, 65162005310  65162005350, 65162005403, 65162005410, 65162005450, 65162070588,  65862000501, 65862000505, 65862000601, 65862000605, 65862000701,  65862000705, 65862001105, 65862001130, 65862001201, 65862001205,  65862001230, 65862001301, 65862001305, 65862001330, 65862007424,  65862015505, 65862015599, 65862019201, 65862019299, 65862019301,  65862019305, 65862019399, 65862019401, 65862019405, 65862019430,  65862019499, 65862024824, 65862037301, 65862037305, 65862037401,  65862037405, 65862037501, 65862037505, 67544008071, 67544090615,  68001012900, 68001012903, 68001012904, 68001019600, 68001019703,  68001039908, 68001040000, 68001040104, 68001045508, 68001045600,  68001059100, 68001059200, 68001059300, 68071004060, 68084004401,  68084004501, 68084004601, 68084010101, 68084018001, 68084018101,  68084060501, 68084061701, 68084061801, 68084073701, 68180013501,  68180013601, 68180013701, 68180035103, 68180035106, 68180035109,  68180035202, 68180035205, 68180035206, 68180035209, 68180035302,  68180035305, 68180035306, 68180035309, 68180064506, 68180064606,  68180064706, 68180099706, 68180099801, 68180099806, 68180099901,  68180099906, 68382000105, 68382000106, 68382000116, 68382009705,  68382009706, 68382009710, 68382009716, 68382009801, 68382009805,  68382009806, 68382009810, 68382009816, 68382009905, 68382009906,  68382009910, 68382009916, 68645013054, 68645013154, 68645044770,  68645044870, 68645048870, 68645048970, 68645049901, 68645051454,  68645051954, 68645052054, 68645052154, 68645052254, 68645052354,  68645055954, 68645056954, 68645057054, 68645057154, 68968202001,  68968203001, 68968204001, 68968907503, 69097082207, 69097082212,  69097082307, 69097082312, 69097082407, 69097082412, 69097083302,  69097083305, 69097083312, 69097083402, 69097083412, 69097083502,  69097083512, 69097084705, 69097084805, 69097084905, 69367023510,  69367023601, 69367023610, 69367023701, 69367023705, 70954031910,  71610046460, 72241000711, 72241000811, 75834014830, 76282020610,  76282020690, 76282020710, 76282020790, 76282020810, 76282020890,  76282021260, 76282021290, 76282021305, 76282021318, 76282021330,  76282021390, 76282021401, 76282021405, 76282021418, 76282021430, 76282021460, 76282021490, 76282024910, 76282024990, 76282025010, 76282025030, 76282025090, 76282025110, 76282025190, 76282062810, 76282062910 |
| MMSL Synonym | 62559, 611247, 598032, 58827, 541662, 50414, 50415, 50416, 50417, 50418,  50419, 50420, 50421, 50422, 50423, 48028, 48031, 48032,  48033, 48034, 436212, 44667, 44668, 4493, 44963, 44964, 44965, 45067,  45068, 45069, 4517, 45602, 45706, 47422, 43187, 43215, 43289, 43385, 43386, 43387, 42334, 357240, 36437, 37675, 403969, 403971, 404408, 4091, 410584, 41163, 41267, 41287, 41430, 41456, 42085, 42110, 32030, 321988, 328269, 328270, 328294, 328295, 32937, 329430, 329431, 349332, 351161, 351165, 351249, 351250, 351285, 352272, 352273, 352741, 353108, 248642, 251201, 2532159, 2532163, 2556, 25714, 261287, 26360, 283672, 284591, 309313, 309314, 310384, 310385, 310386, 312241, 312244, 312938, 312940, 312941, 31331, 31341, 31354, 31372, 313989, 313990, 313995, 314199, 31667, 31670, 31697, 235830, 21742, 221078, 221789, 22634, 227224, 172243, 173151, 173152, 1738483, 1738495, 1738503, 1738511, 1738519, 1738803, 1738804, 1738805, 1738806, 1738807, 1738808, 18290, 18291, 18440, 18441, 18442, 18443, 18493, 18494, 186425, 187848, 18890, 18891, 19055, 19740, 200371, 20204, 205287, 205535, 207349, 207350, 208149, 208161, 211699, 211700, 212233, 213291, 213344, 213345, 215928, 136891, 13878, 14099, 1430128, 155137, 1554, 15682, 15683, 15684, 15685, 15856, 15857, 16096, 16097, 16461, 16462, 16555, 165727, 104849, 114228, 116775, 11806, 1190110, 12162, 12533, 12953, 647556, 725064, 725068, 725072, 82728, 83167, 85388, 85389, 861064, 861066 |
| MMSL Drug ID | d00236, d00880, d03157, d04332, d04812, d04917 |

**Supplemental Table 15.** Association between treatment and adverse healthcare outcomes (SUD-hospitalizations/ED visits, mental health crisis, all-drug overdose, relapse), among those with SUD a recent mental health condition (n=955,368)

|  | SUD hospitalization/ED visit | | Mental health crisis | | All-drug overdose | | Relapse | |
| --- | --- | --- | --- | --- | --- | --- | --- | --- |
|  | n^1^ (IR^2^) | aIRR^3^ (95% CI^4^) | n^1^ (IR^2^) | aIRR^3^ (95% CI^4^) | n^1^ (IR^2^) | aIRR^3^ (95% CI^4^) | n^1^ (IR^2^) | aIRR^3^ (95% CI^4^) |
| Overall | 514844 (229.66) | - | 717973 (320.27) | - | 146121 (65.18) | - | 169413 (75.57) | - |
| Individual treatments |  |  |  |  |  |  |  |  |
| OS^5^ |  |  |  |  |  |  |  |  |
| No | 506495 (238.97) | 1 [REF] | 697076 (328.89) | 1 [REF] | 141797 (66.9) | 1 [REF] | 166874 (78.73) | 1 [REF] |
| Yes | 8349 (68.28) | **0.38 (0.36, 0.39)** | 20897 (170.91) | **0.56 (0.54, 0.58)** | 4324 (35.36) | **0.59 (0.55, 0.64)** | 2539 (20.77) | **0.35 (0.33, 0.37)** |
| Anesthetics |  |  |  |  |  |  |  |  |
| No | 506495 (238.97) | 1 [REF] | 695134 (318.17) | 1 [REF] | 144505 (66.14) | 1 [REF] | 165799 (75.89) | 1 [REF] |
| Yes | 8349 (68.28) | **0.89 (0.86, 0.93)** | 22839 (400.7) | **0.95 (0.91, 0.98)** | 1616 (28.35) | **0.55 (0.49, 0.63)** | 3614 (63.41) | **0.85 (0.80, 0.89)** |
| Psychedelics |  |  |  |  |  |  |  |  |
| No | 513595 (229.72) | 1 [REF] | 715921 (320.22) | 1 [REF] | 145873  (65.25) | 1 [REF] | 169013 (75.6) | 1 [REF] |
| Yes | 1249 (206.88) | **0.88 (0.78, 0.98)** | 2052 (339.88) | *0.92 (0.82, 1.03)* | 202 (33.46) | **0.57 (0.39, 0.82)** | 400 (66.25) | **0.87 (0.76, 0.99)** |
| Sum of treatments |  |  |  |  |  |  |  |  |
| 0 | 494761 (239.9) | 1 [REF] | 673963 (326.8) | 1 [REF] | 140179 (67.97) | 1 [REF] | 163127 (79.10) | 1 [REF] |
| 1 | 19327 (111.29) | **0.56 (0.54, 0.57)** | 42242 (243.23) | **0.70 (0.68, 0.72)** | 5726 (32.97) | **0.56 (0.53, 0.60)** | 6022 (34.68) | **0.52 (0.50, 0.54)** |
| 2 | 748 (132.4) | **0.60 (0.52, 0.70)** | 1758 (311.18) | **0.69 (0.61, 0.78)** | 209 (37.00) | **0.68 (0.49, 0.95)** | 261 (46.20) | **0.63 (0.53, 0.76)** |
| 3 | 8 (71.62) | 0.35 (0.09, 1.37) | 10 (89.52) | 0.22 (0.04, 1.16) | 7 (62.67) | 0.96 (0.18, 5.90) | 3 (26.86) | 0.41 (0.08, 2.21) |
| Combinations of treatments |  |  |  |  |  |  |  |  |
| None | 494761 (239.9) | 1 [REF] | 673963 (326.8) | 1 [REF] | 140179 (67.97) | 1 [REF] | 163127 (79.10) | 1 [REF] |
| OS only | 8033 (67.48) | **0.37 (0.35, 0.39)** | 20014 (168.12) | **0.55 (0.53, 0.57)** | 4176 (35.08) | **0.58 (0.54, 0.63)** | 2420 (20.33) | **0.34 (0.32, 0.36)** |
| Anesthetics only | 10500 (204.55) | **0.88 (0.85, 0.92)** | 21098 (411) | **0.94 (0.91, 0.98)** | 1402 (27.31) | **0.51 (0.45, 0.59)** | 3351 (65.28) | **0.83 (0.79, 0.87)** |
| Psychedelics only | 794 (241.11) | **0.89 (0.77, 0.99)** | 1130 (343.14) | *0.92 (0.79, 1.08)* | 148 (44.94) | **0.58 (0.40, 0.86)** | 251 (76.22) | *0.86 (0.72, 1.04)* |
| OS and anesthetics | 301 (99.77) | **0.49 (0.39, 0.61)** | 846 (280.43) | **0.61 (0.51, 0.73)** | 139 (46.07) | 0.88 (0.58, 1.34) | 115 (38.12) | **0.56 (0.43, 0.73)** |
| OS and psychedelics | 7 (72.24) | **0.23 (0.05, 0.98)** | 27 (278.65) | 0.60 (0.22, 1.63) | 2 (20.64) | 0.20 (0.01, 6.26) | 1 (10.32) | 0.10 (0.01, 1.85) |
| Anesthetics and psychedelics | 440 (173.53) | **0.79 (0.65, 0.95)** | 885 (349.02) | *0.86 (0.72, 1.03)* | 68 (26.82) | **0.48 (0.27, 0.87)** | 145 (57.18) | **0.79 (0.62, 0.97)** |
| OS, anesthetics, and psychedelics | 8 (71.62) | 0.36 (0.09, 1.42) | 10 (89.52) | 0.23 (0.04, 1.19) | 7 (62.67) | 0.97 (0.19, 6.01) | 3 (26.86) | 0.43 (0.08, 2.28) |

**1** count of events occurring within >=3 months and <=27 months after index SUD (allowing a two-year follow-up period)

**2** incidence rate (per 10,000 person-months)

**3** adjusted incidence rate ratio, via Quasipoisson regression; sample balanced on treatment status (separately for each individual, summation, and combination) via logistic/multinomial regression propensity score weighting with average treatment effect in population (ATE) estimand, by age, gender, race/ethnicity, marital status, census division, metropolitan status, rural status, year of index SUD, insurance, comorbidity, index SUD count, number of mental health conditions history, chronic pain history, problems related to lifestyle history, adverse socioeconomic/psychosocial determinants of health history, anesthetics history, antidepressants prescription, benzodiazepine prescription, procedure history, MSUD, SUD hospitalization history, mental health crisis history, all-drug overdose history, relapse history (and OS, anesthetics at baseline, psychedelics at baseline with respective removal given the treatment chosen of interest for balancing), doubly adjusted for residual imbalance

**4** confidence interval **5** outpatient SUD services

**Supplemental Table 16.** Association between treatment (MAT, anesthetics, psychedelics) and adverse healthcare outcomes (SUD-hospitalizations/ED visits, mental health crisis, all-drug overdose, relapse), among those with SUD

|  | SUD hospitalization/ED visit | | Mental health crisis | | All-drug overdose | | Relapse | |
| --- | --- | --- | --- | --- | --- | --- | --- | --- |
|  | n^1^ (IR^2^) | aIRR^3^ (95% CI^4^) | n^1^ (IR^2^) | aIRR^3^ (95% CI^4^) | n^1^ (IR^2^) | aIRR^3^ (95% CI^4^) | n^1^ (IR^2^) | aIRR^3^ (95% CI^4^) |
| Overall | 1498433 (199.71) | - | 1036281 (138.12) | - | 372328 (49.62) | - | 466777 (62.21) | - |
| Individual treatments |  |  |  |  |  |  |  |  |
| MAT |  |  |  |  |  |  |  |  |
| No | 1411513 (205.83) | 1 [REF] | 949959 (138.52) | 1 [REF] | 347679 (50.80) | 1 [REF] | 440593 (64.25) | 1 [REF] |
| Yes | 86920 (134.71) | **0.74 (0.73, 0.75)** | 86322 (133.78) | **0.76 (0.73, 0.79)** | 24649 (38.20) | **0.71 (0.69, 0.73)** | 26184 (40.58) | **0.73 (0.72, 0.74)** |
| Anesthetics |  |  |  |  |  |  |  |  |
| No | 1467776 (200.46) | 1 [REF] | 1006136 (137.42) | 1 [REF] | 369346 (50.44) | 1 [REF] | 457645 (62.50) | 1 [REF] |
| Yes | 30657 (169.25) | **0.86 (0.84, 0.88)** | 30145 (166.42) | **0.89 (0.87, 0.91)** | 2982 (16.46) | **0.44 (0.40, 0.48)** | 9132 (50.41) | **0.81 (0.78, 0.84)** |
| Psychedelics |  |  |  |  |  |  |  |  |
| No | 1495223 (199.78) | 1 [REF] | 1033615 (138.11) | 1 [REF] | 371801 (49.68) | 1 [REF] | 465862 (62.25) | 1 [REF] |
| Yes | 3210 (171.17) | **0.81 (0.76, 0.86)** | 2666 (142.16) | **0.86 (0.79, 0.94)** | 449 (23.94) | **0.56 (0.46, 0.71)** | 915 (48.79) | **0.76 (0.69, 0.84)** |
| Sum of treatments |  |  |  |  |  |  |  |  |
| 0 | 1381282 (206.63) | 1 [REF] | 921904 (137.91) | 1 [REF] | 344831 (51.58) | 1 [REF] | 431660 (64.57) | 1 [REF] |
| 1 | 113617 (143.46) | **0.75 (0.74, 0.76)** | 109773 (138.61) | **0.77 (0.76, 0.78)** | 26901 (33.97) | **0.66 (0.64, 0.68)** | 34034 (42.97) | **0.72 (0.71, 0.73)** |
| 2 | 3432 (135.54) | **0.69 (0.64, 0.73)** | 4452 (175.82) | **0.76 (0.71, 0.81)** | 570 (22.51) | **0.53 (0.43, 0.65)** | 1052 (41.55) | **0.67 (0.61, 0.73)** |
| 3 | 102 (121.23) | **0.62 (0.43, 0.90)** | 152 (180.66) | 0.80 (0.56, 1.14) | 26 (30.90) | 0.70 (0.27, 1.83) | 31 (36.85) | *0.61 (0.36, 1.01)* |
| Combinations of treatments |  |  |  |  |  |  |  |  |
| None | 1381282 (206.73) | 1 [REF] | 921904 (137.91) | 1 [REF] | 344831 (51.58) | 1 [REF] | 431660 (64.57) | 1 [REF] |
| MAT only | 84462 (134.98) | **0.72 (0.71, 0.73)** | 82727 (132.21) | **0.74 (0.73, 0.75)** | 24161 (38.61) | **0.70 (0.68, 0.73)** | 25426 (40.63) | **0.70 (0.69, 0.71)** |
| Anesthetics only | 27293 (174.88) | **0.86 (0.84, 0.88)** | 25742 (164.94) | **0.88 (0.85, 0.90)** | 2445 (15.67) | **0.40 (0.36, 0.44)** | 8099 (51.89) | **0.80 (0.77, 0.82)** |
| Psychedelics only | 1862 (183.18) | **0.75 (0.68, 0.81)** | 1304 (128.29) | **0.79 (0.70, 0.89)** | 295 (29.02) | **0.45 (0.34, 0.60)** | 509 (50.08) | **0.66 (0.59, 0.75)** |
| MAT and anesthetics | 2186 (124.39) | **0.66 (0.61, 0.71)** | 3242 (184.48) | **0.75 (0.70, 0.81)** | 403 (22.93) | **0.56 (0.44, 0.71)** | 677 (38.52) | **0.64 (0.57, 0.71)** |
| MAT and psychedelics | 170 (155.07) | **0.65 (0.49, 0.86)** | 201 (183.34) | 0.93 (0.68, 1.27) | 59 (53.81) | 0.74 (0.39, 1.39) | 50 (45.61) | **0.62 (0.42, 0.93)** |
| Anesthetics and psychedelics | 1076 (161.78) | **0.79 (0.71, 0.89)** | 1009 (151.71) | **0.78 (0.68, 0.89)** | 108 (16.24) | **0.36 (0.22, 0.57)** | 325 (48.86) | **0.76 (0.65, 0.89)** |
| MAT, anesthetics, and psychedelics | 102 (121.23) | **0.63 (0.44, 0.91)** | 152 (180.66) | 0.81 (0.56, 1.15) | 26 (30.90) | 0.69 (0.27, 1.78) | 31 (36.85) | *0.61 (0.37, 1.01)* |

**1** count of events occurring within >=3 months and <=27 months after index SUD (allowing a two-year follow-up period)

**2** incidence rate (per 10,000 person-months)

**3** adjusted incidence rate ratio, via Quasipoisson regression; sample balanced on treatment status (separately for each individual, summation, and combination) via logistic/multinomial regression propensity score weighting with average treatment effect in population (ATE) estimand, by age, gender, race/ethnicity, marital status, census division, metropolitan status, rural status, year of index SUD, insurance, comorbidity, index SUD count, number of mental health conditions history, chronic pain history, problems related to lifestyle history, adverse socioeconomic/psychosocial determinants of health history, anesthetics history, benzodiazepine prescription, procedure history, SUD hospitalization history, mental health crisis history, all-drug overdose history, relapse history (and MAT, anesthetics at baseline, psychedelics at baseline with respective removal given the treatment chosen of interest for balancing), doubly adjusted for residual imbalance

**4** confidence interval
